# Mild and efficient synthesis of carbamates using dioxazolones as bench-stable isocyanate surrogates: application in AChE-inhibiting agent development

**DOI:** 10.1039/d6ra00983b

**Published:** 2026-03-24

**Authors:** Yinxin Wu, Xiaodan Liu, Fangfang Zuo, Yulu Ding, Jiasheng Kang, Jianping Wu, Wenjian Tang, Jing Zhang

**Affiliations:** a School of Pharmacy, Anhui Medical University Hefei 230032 China ahmupharm@ahmu.edu.cn; b Anhui Province Key Laboratory of Green Manufacturing in Phosgene Industry, Anhui Guangxin Agrochemical Co., Ltd Guangde 242200 China 1026171396@qq.com; c Anhui Province Key Laboratory of Occupational Health, Anhui No.2 Provincial People's Hospital Hefei 230041 China hfzj2552@163.com

## Abstract

A practical method for carbamate synthesis was developed using 3-substituted dioxazolones as bench-stable isocyanate surrogates. Prepared from hydroxamic acids using triphosgene, these dioxazolones underwent base-promoted decarboxylation to generate isocyanates *in situ*. The optimized protocol (MeCN, Cs_2_CO_3_, 70 °C) enabled rapid (20 min), high-yielding access to diverse carbamates, including commercial insecticides propoxur and carbofuran in both milligram and gram scales. The carbamate-forming step avoided direct handling of volatile isocyanates, generates CO_2_ as the only byproduct, and exhibited broad substrate scope, affording a 30-compound library. Biological evaluation identified several potent acetylcholinesterase (AChE) inhibitors, with series-5 demonstrating nanomolar-level activity. Molecular docking provided structural insights into structure–activity relationships and potential determinants of insect *versus* mammalian AChE selectivity. This work offered a scalable route to carbamate agents and a valuable compound library for AChE-targeted discovery.

## Abstract 2

A practical method for carbamate synthesis was developed using 3-substituted dioxazolones as bench-stable isocyanate surrogates.

## Graphical abstract

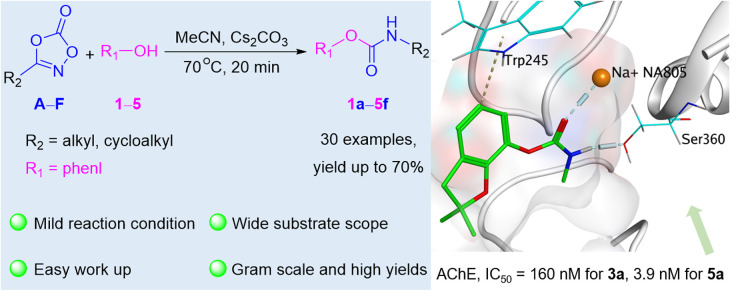

## 1 Introduction

**Fig. 1:**
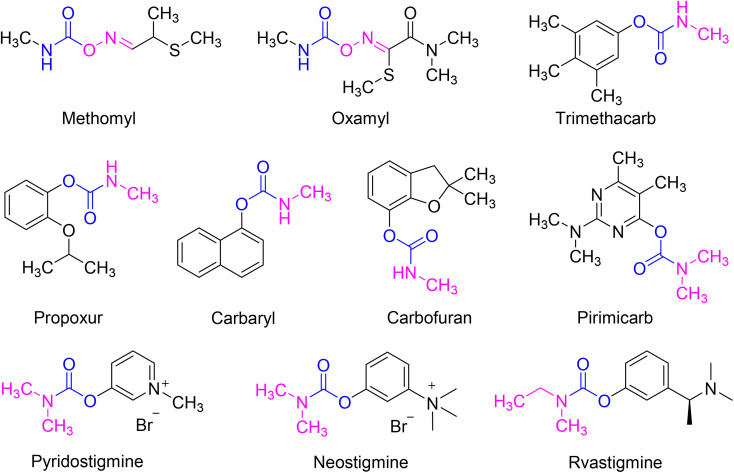
Representative insecticides and drugs containing carbamate scaffold.

Carbamate compounds constitute a vital class of agrochemicals, valued for their potent insecticidal activity and broad-spectrum efficacy in integrated pest management.^1–3^ The primary biological target of carbamate insecticides is acetylcholinesterase (AChE), a key enzyme in the insect nervous system. By carbamylating the active-site serine, these carbamates disrupt acetylcholine hydrolysis, leading to neurotransmitter accumulation, synaptic dysfunction, and ultimately insect paralysis and death.^4–7^ Prominent examples such as carbofuran, carbaryl, and propoxur underscore the agricultural importance of this scaffold (Fig. 1), combining effectiveness with relatively shorter environmental persistence compared to organophosphates.^2,8,9^ Also, carbamate-based drugs exhibit diverse therapeutic effects including neostigmine, pyridostigmine and rivastigmine (Fig. 1).^10–12^ As versatile intermediates in organic synthesis, carbamates can either be converted into amines or directly find diverse applications in the chemical industry.^13–17^

**Fig. 2:**
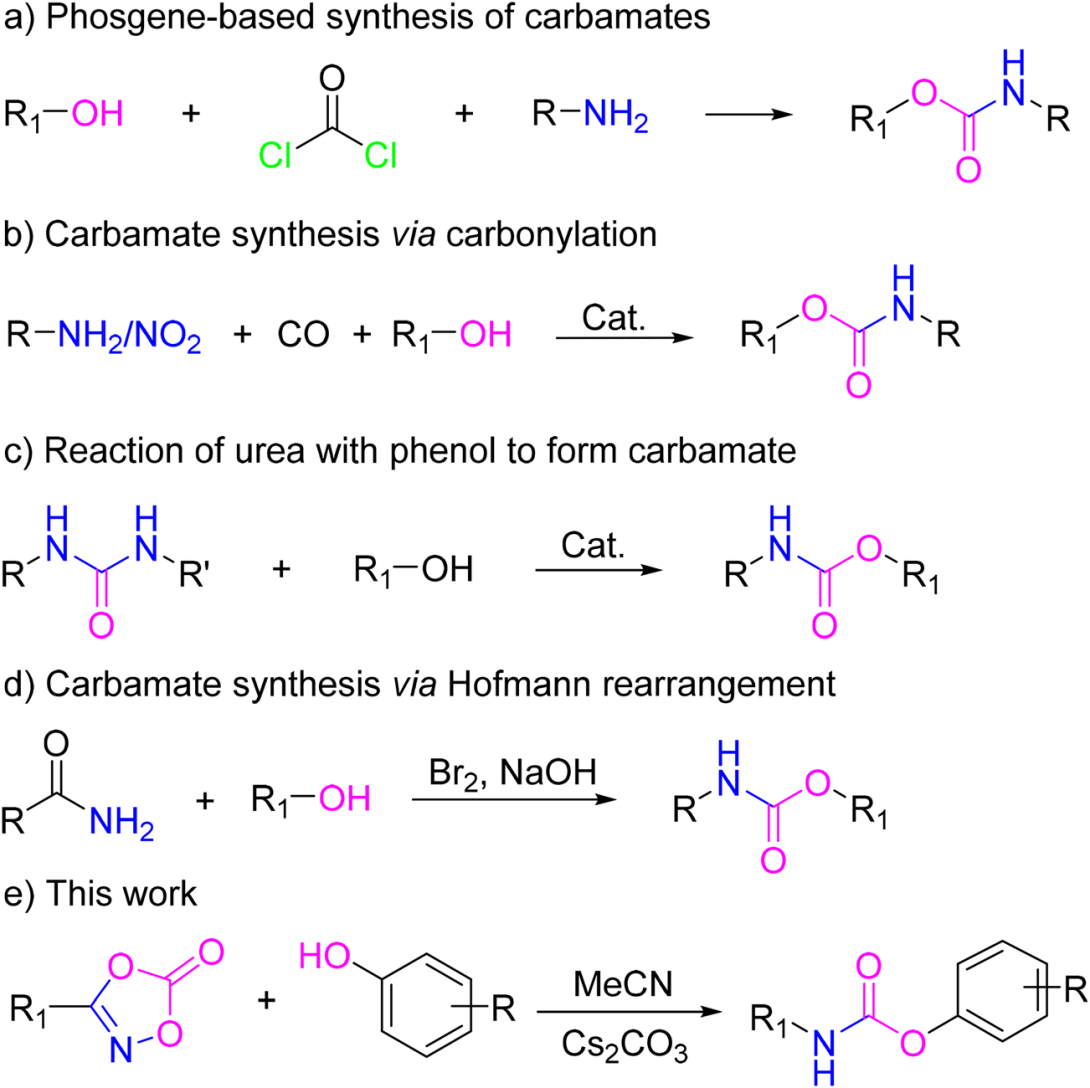
Strategy for the synthesis of carbamates.

Despite their utility, conventional industrial synthesis of carbamates often relies on methodologies with significant drawbacks. As shown in Fig. 2, the most prevalent routes involve the use of highly toxic and corrosive phosgene or its derivatives (Fig. 2a),^18^ transition-metal-catalyzed carbonylation reactions that require high-pressure equipment (Fig. 2b),^19,20^ alcoholysis of ureas, which can suffer from limited substrate scope and efficiency (Fig. 2c),^21^ or the Hofmann rearrangement (Fig. 2d).^22,23^ These methods conflict with the growing imperative for sustainable chemical processes in chemical manufacturing. Consequently, the development of safe, efficient, and condition mild synthetic strategies remains a pressing objective.^24^

In recent years, stable isocyanate surrogates have emerged as promising alternatives to volatile and toxic free isocyanates. Amongst them, 3-substituted dioxazolones have gained attention as bench-stable precursors that undergo controlled decarboxylation to generate isocyanates *in situ* under mild conditions (Fig. 2e).^25,26^ This approach mitigates the hazards associated with the isolation and use of volatile isocyanates. Pioneering work demonstrated the thermolysis and cobalt-catalysis of dioxazolones to isocyanates,^27,28^ while subsequent studies effectively leveraged these intermediates for the synthesis of ureas and thiocarbamates.^29,30^ Inspired by these advances, we hypothesized that 3-substituted dioxazolones could serve as ideal precursors for a benign and general synthesis of carbamate derivatives through their reaction with readily available phenols and alcohols.

In this study, a mild, efficient method was reported for the preparation of carbamates (Fig. 2). By systematically optimizing the reaction conditions, we established a robust protocol utilizing 3-substituted dioxazolones as isocyanate surrogates in the presence of a base. This method operated under mild reaction conditions, affords high yields with minimal impurities, and is well-suited for the synthesis of carbamates. Utilizing this optimized approach, we designed and synthesized a focused library of carbamates. The synthesized compounds were evaluated for AChE inhibitory activity to explore their potential as AChE-targeted agents and to elucidate SARs relevant to AChE-inhibited design.

## 2 Materials and methods

### 2.1. Reagents and instruments

All chemicals and solvents were purchased from commercial suppliers (Energy Chemicals, Shanghai, China) and used without further purification unless otherwise noted. Reactions were performed under ambient atmosphere unless specified. Reaction progress was monitored by thin-layer chromatography (TLC) on silica gel plates with UV detection at 254 nm. Melting points were measured using an XT4MP apparatus (Taike Instruments, Beijing, China) and are uncorrected. Nuclear magnetic resonance (NMR) spectra were recorded on a 500 MHz spectrometer for ^1^H and 126 MHz for ^13^C, with CDCl_3_ as the solvent. Chemical shifts are reported in parts per million (ppm) relative to tetramethylsilane (TMS). Coupling constants (*J*) are given in Hertz (Hz), and signal multiplicities are denoted as s (singlet), d (doublet), t (triplet), and m (multiplet). High-resolution mass spectrometry (HRMS) data were acquired using an Agilent 1260-6221 TOF mass spectrometer.

### 2.2. Synthesis of 3-substituted dioxazolones

3-Substituted dioxazolones (A–F) were synthesized *via* triphosgene-mediated cyclization of the corresponding hydroxamic acids. In a typical reaction, the hydroxamic acid (10.0 mmol) was treated with triphosgene (4.0 mmol) in anhydrous CH_2_Cl_2_ (20 mL) in the presence of NaHCO_3_ (10 mmol). The mixture was stirred at 0 °C for 1 h and then at room temperature for 6–8 h. After filtration and concentration under reduced pressure, the crude dioxazolone was obtained and used without purification in subsequent carbamate synthesis.

### 2.3. General procedure for carbamate synthesis using dioxazolones

To a solution of phenol (2.0 mmol) in anhydrous acetonitrile (5 mL) were added the appropriate 3-substituted dioxazolone (2.0 equiv.) and Cs_2_CO_3_ (0.5 equiv.). The reaction mixture was stirred at 70 °C for 20 min. After completion (monitored by TLC), the mixture was cooled to room temperature, diluted with EtOAc (30 mL), and washed with saturated brine (2 × 30 mL). The organic phase was dried over anhydrous Na_2_SO_4_, filtered, and concentrated under reduced pressure. The crude product was purified by flash column chromatography on silica gel using petroleum ether/EtOAc (15 : 1) as eluent. Further recrystallization from petroleum ether/EtOAc (30 : 1) afforded the pure carbamate derivatives 1a–5f. Characterization data of 1a–5f including ^1^H-NMR, ^13^C-NMR, and HRMS and their spectra were listed in SI.

### 2.4. Gram-scale synthesis of propoxur and carbofuran

The optimized protocol was applied to a gram-scale synthesis. 3-Methyl-1,4,2-dioxazol-5-one (1, 10 mmol) and the corresponding phenol (5 mmol) were combined with Cs_2_CO_3_ (0.5 equiv.) in MeCN (50 mL) and stirred at 70 °C for 20 min. Work-up as described above afforded propoxur and carbofuran in 72% and 78% yield, respectively.

### 2.5. Acetylcholinesterase (AChE) inhibition assays

Inhibitory activities against *Electrophorus electricus* AChE (eeAChE) were determined using a modified Ellman's method. The reaction system contained 0.036 U mL^−1^ of AChE (C3389 e500 UN; Sigma) and synthesized compounds (0.002–20 µM) dissolved in 0.1 M phosphate buffer (pH = 8.0). The mixture was pre-incubated at 37 °C for 20 min. Subsequently, 0.35 mM of acetylthiocholine iodide (ATCHI, Sigma, A5751) was added as a substrate, along with 0.35 mM of the developer 5,5′-dithiobis (2-nitrobenzoic acid; DTNB, Sigma, D8130). The plate was then incubated at 37 °C until appropriate color development was observed.

The absorbance of each well was measured at a wavelength of 410 nm using a Tecan Infinite M Nano multifunctional microplate reader. The half-maximal inhibitory concentration (IC_50_) for each compound was calculated by nonlinear regression analysis using GraphPad Prism software (version 8.0.1). The following controls were included in each experiment: a positive control (containing buffer, enzyme, DTNB, and substrate, but no inhibitor) and a blank control (containing buffer, DTNB, and substrate, but no enzyme). All assays were independently repeated three times.

### 2.6. Molecular docking

To elucidate the molecular basis of AChE inhibition and inform the design of selective insecticides, molecular docking studies were performed using the Molecular Operating Environment (MOE) software. The crystal structures used include electric eel-derived and human-derived acetylcholinesterases: *Tetronarce californica* acetylcholinesterase (eeAChE, PDB: 6TT0) and human acetylcholinesterase (*h*AChE, PDB: 7D9O).

Structural alignment and binding pocket similarity among the receptors were analyzed using the superposition tools in MOE. Rigid docking was carried out, and the GBVI/WSA dG scoring function was applied to evaluate binding poses. The conformation with the lowest free energy of binding was selected for further analysis.

### 2.7. Cytotoxicity assays

Cytotoxicity was evaluated using the CCK-8 assay on AML-12 and HepG2 cell lines. HepG2 cells were cultured in DMEM medium supplemented with 10% fetal bovine serum (FBS), 100 U mL^−1^ penicillin, and 100 µg mL^−1^ streptomycin. AML-12 cells were maintained in DMEM/F12 medium (Gibco, USA) containing 10% FBS, 50 U mL^−1^ penicillin, 50 µg mL^−1^ gentamicin, 1% sodium pyruvate, and 1% non-essential amino acids. All cell lines were incubated at 37 °C in a humidified atmosphere of 5% CO_2_.

Cells were seeded in a 96-well plate and incubated for 24 h. This was followed by treatment with carbamate derivatives (20 µM) for 24 h. After incubation, CCK-8 solution was added to each well, and absorbance was detected at 450 nm after 1.5 h using a Tecan Infinite M Nano multifunction microplate reader. Cell viability was determined from three independent experiments, each performed in triplicate. The absorbance of the blank control (cells with fresh medium only) was set as 100% viability. Cell viability was calculated using the following formula: Cell viability (%) = [OD_450_ (compound)/OD_450_ (blank)] × 100%.

## 3 Results and discussion

### 3.1. Optimization of reaction conditions for carbamate formation

Given the importance of carbamates in agrochemical applications, we sought to develop a mild and efficient synthetic route using 3-substituted dioxazolones as safe isocyanate precursors. Initial studies focused on the cyclization of hydroxamic acids to the corresponding dioxazolones using triphosgene, which proceeded in excellent yield. The resulting 3-cyclopentyl-1,4,2-dioxazol-5-one (D) was selected as a model substrate for reaction optimization.

To establish an environmentally benign and high-yielding process, the coupling of 2,2-dimethyl-2,3-dihydrobenzofuran-7-ol (5)—a structural motif found in several commercial insecticides—with 3-cyclopentyl-1,4,2-dioxazol-5-one (D) was examined. Preliminary screening under Pfizer's green chemistry guidelines identified acetonitrile (MeCN) as the optimal solvent, minimizing side-product formation compared to DMSO or acetone (Table 1).

**Table 1:** Optimization of carbamate synthesis from dioxazolone D and phenol 5

Entry	Solvent	Base	D (equiv.)	Temp. (°C)	Time (h)	Yield (%)
1	Acetone	K_2_CO_3_	1	30	8	25
2	MeCN	K_2_CO_3_	1	30	8	22
3	DMSO	K_2_CO_3_	1	30	8	5
4	MeCN	Cs_2_CO_3_	1	30	8	29
5	MeCN	Cs_2_CO_3_	1.5	30	8	47
6	MeCN	Cs_2_CO_3_	2	30	8	59
7	MeCN	Cs_2_CO_3_	3	30	8	61
8	MeCN	Cs_2_CO_3_	4	30	8	63
9	MeCN	Cs_2_CO_3_ (0.2 eq.)	2	30	12	55
10	MeCN	Cs_2_CO_3_ (0.5 eq.)	2	30	8	58
11	MeCN	Cs_2_CO_3_ (2.0 eq.)	2	30	2	57
12	MeCN	Cs_2_CO_3_ (0.5 eq.)	2	60	1	73
13	MeCN	Cs_2_CO_3_ (0.5 eq.)	2	70	20 (min)	86
14	MeCN	Cs_2_CO_3_ (0.5 eq.)	2	80	7 (min)	74

Base selection proved critical for suppressing urea byproduct formation, commonly arising from isocyanate hydrolysis. Among inorganic bases tested, Cs_2_CO_3_ afforded the best balance of reactivity and selectivity. Further optimization showed that employing 2.0 equiv. of dioxazolone D and 0.5 equiv. of Cs_2_CO_3_ in MeCN at 70 °C for 20 min gave the target carbamate 5d in 86% yield, with minimal byproducts and short reaction time—factors advantageous for potential scale-up in pesticide manufacturing.

### 3.2. Substrate scope: synthesis of carbamate analogues as pesticides

To assess the utility of this method in pesticide chemistry, a panel of phenolic substrates resembling commercial carbamate agrochemicals was selected, including *m*-cresol (1), 2-(sec-butyl)phenol (2), 2-isopropoxyphenol (3), naphthol (4), and 2,2-dimethyl-2,3-dihydrobenzofuran-7-ol (5). Correspondingly, a series of 3-substituted dioxazolones (A–F) were prepared from readily available hydroxamic acids using triphosgene-mediated cyclization.

**Scheme 1:**
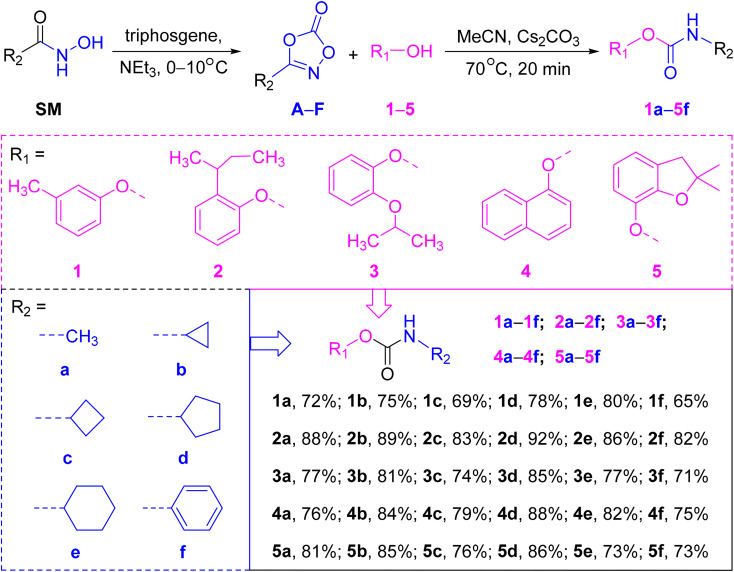
Synthesis of carbamates and their structures.

Under the optimized conditions, all 30 carbamate derivatives (1a–5f) were synthesized in good to excellent yields (Scheme 1). Notably, substrates bearing electron-donating groups (*e.g.*, series 2) exhibited enhanced reactivity, likely due to facilitated nucleophilic attack on the intermediate isocyanate. Importantly, several of the products, including 1a (metolcarb), 2a (fenobucarb), 3a (propoxur), 4a (carbaryl), and 5a (carbofuran), are known pesticides, confirming the method's applicability in agrochemical synthesis.

### 3.3. Proposed mechanism

**Scheme 2:**
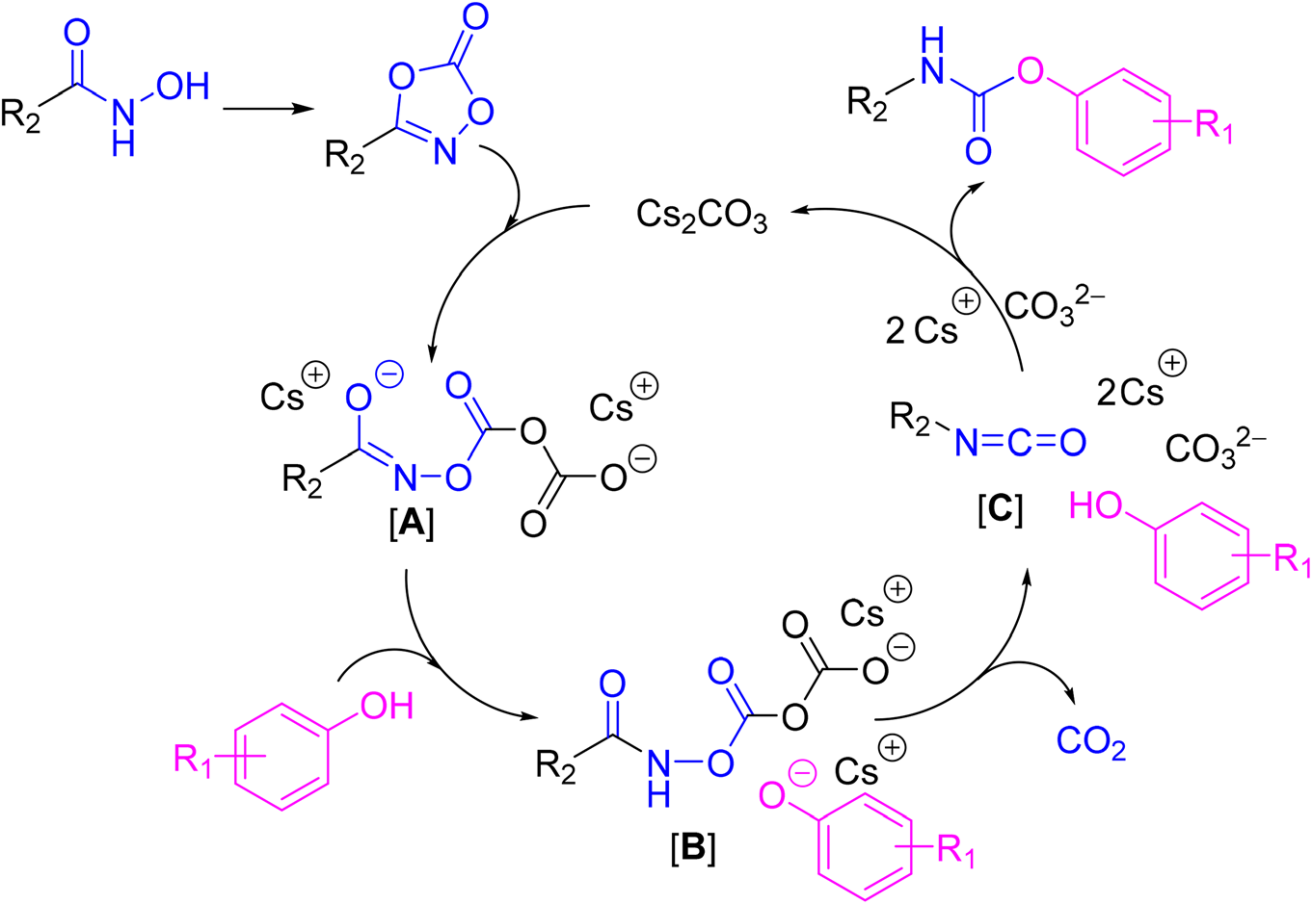
Possible base-promoted reaction mechanism for carbamate formation.

According to proposed reaction mechanism for the base-promoted formation of unsymmetrical phenylurea using 3-substituted dioxazolone,^27^ the base-promoted mechanism of *O*-carbonic anhydride intermediate is proposed (Scheme 2). In the presence of cesium carbonate (Cs_2_CO_3_) as an alkaline salt initiator, dioxazolone undergoes ring-opening to form the *O*-carbonic anhydride intermediate [A]. Under mild heating, intermediate [A] undergoes proton transfer to afford the activated hydroxamate [B], which then decarboxylates spontaneously to generate the isocyanate intermediate [C]. Nucleophilic addition by phenol affords the corresponding carbamate. Cs_2_CO_3_ acts as a catalyst in this reaction and participates in the catalytic cycle.

### 3.4. Gram-scale synthesis of commercial insecticides

**Scheme 3:**
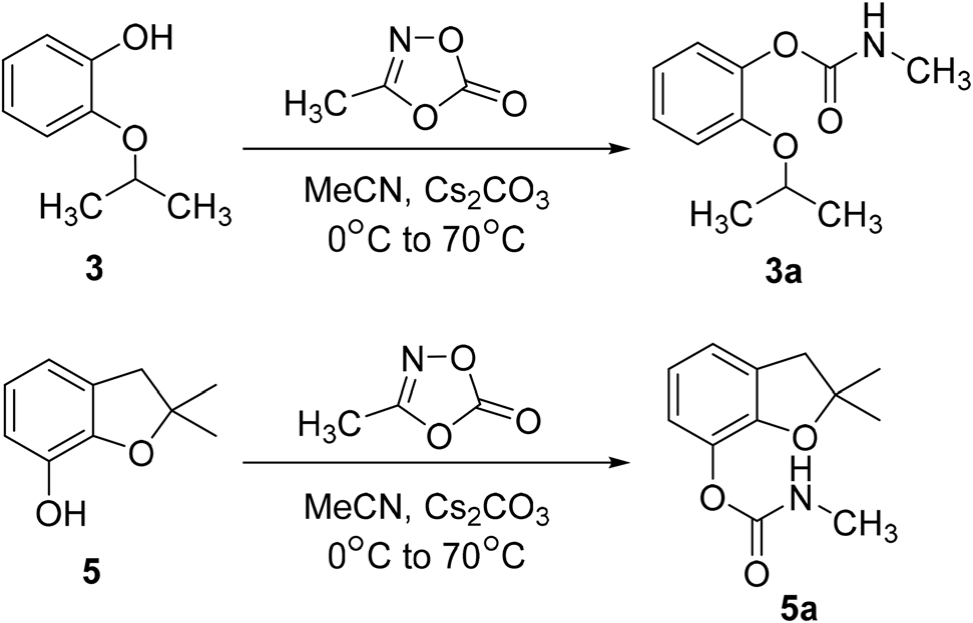
Synthesis of propoxur and carbofuran.

To demonstrate practical utility, the protocol was applied to the gram-scale synthesis of two important carbamate insecticides: propoxur and carbofuran. Using 3-methyl-1,4,2-dioxazol-5-one (A) as a methyl isocyanate surrogate, the corresponding carbamates were obtained in 72% and 78% yield, respectively, with CO_2_ as the sole byproduct (Scheme 3). This efficient, simplified procedure highlights the potential of this methodology for the sustainable and scalable production of carbamate-based pesticides.

### 3.5. Inhibition of AChE activities

To evaluate AChE inhibitory activity of the synthesized carbamates 1a–5f and assess their potential for agricultural and medical applications, an AChE inhibition assay was performed using AChE from *Electrophorus electricus* (eeAChE). The assay was conducted according to Ellman's method, employing acetylthiocholine iodide as the substrate. In the presence of the chromogenic agent 5,5′-dithio-bis-(2-nitrobenzoic acid) (DTNB), enzymatic hydrolysis produces the yellow-colored 5-thio-2-nitrobenzoate anion, enabling the quantification of inhibition activity based on absorbance measurements.

The AChE inhibitory activities of compounds 1a–5f are summarized in Table 2. The results indicated that compounds 2a, 2c, 3a, 3c, 5a–5f exhibit strong inhibitory potency. The series-5 compounds demonstrated particularly excellent AChE inhibition, with 5a showing nanomolar-level activity.

**Table 2:** Inhibitory effects of compounds 1**a**–**5f**on EeAChE (IC_50_, µM or % inhibition at 20 µM)^a^

Compd.	AChE b	Compd.	AChE b
	(IC_50_, µM or % inhibition at 20 µM)		(IC_50_, µM or % inhibition at 20 µM)
1a	4.84 ± 0.97	3e	16.0 ± 0.8%
1b	15.7 ± 1.9%	3f	na c
1c	18.3 ± 2.5%	4a	0.96 ± 0.12
1d	na	4b	46.8 ± 6.4%
1e	na	4c	19.54 ± 0.90
1f	na	4d	43.4 ± 2.2%
2a	0.17 ± 0.02	4e	51.5 ± 12.2%
2b	8.2 ± 2.1%	4f	8.2 ± 0.5%
2c	3.56 ± 0.22	5a	0.0039 ± 0.0025
2d	44.1 ± 0.1%	5b	2.95 ± 0.58
2e	18.8 ± 5.4%	5c	0.57 ± 0.05
2f	na	5d	2.00 ± 0.50
3a	0.16 ± 0.03	5e	3.23 ± 0.08
3b	11.92 ± 0.98	5f	1.45 ± 0.25
3c	3.37 ± 0.72	Donepezil	0.032 ± 0.007
3d	44.5 ± 3.8%		

a Each IC_50_ value was calculated based on the average ± SEM of three separate trials.

b Acetylcholinesterase derived from electric eel.

c na, Inhibition of EeAChE and eqBuChE was not observed at 20 µM.

SAR analysis of carbamates series 1–5 showed that *N*-methyl analogs exhibited the most potent AChE inhibition, which generally decreased with larger *N*-alkyl groups—except for the cyclobutyl derivative, which displayed enhanced activity. This exception may be explained by the narrow, flat structure of the AChE active site gorge, allowing better binding of this conformation (Fig. S1). Optimal activity was also observed when the O-terminus bore an ortho-substituent of 3–5 atoms, often including heteroatoms.

As shown in Table 2, AChE activity varies significantly with *O*- and *N*-substituents. Notably, commercial methyl carbamates showed potent inhibition in both nematodes and mammals, guiding future efforts toward the discovery of nematode-selective AChE inhibitors.

### 3.6. Molecular docking study

**Fig. 3:**
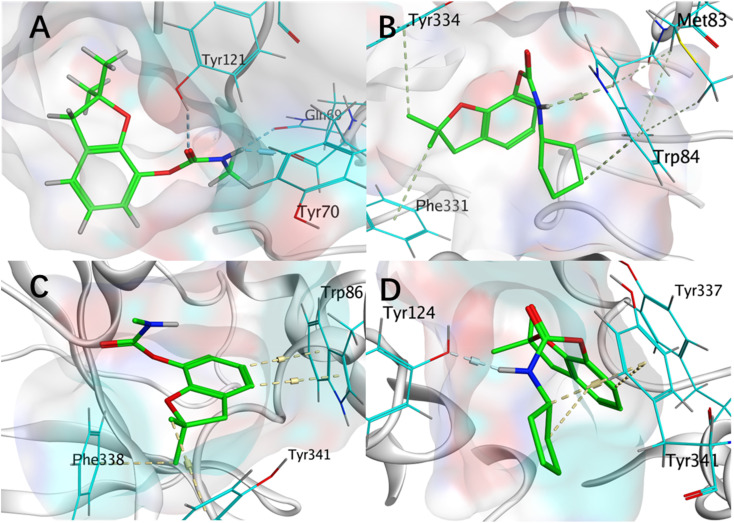
Molecular interactions of 5a and 5d with receptor AChE. 3D interaction diagrams of 5a (A) and 5d (B) bound to eeAChE (PDB: 6TT0); 3D interaction diagrams of 5a (C) and 5d (D) bound to hAChE (PDB: 7D9O). Ligands 5a and 5d are shown in light green. Hydrogen bonds and other key interactions are indicated by light blue and green dashed lines, respectively. Critical binding residues are highlighted in turquoise.

Molecular docking of potent inhibitor 5a (*N*-methyl) with eeAChE (6TT0) revealed key hydrogen bonds between its carbamate group and Tyr70, Tyr121, and Gln69, stabilizing a conformation favorable for carbamylation. In contrast, bulkier analog 5d formed only weak H–π interactions and encountered steric hindrance at the active site entrance, explaining its lower inhibitory activity (Fig. 3A and S2A). Docking with *h*AChE (7D9O) showed similar binding modes for both compounds: 5a engaged in H–π interactions with Trp86, Tyr341, and Phe338, while 5d retained Tyr341 binding and formed a hydrogen bond with Tyr124 but lost other key contacts (Fig. 3B and S2B). Despite this, 5d achieved a higher docking score than 5a.

Across the series, smaller *N*-alkyl groups (*e.g.*, methyl) facilitated active site entry, whereas larger substituents yielded more negative docking scores—a trend consistent with biochemical data (Table S1). These findings rationalize the SAR and suggest that scaffold optimization could reduce mammalian toxicity while retaining efficacy.

### 3.7. Cell viability assay

**Fig. 4:**
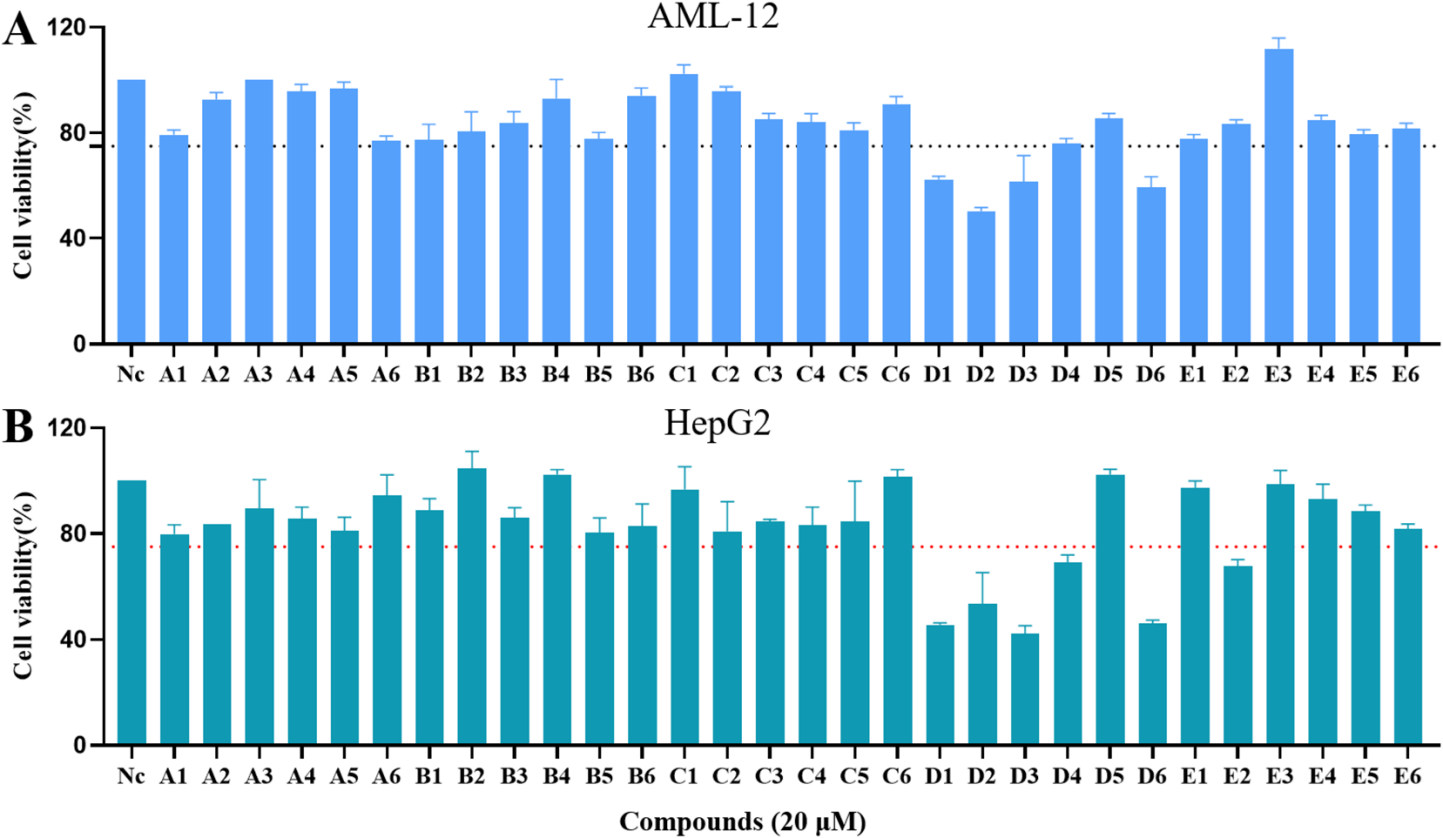
Effect of carbamate derivatives on the survival of AML-12 cells (A) and HepG2 cells (B).

The effect of carbamate derivatives on AML-12 and HepG2 cells was first evaluated in an *in vitro* assay. At concentrations below 20 µM, most carbamates had a negligible effect on cell viability (Fig. 4A and B). It was shown that most of the compounds studied had cell survival rates of more than 75%, with less liver damage, and that most of the compounds at the dose were suitable for subsequent biological studies. The toxicity of carbamates was closely related to the structure of phenols, which containing naphthalene exhibited a slight cytotoxicity.

## 4 Conclusion

In summary, we have established a mild, efficient, and environmentally benign synthetic route to carbamate derivatives using 3-substituted dioxazolones as practical isocyanate surrogates. The optimized method—employing MeCN as solvent and catalytic Cs_2_CO_3_ under mild heating—enables rapid, high-yielding carbamate formation without toxic phosgene or specialized equipment. The reaction demonstrates broad applicability, successfully affording a library of 30 carbamate analogues, including known pesticides such as propoxur and carbofuran. Biological assessment revealed strong AChE inhibitory activity for several compounds, with the series-5 showing particularly potent, nanomolar-level inhibition. SAR and molecular docking studies underscored the importance of an *N*-terminal methyl group and an *ortho*-substituted phenolic ring for optimal AChE binding and activity. This work not only offered a scalable and green synthetic strategy for carbamate chemicals but also provides a promising structural foundation for the development of selective AChE inhibitors with potential applications in AChE-inhibiting agent development.

## Author contributions

Yinxin Wu: conceptualization, data curation, formal analysis, investigation, methodology, software, visualization, writing – original draft; Xiaodan Liu: conceptualization, data curation, investigation, methodology, visualization, writing – original draft; Fangfang Zuo: formal analysis, investigation, methodology, validation, writing – original draft; Yulu Ding: conceptualization, data curation, investigation, software, visualization, writing – original draft; Jiasheng Kang: conceptualization, funding acquisition, project administration, resources, software, supervision; Jianping Wu: formal analysis, investigation, methodology, resoures, validation; Wenjian Tang: conceptualization, data curation, formal analysis, funding acquisition, investigation, methodology, software, supervision, validation, writing – review & editing.

## Conflicts of interest

The authors declare that they have no known competing financial interests or personal relationships that could have appeared to influence the work reported in this paper.

## Supplementary Material

RA-016-D6RA00983B-s001

## Data Availability

Data will be made available on request. Supplementary information (SI): the docking scores (kcal mol^−1^) of carbamate compounds with AChE (Table S1), 3D (Fig. S1) and 2D (Fig. S2) mode of interactions of 5a and 5d with receptor AChEs, general information of synthesis of carbamate compounds 1a–5f and their optimization of the reaction conditions, and the characterization data and their copies of NMR spectra and HRMS of 1a–5f. See DOI: https://doi.org/10.1039/d6ra00983b.
