# Supplementary material for: Mild and efficient synthesis of carbamates using dioxazolones as bench-stable isocyanate surrogates: application in AChE-inhibiting agent development
Source: RSC Adv. 2026 Mar 24;16(18):16220–8. doi: 10.1039/d6ra00983b (PMC13010858; doi:10.1039/d6ra00983b)
Supplement: RA-016-D6RA00983B-s001 [file RA-016-D6RA00983B-s001.pdf]

Supplementary Information (SI) for RSC Advances.
This journal is © The Royal Society of Chemistry 2026

Supporting Information

Mild and efficient synthesis of carbamates using dioxazolones as bench-stable isocyanate surrogates: Application in AChE-inhibiting agent development

Yinxin Wu,[a] Xiaodan Liu,[a] Fangfang Zuo,[a] Yulu Ding,[a] Jiasheng Kang,[b] Jianping Wu,[*b] Wenjian Tang,[*ab] Jing Zhang[*c]

[a] *School of Pharmacy, Anhui Medical University, Hefei 230032, China*

[b] *Anhui Province Key Laboratory of Green Manufacturing in Phosgene Industry, Anhui Guangxin Agrochemical Co., Ltd., Guangde 242200, China*

[c] *Anhui Province Key Laboratory of Occupational Health, Anhui No.2 Provincial People's Hospital, Hefei 230041, China*

Table of contents

1. **Table S1.** Docking scores (kcal/mol) of carbamate compounds with AChE...........S2
2. **Fig. S1.** Schematic diagram of the interaction (VDW) in the active pocket of human *h*AChE..........S3
3. **Fig. S2.** 2D mode of interactions of **5a** and **5d** with receptor AChEs.....................S4
4. General information of synthesis..........S5
5. Optimization of the reaction conditions..........S6
6. Synthesis and characterization data of **1a–5f**..........S12
7. The copies of NMR spectra and HRMS of **1a–5f**..........S26

Table S1. Docking scores (kcal/mol) of carbamate compounds with AChE (PDB: 6TT0, 7D9O).

Compd.	6TT0	7D9O
**1a**	–5.3129	–5.4369
**1b**	–5.8117	–6.0918
**1c**	–5.9608	–6.0454
**1d**	–6.1716	–6.3851
**1e**	–6.1507	–6.3418
**1f**	–6.0331	–6.1067
**2a**	–5.9676	–6.1189
**2b**	–6.4757	–6.6822
**2c**	–6.7661	–6.7778
**2d**	–7.1777	–7.0168
**2e**	–7.2480	–6.9662
**2f**	–6.8856	–6.6223
**3a**	–6.4029	–6.2284
**3b**	–6.6376	–6.3183
**3c**	–6.8289	–6.7875
**3d**	–7.1724	–7.0586
**3e**	–6.9189	–6.8828
**3f**	–6.8455	–6.4411
**4a**	–5.6083	–5.7529
**4b**	–6.1481	–6.3998
**4c**	–6.4141	–6.5733
**4d**	–6.6313	–6.6033
**4e**	–6.5182	–6.8393
**4f**	–6.4459	–6.5258
**5a**	–6.2165	–6.2958
**5b**	–6.6315	–6.4171

**5c**	–6.8136	–6.9055
**5d**	–7.1976	–6.9236
**5e**	–7.0987	–7.1120
**5f**	–7.0161	–6.5891

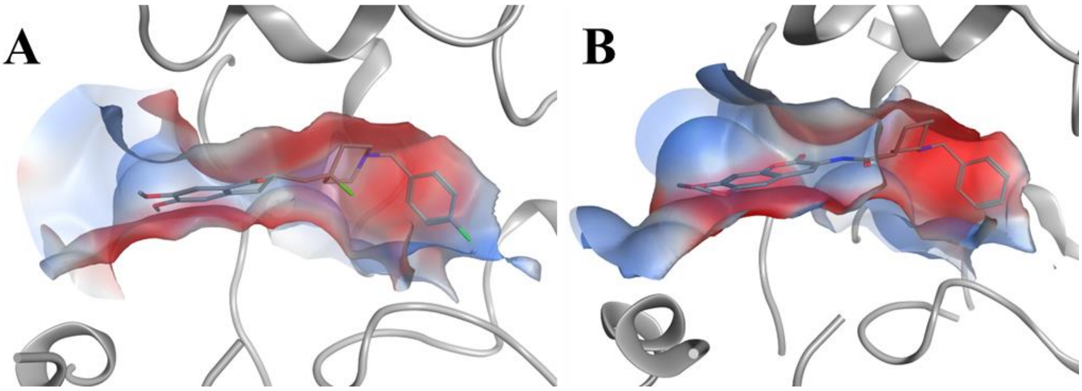

Fig. S1. Schematic diagram of the interaction (VDW) in the active pocket of human *h*AChE (**A**) (PDB: 7D9O, ligand: (2*R*)-2-[[4-fluoro-1-[(4-fluorophenyl)methyl]piperidin-4-yl]methyl]-5,6-dimethoxy-2,3-dihydroinden-1-one) and electric eel AChE (**B**) (PDB: 6TT0, ligand: (1*R*,3*S*)-*N*-(6,7-dimethoxy-2-oxochromen-3-yl)-3-[(phenylmethyl)amino]cyclohexane-1-carboxamide. Hydrophilic regions of the pocket are shown in blue, and hydrophobic regions in red.

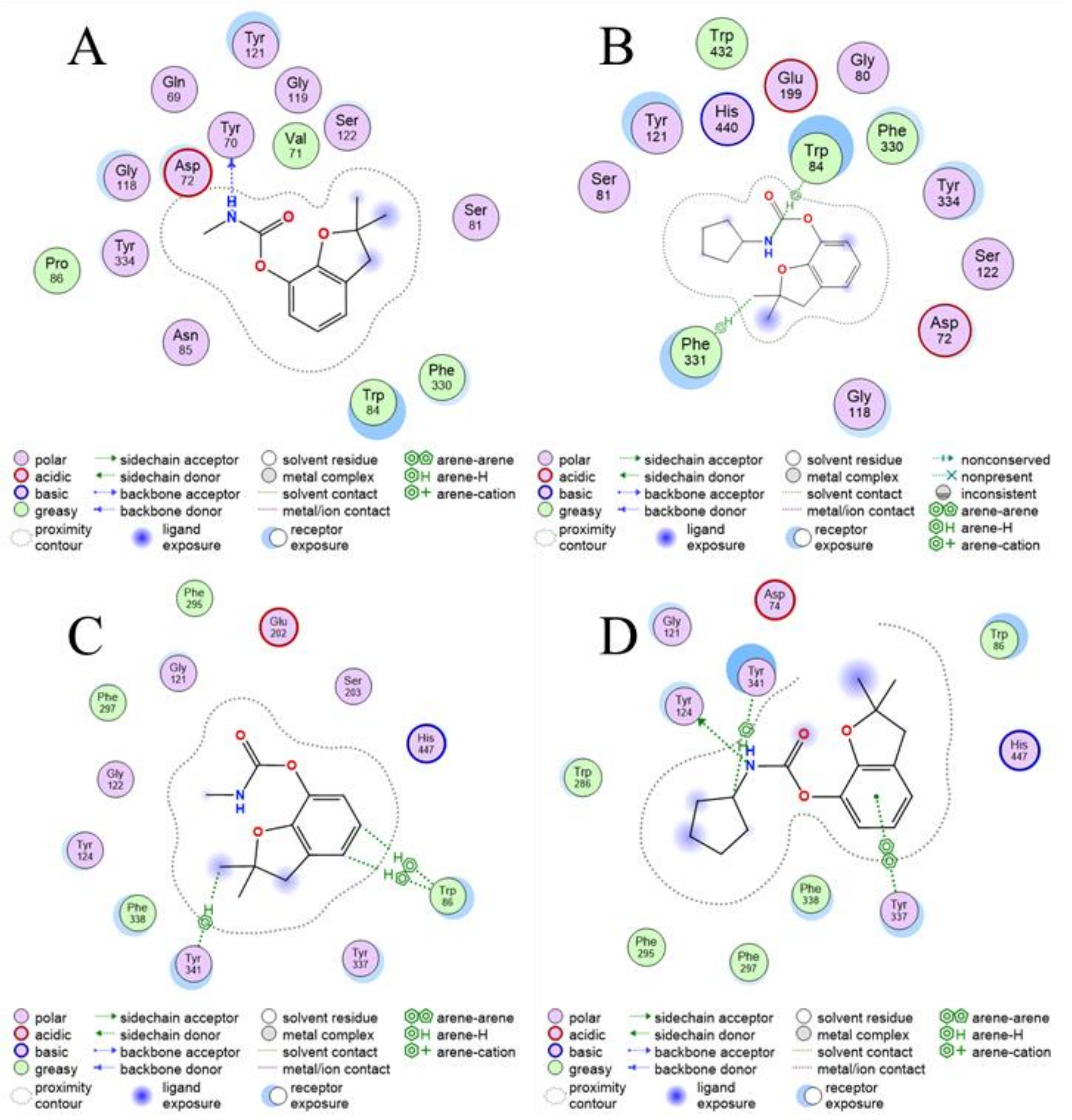

Fig. S2. 2D mode (**A**) of interactions of **5a** with receptor eeAChE (PDB: 6TT0) and 2D mode (**B**) of interactions of **5d** with receptor eeAChE (PDB: 6TT0); 2D mode (**C**) of interactions of **5a** with receptor AChE (PDB: 7D9O) and 2D mode (**D**) of interactions of **5d** with receptor AChE (PDB: 7D9O).

1. General information

All reactions were carried out under an air atmosphere unless otherwise specified. Oil bath was used for the heating reactions. NMR spectra were recorded in $CDCl_3$ / DMSO-d_6 on a 500 MHz (for ^{1}H/ for ^{13}C) or 400 MHz (for ^{1}H/ for ^{13}C) spectrometer. All chemical shifts are reported in ppm relative to TMS (0 ppm) as an internal standard. The following abbreviations were used to explain the multiplicities: *s* = singlet, *d* = doublet, *t* = triplet, *q* = quartet, *m* = multiplet. The coupling constants were reported in Hertz (Hz).

The reaction between 2,2-dimethyl-2,3-dihydrobenzofuran-7-ol (**5**) and 3-cyclopentyl-1,4,2-dioxazol-5-one (**D**) to form 2,2-dimethyl-2,3-dihydrobenzofuran-7-yl cyclopentylcarbamate (**5d**) was selected as the model for optimization. To monitor and quantify the yield under optimized conditions, high-performance liquid chromatography (HPLC) was preferred for the detection of both reactants and products. HPLC analysis was performed on a Shimadzu system equipped with a J&K RP-C18 column (5 μm, 4.6 × 150 mm). A pure, independently synthesized sample of **5d** was used as an external standard for quantification.

Unless otherwise specified, an acetonitrile (A)-water (B) gradient was applied as follows: 0-2 min, 60% A; 2-3 min, linear increase to 80% A; 3-7 min, maintained at 80% A; 7-8 min, increase to 90% A; 8-10 min, maintained at 90% A; 10-11 min, decrease to 60% A; and 11-14 min, re-equilibration at 60% A. The total run time was 20 min with a flow rate of 1.0 mL/min. The retention times and detection wavelength were: TR, **5d** = 6.520 min, **5** = 3.526 min at λ_{max} = 254.0 nm. All other reagents were obtained from commercial sources and used without further purification.

2. Optimization of the reaction conditions

Table S2. Screening of the solvent [a]

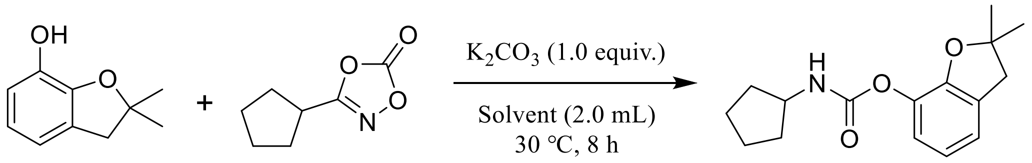

5 **D** **5d**

Entry	Solvent	Yield (**5d**, %) [b]
1	DCM	9
2	Ethyl acetate	6
3	MeOH	14
**4**	**MeCN**	**22**
5	THF	5
**6**	**Acetone**	**25**
7	Toluene	10
8	1,4-Dioxane	3
9	DMF	12
10	DMSO	5

[a] Dissolve 2,2-dimethyl-2,3-dihydrobenzofuran-7-ol (**5**, 32.8 mg, 0.2 mmol), 3-cyclopentyl-1,4,2-dioxazol-5-one (**D**, 31.1 mg, 0.2 mmol, 1.0 equiv.) in solvent (2.0 mL) by stirring with anhydrous potassium carbonate (27.6 mg, 1.0 equiv.) and react at 30 degrees Celsius for 8 h. [b] The yield was determined by high-performance liquid chromatography, using pure **5d** as an external standard.

Table S3. Screening of the base [a]

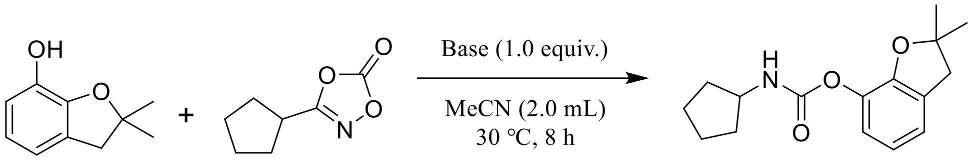

5 D 5d

Entry	Base	Yield (**5d**, %) [b]
1	KOAc	13
2	Et_3N	6
3	DIPEA	4
4	DBU	8
5	Na_2CO_3	15
6	K_2CO_3	21
**7**	**Cs_2CO_3**	**29**
8	NaOH	12

[a] Dissolve 2,2-dimethyl-2,3-dihydrobenzofuran-7-ol (**5**, 32.8 mg, 0.2 mmol), 3-cyclopentyl-1,4,2-dioxazol-5-one (**D**, 31.1 mg, 0.2 mmol, 1.0 equiv.) in MeCN (2.0 mL) by stirring with anhydrous base (27.6 mg, 1.0 equiv.) and react at 30 degrees Celsius for 8 h.

[b] The yield was determined by high-performance liquid chromatography, using pure **5d** as an external standard.

Table S4. Screening of the **D** equivalent [a]

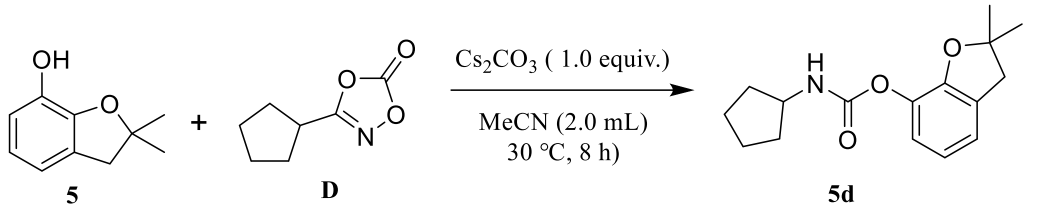

5 **D** **5d**

Entry	**D** (X equiv.)	Yield (**5d**, %) [b]
1	1	29
2	1.2	32
3	1.5	47
4	2	59
5	3	61
6	4	63

[a] Dissolve 2,2-dimethyl-2,3-dihydrobenzofuran-7-ol (**5**, 32.8 mg, 0.2 mmol), 3-cyclopentyl-1,4,2-dioxazol-5-one (**D**) in MeCN (2.0 mL) by stirring with anhydrous cesium carbonate (65.2 mg, 1.0 equiv.) and react at 30 degrees Celsius for 8 h.

[b] The yield was determined by high-performance liquid chromatography, using pure **5d** as an external standard.

Table S5. Screening of the effects of base equivalent and reaction time [a]

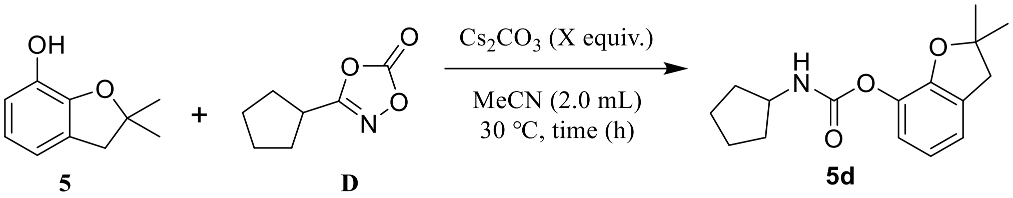

Entry	Cs_2CO_3 (X equiv.)	Yield (**5d**, 2h, %)	Yield (**5d**, 4h, %)	Yield (**5d**, 6h, %)	Yield (**5d**, 8h, %)	Yield (**5d**, 12h, %) [b]
1	0.1	23	35	45	51	54
2	0.2	26	37	46	53	55
3	0.5	34	46	53	58	57
4	1	47	55	58	58	53
5	2	57	55	52	49	44

[a] Dissolve 2,2-dimethyl-2,3-dihydrobenzofuran-7-ol (**5**, 32.8 mg, 0.2 mmol), 3-cyclopentyl-1,4,2-dioxazol-5-one (**D**, 62.2 mg, 0.4 mmol, 2.0 equiv.) in MeCN (2.0 mL) by stirring with anhydrous cesium carbonate and react at 40 degrees Celsius.

[b] The yield was determined by high-performance liquid chromatography at 2 h, 4 h, 6 h, 8 h, and 12 h, using pure **5d** as an external standard.

Table S6. Screening of the highest yield corresponding to different reaction temperatures [a]

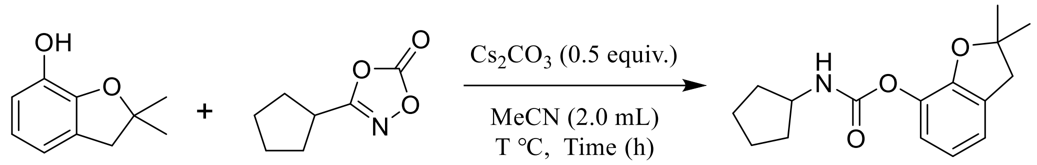

E **4** **E4**

Entry	Temperature (°C)	Yield (**5d**, 7 min, %)	Yield (**5d**, 20 min, %)	Yield (**5d**, 1h, %)	Yield (**5d**, 2h, %)	Yield (**5d**, 4h, %) [b]
1	40	\	\	\	\	60
2	50	\	\	\	67	
3	60	\	\	73	\	\
4	70	\	86	\	\	\
5	80	74	\	\	\	\

[a] A mixture of 2,2-dimethyl-2,3-dihydrobenzofuran-7-ol (**5**, 32.8 mg, 0.2 mmol), 3-cyclopentyl-1,4,2-dioxazol-5-one (**D**, 62.2 mg, 0.4 mmol, 2.0 equiv.), and cesium carbonate (32.6 mg, 0.1 mmol, 0.5 equiv.) was stirred and dissolved in acetonitrile (2.0 mL), and the reaction was conducted at 40, 50, 60, 70, and 80 °C, respectively.

The reaction mixture at 40 °C was sampled every 30 min over 3-5 h, and the yield was determined by high-performance liquid chromatography (HPLC). The yield reached its maximum at 4 h. For the 50 °C system, sampling was performed every 15 min within 1–3 h, with the highest yield observed at 2 h. At 60 C, samples were taken every 10 min during 0.5–1.5 h, and the maximum yield was achieved at 1 h. For the 70 °C condition, the mixture was sampled every 2 min between 15–30 min, and the yield peaked at 21 min. At 80 °C, sampling was carried out every 1 min within 5–15 min, with the highest yield attained at 8 min. Pure compound **5d** was used as an external standard for all HPLC analyses.

3. HPLC Chromatograms

SHIMADZU LabSolutions **Analysis Report**

<Sample message>

Sample	: 21min		
ID	: 21min		
Data Name	: 21min.lcd		
Method Name	: 60%MeCN to 80% MeCN to 60%MeCN.lcm		
Batch Name	: MeCN 1mmol 10ml 70℃.lcb		
Sample Number	: 1-5		
Injection volume	: 10 uL		
Acquisition Date	: 2025/7/18 16:34:39	Operator	: System Administrator
Modified Date	: 2025/7/18 16:54:46	Modified by	: System Administrator

<Chromatogram>

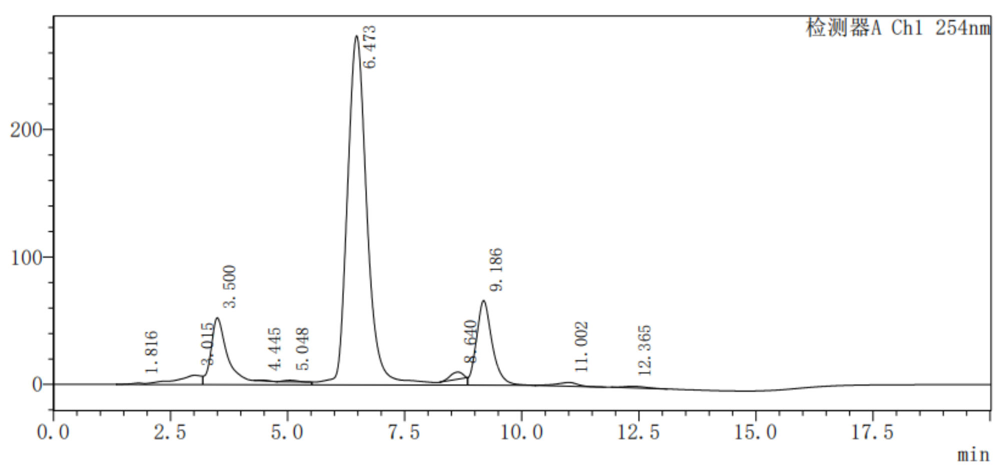

<峰表>

检测器A Ch1 254nm

peak	retention time	square	height	concentration	sign
1	1.816	20082	1104	0.000	
2	3.015	288129	7321	0.000	V
3	3.500	1468815	52442	0.000	SV
4	4.445	8292	561	0.000	T
5	5.048	23798	1210	0.000	T
6	6.473	8137939	273795	0.000	SV
7	8.640	106706	5611	0.000	T
8	9.186	1549977	66354	0.000	V
9	11.002	89688	2943	0.000	
10	12.365	39793	1186	0.000	
总计		11733219	412528		

HPLC Chromatograms

In the HPLC chromatogram, the peak corresponding to the starting material, 2,2-dimethyl-2,3-dihydrobenzofuran-7-ol (**5**), appeared at a retention time (Rt) of 3.500 min. In contrast, the target product, 2,2-dimethyl-2,3-dihydrobenzofuran-7-yl cyclopentylcarbamate (**5d**), exhibited a sharp and well-defined peak at R_t = 6.473 min, with good baseline separation between the two compounds.

4. Experimental procedures

4.1 Synthesis of 3-methyl-1,4,2-dioxazol-5-one (**A**)

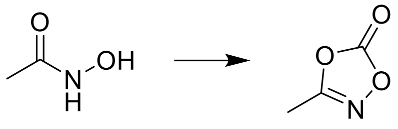

I **A**

Triethylamine (1.5 mL) was added dropwise to a mixture of acetohydroxamic acid (**I**, 750 mg, 10 mmol) and triphosgene (1.2 g, 4 mmol) in dichloromethane at 0 °C (ice bath). The reaction mixture was stirred for 30 min. The mixture was then washed with 1N HCl (50 mL) and saturated NaCl solution (2 × 40 mL). The organic phase was collected, dried over anhydrous Na_2SO_4, filtered, and concentrated under reduced pressure to afford pure 3-methyl-1,4,2-dioxazol-5-one (**A**).

4.2 Synthesis of 3-cyclopropyl-1,4,2-dioxazol-5-one (**B**)

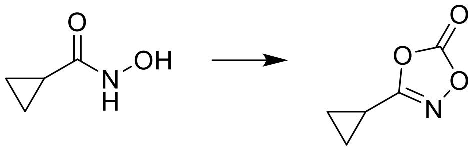

II **B**

Triethylamine (1.5 mL) was added dropwise to a mixture of *N*-hydroxycyclopropanecarboxamide (**II**, 1.01 g, 10 mmol) and triphosgene (1.2 g, 4 mmol) in dichloromethane at 0 °C (ice bath). The reaction mixture was stirred for 30 min. The mixture was then washed with 1N HCl (50 mL) and saturated NaCl solution (2 × 40 mL). The organic phase was collected, dried over anhydrous Na_2SO_4, filtered, and concentrated under reduced pressure to afford pure 3-cyclopropyl-1,4,2-dioxazol-5-one (**B**).

4.3 Synthesis of 3-cyclobutyl-1,4,2-dioxazol-5-one (**C**)

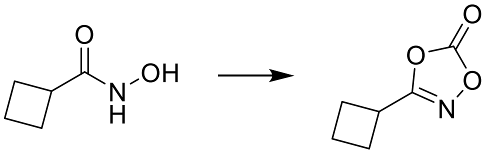

III **C**

Triethylamine (1.5 mL) was added dropwise to a mixture of *N*-hydroxycyclobutanecarboxamide (**III**, 1.15 g, 10 mmol) and triphosgene (1.2 g, 4 mmol) in dichloromethane at 0 °C (ice bath). The reaction mixture was stirred for 30 min. The mixture was then washed with 1N HCl (50 mL) and saturated NaCl solution (2 × 40 mL). The organic phase was collected, dried over anhydrous Na_2SO_4, filtered, and concentrated under reduced pressure to afford pure 3-cyclobutyl-1,4,2-dioxazol-5-one (**C**).

4.4 Synthesis of 3-cyclopentyl-1,4,2-dioxazol-5-one (**D**)

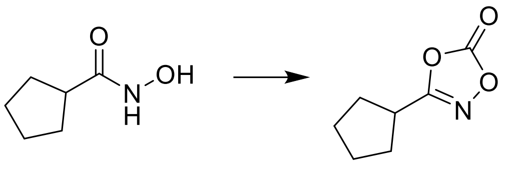

IV **D**

Triethylamine (1.5 mL) was added dropwise to a mixture of *N*-hydroxycyclopentanecarboxamide (**IV**, 1.29 g, 10 mmol) and triphosgene (1.2 g, 4 mmol) in dichloromethane at 0 °C (ice bath). The reaction mixture was stirred for 30 min. The mixture was then washed with 1N HCl (50 mL) and saturated NaCl solution (2 × 40 mL). The organic phase was collected, dried over anhydrous Na_2SO_4, filtered, and concentrated under reduced pressure to afford pure 3-cyclopentyl-1,4,2-dioxazol-5-one (**D**).

4.5 Synthesis of 3-cyclohexyl-1,4,2-dioxazol-5-one (**E**)

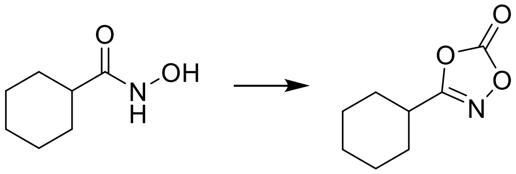

V **E**

Triethylamine (1.5 mL) was added dropwise to a mixture of *N*-hydroxycyclohexanecarboxamide (**V**, 1.43 g, 10 mmol) and triphosgene (1.2 g, 4 mmol) in dichloromethane at 0 °C (ice bath). The reaction mixture was stirred for 30 min. The

mixture was then washed with 1N HCl (50 mL) and saturated NaCl solution (2 × 40 mL). The organic phase was collected, dried over anhydrous Na_2SO_4, filtered, and concentrated under reduced pressure to afford pure 3-cyclohexyl-1,4,2-dioxazol-5-one (**E**).

4.6 Synthesis of 3-phenyl-1,4,2-dioxazol-5-one (**F**)

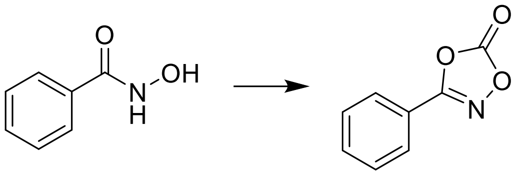

VI **F**

Triethylamine (1.5 mL) was added dropwise to a mixture of *N*-hydroxybenzamide (**VI**, 1.37 g, 10 mmol) and triphosgene (1.2 g, 4 mmol) in dichloromethane at 0 °C (ice bath). The reaction mixture was stirred for 30 min. The mixture was then washed with 1N HCl (50 mL) and saturated NaCl solution (2 × 40 mL). The organic phase was collected, dried over anhydrous Na_2SO_4, filtered, and concentrated under reduced pressure to afford pure 3-phenyl-1,4,2-dioxazol-5-one (**F**).

4.7 Synthesis of m -tolyl-substituted carbamates (**1a–1f**)

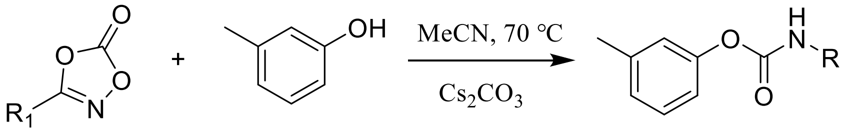

A-F **1** **1a–1f**

A mixture of m -cresol (**1**, 216 mg, 2 mmol), 3-substituted-1,4,2-dioxazol-5-one (**A-F**, 6 mmol), and cesium carbonate (325 mg, 1 mmol) in acetonitrile was stirred and heated at 70 °C for 21 min. After completion of the reaction, the mixture was extracted with ethyl acetate (30 mL) and washed with saturated sodium chloride solution (2 × 30 mL). The organic phase was dried over anhydrous sodium sulfate, filtered, and concentrated under reduced pressure. The crude product was purified by column chromatography using a mixture of petroleum ether and ethyl acetate (petroleum ether / ethyl acetate = 15: 1) as the eluent. The resulting product was recrystallized from a mixture of petroleum ether and ethyl acetate (30: 1) to afford pure solid products (**1a–**

5f).

4.8 Synthesis of 2 - (sec-butyl) phenyl *N*-substituted carbamates (**2a–2f**)

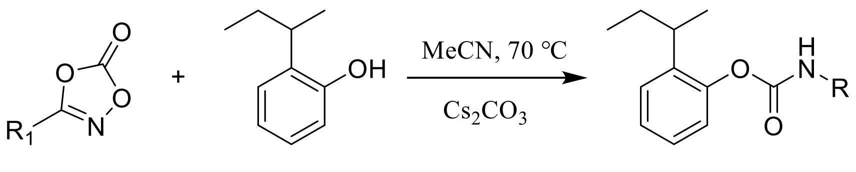

A-F **2** **2a–2f**

A mixture of 2-(sec-butyl) phenol (**2**, 300 mg, 2 mmol), 3-substituted-1,4,2-dioxazol -5-one (**A-F**, 4 mmol), and cesium carbonate (325 mg, 1 mmol) in acetonitrile was stirred and heated at 70 °C for 21 min. After completion of the reaction, the mixture was extracted with ethyl acetate (30 mL) and washed with saturated sodium chloride solution (2 × 30 mL). The organic phase was dried over anhydrous sodium sulfate, filtered, and concentrated under reduced pressure. The crude product was purified by column chromatography using a mixture of petroleum ether and ethyl acetate (petroleum ether / ethyl acetate =10: 1) as the eluent to give the target compounds (**2a–2f**).

4.9 Synthesis of 2-isopropoxyphenyl *N*-substituted carbamates (**3a–3f**)

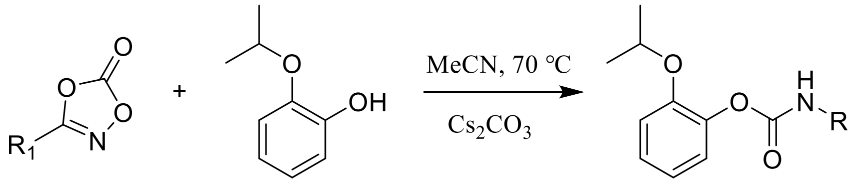

A-F **3** **3a–3f**

A mixture of 2-isopropoxyphenol (**3**, 304 mg, 2 mmol), 3-substituted-1,4,2-dioxazol-5-one (**A-F**, 4 mmol), and cesium carbonate (325 mg, 1 mmol) in acetonitrile was stirred and heated at 70 °C for 21 min. After completion of the reaction, the mixture was extracted with ethyl acetate (30 mL) and washed with saturated sodium chloride solution (2 × 30 mL). The organic phase was dried over anhydrous sodium sulfate, filtered, and concentrated under reduced pressure. The crude product was purified by column chromatography using a mixture of petroleum ether and ethyl acetate

(petroleum ether / ethyl acetate = 10: 1) as the eluent to give the target compounds (**3a**–**3f**).

4.10 Synthesis of 1-naphthyl *N*-substituted carbamates (**4a**–**4f**)

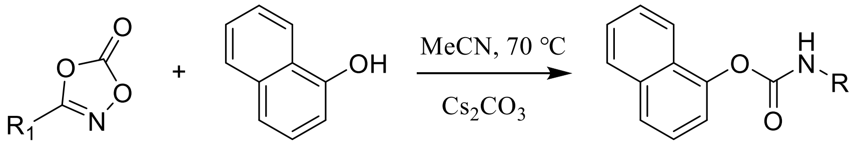

A-F **4** **4a–4f**

A mixture of 1-naphthol (**4**, 288 mg, 2 mmol), 3-substituted-1,4,2-dioxazol-5-one (**A-F**, 4 mmol), and cesium carbonate (325 mg, 1 mmol) in acetonitrile was stirred and heated at 70 °C for 21 min. After completion of the reaction, the mixture was extracted with ethyl acetate (30 mL) and washed with saturated sodium chloride solution (2 × 30 mL). The organic phase was dried over anhydrous sodium sulfate, filtered, and concentrated under reduced pressure. The crude product was purified by column chromatography using a mixture of petroleum ether and ethyl acetate (petroleum ether / ethyl acetate = 10: 1) as the eluent to give the target compounds (**4a**–**4f**).

4.11 Synthesis of 2,2-dimethyl-2,3-dihydrobenzofuran-7-yl *N*-substituted carbamates (**5a**–**5f**)

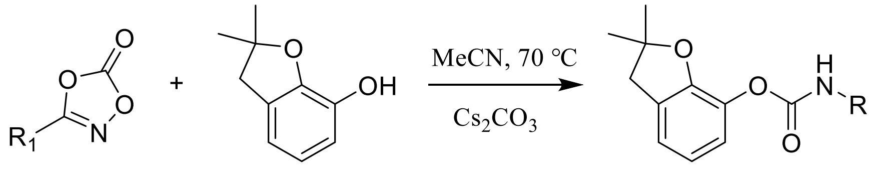

A-F **5** **5a–5f**

A mixture of 2,2-dimethyl-2,3-dihydrobenzofuran-7-ol (**5**, 328 mg, 2.0 mmol), 3-substituted-1,4,2-dioxazol-5-one (**A-F**, 4 mmol), and cesium carbonate (325 mg, 1 mmol) in acetonitrile was stirred and heated at 70 °C for 21 min. After completion of the reaction, the mixture was extracted with ethyl acetate (30 mL) and washed with saturated sodium chloride solution (2 × 30 mL). The organic phase was dried over anhydrous sodium sulfate, filtered, and concentrated under reduced pressure. The crude

product was purified by column chromatography using a mixture of petroleum ether and ethyl acetate (petroleum ether / ethyl acetate = 10: 1) as the eluent to give the target compounds (**5a–5f**).

5. Characterization

m-tolyl methylcarbamate (**1a**). White solid, 238 mg, 72 % yield. $C_9H_{11}NO_2$; M W: 165.19. ^{1}H NMR (500 MHz, $CDCl_3$) δ 7.23 (dd, J = 15.2, 7.4 Hz, 1H), 7.00 (d, J = 7.6 Hz, 1H), 6.97 – 6.86 (m, 2H), 5.02 (s, 1H), 2.86 (d, J = 4.9 Hz, 3H, CH_3), 2.34 (s, 3H, CH_3). ^{13}C NMR (126 MHz, $CDCl_3$) δ 155.61 (C=O), 151.18, 139.60, 129.18, 126.29, 122.44, 118.73, 27.86, 21.48; TOF-HRMS (ESI): *m/z* $[M + Na]^+$ calcd for $[C_9H_{11}NO_2Na]^+$: 188.0682; found: 188.0367.

m-tolyl cyclopropylcarbamate (**1b**). White solid, 288 mg, 75 % yield. $C_{11}H_{13}NO_2$; M W: 191.23. ^{1}H NMR (500 MHz, $CDCl_3$) δ 7.23 (dd, J = 15.9, 8.4 Hz, 1H), 7.05 – 6.86 (m, 3H), 5.26 (s, 1H), 2.67 (s, 1H), 2.34 (s, 3H, CH_3), 0.76 (q, J = 6.8 Hz, 2H), 0.67 – 0.55 (m, 2H). ^{13}C NMR (126 MHz, $CDCl_3$) δ 155.50 (C=O), 151.05, 139.58, 129.16, 126.28, 122.41, 118.71, 23.41, 21.48, 6.97 (2C). TOF-HRMS (ESI): *m/z* $[M + Na]^+$ calcd for $[C_{11}H_{13}NO_2Na]^+$: 214.0838; found: 188.0367.

m-tolyl cyclobutylcarbamate (**1c**). White solid. 284 mg, 69% yield. $C_{12}H_{15}NO_2$; M W: 205.26. ^{1}H NMR (400 MHz, $CDCl_3$) δ 7.23 (t, J = 7.8 Hz, 1H), 7.00 (d, J = 7.7 Hz, 1H), 6.94 (s, 1H), 6.91 (d, J = 8.7 Hz, 1H), 5.26 (d, J = 8.3 Hz, 1H), 4.22 (h, J = 8.3 Hz, 1H), 2.42 – 2.35 (m, 2H), 2.35 – 2.32 (m, 3H, CH_3), 1.98 – 1.87 (m, 2H), 1.70 (dd, J = 11.0, 6.6 Hz, 2H). ^{13}C NMR (101 MHz, $CDCl_3$) δ 153.68 (C=O), 151.00, 139.47, 129.07, 126.17, 122.39, 118.69, 46.39, 31.37 (2C), 21.40, 14.86. TOF-HRMS (ESI): *m/z* $[M + Na]^+$ calcd for $[C_{12}H_{15}NO_2Na]^+$: 228.0995; found: 228.0998.

m-tolyl cyclopentylcarbamate (**1d**). White solid, 340 mg, 78% yield. $C_{13}H_{17}NO_2$; M W: 219.28. ^{1}H NMR (400 MHz, $CDCl_3$) δ 7.23 (t, J = 7.8 Hz, 1H), 7.00 (d, J = 7.8 Hz,

1H), 6.95 (s, 1H), 6.92 (d, *J* = 8.3 Hz, 1H), 5.01 (d, *J* = 7.5 Hz, 1H), 4.05 (h, *J* = 7.2 Hz, 1H), 2.35 (s, 3H, CH_3), 2.01 (dq, *J* = 12.5, 6.4 Hz, 2H), 1.81 – 1.68 (m, 2H),1.61 (td, *J* = 7.5, 3.9 Hz, 2H), 1.48 (dq, *J* = 13.3, 6.6 Hz, 2H). ^{13}C NMR (101 MHz, $CDCl_3$) δ 154.28 (C=O), 151.09, 139.47, 129.07, 126.10, 122.40, 118.70, 53.07, 33.25 (2C), 23.66 (2C), 21.42. TOF-HRMS (ESI): *m/z* $[M + Na]^+$ calcd for $[C_{13}H_{17}NO_2Na]^+$: 242.1151; found: 242.0836.

m-tolyl cyclohexylcarbamate (**1e**). White solid. 372 mg, 80% yield. $C_{14}H_{19}NO_2$; M W: 233.31. ^{1}H NMR (400 MHz, $CDCl_3$) δ 7.23 (t, *J* = 7.8 Hz, 1H), 7.00 (d, *J* = 7.8 Hz, 1H), 6.95 (s, 1H), 6.92 (d, *J* = 7.8 Hz, 1H), 5.21 – 4.81 (m, 1H), 3.62 – 3.50 (m, 1H), 2.35 (s, 3H, CH_3), 2.00 (dd, *J* = 12.4, 4.3 Hz, 2H), 1.80 – 1.71 (m, 2H), 1.71 – 1.45 (m, 2H), 1.42 – 1.31 (m, 2H), 1.25 – 1.17 (m, 2H). ^{13}C NMR (101 MHz, $CDCl_3$) δ 153.93 (C=O), 151.12, 139.45, 129.05, 126.06, 122.38, 118.68, 50.19, 33.36, 25.56 (2C), 24.87 (2C), 21.42. TOF-HRMS (ESI): *m/z* $[M + H]^+$ calcd for $[C_{14}H_{20}NO_2]^+$: 234.1489; found: 234.1489.

m-tolyl phenylcarbamate (**1f**). White solid. 295 mg, 65% yield. $C_{14}H_{13}NO_2$; M W: 227.26. ^{1}H NMR (500 MHz, $CDCl_3$) δ 7.42 (d, *J* = 7.8 Hz, 2H), 7.35 – 7.28 (m, 2H), 7.28 – 7.22 (m, 1H), 7.09 (t, *J* = 7.4 Hz, 1H), 7.06 – 6.94 (m, 4H), 2.35 (s, 3H, CH_3). ^{13}C NMR (126 MHz, $CDCl_3$) δ 151.99 (C=O), 150.66, 139.80, 137.62, 129.31 (3C), 126.73, 124.02, 122.48 (2C), 118.87, 118.79, 21.51. TOF-HRMS (ESI): *m/z* $[M + Na]^+$ calcd for $[C_{14}H_{13}NO_2Na]^+$: 250.0838; found: 250.0831.

2-(sec-butyl)phenyl methylcarbamate (**2a**). Colorless oil. 364 mg, 88% yield. $C_{12}H_{17}NO_2$; M W: 207.27. ^{1}H NMR (400 MHz, $CDCl_3$) δ 7.24 (dd, J = 5.8, 3.6 Hz, 1H), 7.19 (t, *J* = 3.7 Hz, 1H), 7.17 (t, J = 3.7 Hz, 1H), 7.06 (d, J = 9.4 Hz, 1H), 5.00 (m, 1H), 2.90 (d, J = 4.9 Hz, 3H, CH_3), 2.89 – 2.82 (m, 1H), 1.62 – 1.53 (m, 2H), 1.20 (d, J = 6.9 Hz, 3H, CH_3), 0.83 (t, J = 7.4 Hz, 3H, CH_3). TOF-HRMS (ESI): *m/z* $[M + H]^+$ calcd for $[C_{12}H_{18}NO_2]^+$: 208.1332; found: 208.1332.

2-(sec-butyl)phenyl cyclopropylcarbamate (**2b**). White solid. 414mg, 89% yield. $C_{14}H_{19}NO_2$; M W: 233.31. ^{1}H NMR (400 MHz, $CDCl_3$) δ 7.24 (d, J = 6.7 Hz, 1H), 7.19 (s, 1H), 7.17 (s, 1H), 7.07 (s, 1H), 5.18 (d, J = 77.0 Hz, 1H), 2.94 – 2.79 (m, 1H), 2.70 (s, 1H), 1.66 – 1.47 (m, 2H), 1.20 (d, J = 7.0 Hz, 3H, CH_3), 0.83 (t, J = 7.6 Hz, 3H, CH_3), 0.78 (dt, J = 6.9, 3.3 Hz, 2H), 0.63 (d, J = 7.6 Hz, 2H). ^{13}C NMR (101 MHz, $CDCl_3$) δ 155.49 (C=O), 148.67, 139.59, 127.16, 126.54, 125.92, 122.65, 34.38, 30.28, 23.41, 20.93, 12.34, 7.04 (2C). TOF-HRMS (ESI): *m/z* $[M + H]^+$ calcd for $[C_{14}H_{20}NO_2]^+$: 234.1489; found: 234.1457.

2-(sec-butyl)phenyl cyclobutylcarbamate (**2c**). White solid. 386mg, 83% yield. $C_{15}H_{21}NO_2$; M W: 247.34. ^{1}H NMR (400 MHz, $CDCl_3$) δ 7.24 (t, J = 4.7 Hz, 1H), 7.18 (s, 1H), 7.18 – 7.13 (m, 1H), 7.11 – 6.96 (m, 1H), 5.22 (d, J = 8.4 Hz, 1H), 4.23 (h, J = 8.2 Hz, 1H), 2.86 (h, J = 7.0 Hz, 1H), 2.44 – 2.33 (m, 2H), 1.94 (dt, J = 20.5, 9.8 Hz, 2H), 1.70 (q, J = 9.4 Hz, 2H), 1.58 (dq, J = 16.4, 6.9 Hz, 2H), 1.20 (d, J = 6.9 Hz, 3H, CH_3), 0.83 (t, J = 7.3 Hz, 3H, CH_3). ^{13}C NMR (101 MHz, $CDCl_3$) δ 153.72 (C=O), 148.71, 139.65, 127.14, 126.51, 125.85, 122.68, 46.50, 34.32, 31.50, 31.43, 30.30, 20.95, 14.86, 12.34. TOF-HRMS (ESI): *m/z* $[M + H]^+$ calcd for $[C_{15}H_{22}NO_2]^+$: 248.1645; found: 248.1607.

2-(sec-butyl)phenyl cyclopentylcarbamate (**2d**). White solid. 480mg, 92% yield. $C_{16}H_{23}NO_2$; M W: 261.37. ^{1}H NMR (400 MHz, $CDCl_3$) δ 7.27 – 7.22 (m, 1H), 7.19 (d, J = 4.3 Hz, 1H), 7.17 (d, J = 5.1 Hz, 1H), 7.09 – 7.04 (m, 1H), 5.02 (d, J = 7.7 Hz, 1H), 4.06 (h, J = 6.9 Hz, 1H), 2.86 (h, J = 7.1 Hz, 1H), 2.02 (dq, J = 12.8, 6.5 Hz, 2H), 1.75 – 1.68 (m, 2H), 1.62 (dd, J = 10.3, 4.9 Hz, 2H), 1.56 (q, J = 6.3 Hz, 2H), 1.49 (dt, J = 12.6, 6.2 Hz, 2H), 1.20 (d, J = 7.0 Hz, 3H, CH_3), 0.83 (t, J = 7.4 Hz, 3H, CH_3). ^{13}C NMR (101 MHz, $CDCl_3$) δ 154.38 (C=O), 148.78, 139.69, 127.11, 126.50, 125.80, 122.74, 53.12, 34.36, 33.93, 33.30, 33.26, 30.31, 23.66, 20.93, 12.36. TOF-HRMS (ESI): *m/z* $[M + H]^+$ calcd for $[C_{16}H_{24}NO_2]^+$: 262.1802; found: 262.1766.

2-(sec-butyl)phenyl cyclohexylcarbamate (**2e**). White solid. 471mg, 86% yield. $C_{17}H_{25}NO_2$; M W: 275.39. ^{1}H NMR (400 MHz, $CDCl_3$) δ 7.33 – 7.26 (m, 1H), 7.22 (s, 1H), 7.21 – 7.17 (m, 1H), 7.12 – 7.07 (m, 1H), 5.02 (d, J = 8.2 Hz, 1H), 3.60 (td, J = 13.5, 8.1 Hz, 1H), 2.90 (h, J = 7.0 Hz, 1H), 2.04 (dd, J = 12.4, 4.3 Hz, 2H), 1.81 – 1.73 (m, 2H), 1.72 – 1.63 (m, 2H), 1.58 (dt, J = 13.7, 6.9 Hz, 2H), 1.38 (t, J = 12.5 Hz, 2H), 1.33 – 1.25 (m, 2H), 1.25 – 1.22 (m, 3H, CH_3), 0.87 (t, J = 7.5 Hz, 3H, CH_3). ^{13}C NMR (101 MHz, $CDCl_3$) δ 154.05 (C=O), 148.78, 139.70, 127.10, 126.47, 125.77, 122.75, 50.20, 34.38, 33.37, 30.30, 25.55 (2C), 24.88 (2C), 20.90, 12.35. TOF-HRMS (ESI): *m/z* $[M + H]^+$ calcd for $[C_{17}H_{26}NO_2]^+$: 276.1958; found: 276.1910.

2-(sec-butyl)phenyl phenylcarbamate (**2f**). White solid. 436mg, 82% yield. $C_{17}H_{19}NO_2$; M W: 269.34. ^{1}H NMR (400 MHz, $CDCl_3$) δ 7.47 (d, J = 8.0 Hz, 2H), 7.38 – 7.31 (m, 2H), 7.32 – 7.27 (m, 1H), 7.26 – 7.19 (m, 2H), 7.18 – 7.11 (m, 2H), 7.11 (d, 1H), 2.93 (h, J = 7.1 Hz, 1H), 1.61 (ddt, J = 16.8, 9.4, 4.2 Hz, 2H), 1.23 (d, J = 6.9 Hz, 3H, CH_3), 0.85 (t, J = 7.4 Hz, 3H, CH_3). ^{13}C NMR (101 MHz, $CDCl_3$) δ 151.88 (C=O), 148.26, 139.73, 137.63, 129.27 (2C), 127.32, 126.68, 126.36, 123.90, 122.66, 118.68 (2C), 34.36, 30.31, 21.09, 12.34. TOF-HRMS (ESI): *m/z* $[M + H]^+$ calcd for $[C_{17}H_{20}NO_2]^+$: 270.1489; found: 270.1472.

2-isopropoxyphenyl methylcarbamate (**3a**). White solid. 322 mg, 77% yield. $C_{11}H_{15}NO_3$; M W: 209.25. ^{1}H NMR (400 MHz, $CDCl_3$) δ 7.16 – 7.11 (m, 1H), 7.08 (dd, J = 8.0, 1.7 Hz, 1H), 6.96 (dd, J = 8.2, 1.5 Hz, 1H), 6.91 (td, J = 7.7, 1.5 Hz, 1H), 5.05 (s, 1H), 4.51 (hept, J = 6.1 Hz, 1H), 2.88 (d, J = 4.9 Hz, 3H, CH_3), 1.33 (d, J = 6.0 Hz, 6H, 2CH_3). TOF-HRMS (ESI): *m/z* $[M + Na]^+$ calcd for $[C_{11}H_{15}NO_3Na]^+$: 232.0944; found: 232.0948.

2-isopropoxyphenyl cyclopropylcarbamate (**3b**). White solid. 383mg, 81% yield. $C_{13}H_{17}NO_3$; M W: 235.28. ^{1}H NMR (500 MHz, $CDCl_3$) δ 7.17 – 7.11 (m, 1H), 7.08 (d,

J = 8.3 Hz, 1H), 6.95 (dd, J = 8.2, 1.4 Hz, 1H), 6.90 (t, J = 7.7 Hz, 1H), 5.31 (d, J = 19.9 Hz, 1H), 4.51 (p, J = 4.9 Hz, 1H), 2.68 (s, 1H), 1.33 (d, J = 6.1 Hz, 6H, 2CH_3), 0.80 – 0.73 (m, 2H), 0.66 (dd, J = 54.1, 5.2 Hz, 2H). ^{13}C NMR (126 MHz, $CDCl_3$) δ 155.10 (C=O), 150.15, 141.29, 126.35, 123.50, 120.82, 115.50, 71.50, 23.34, 22.25 (2C), 6.97 (2C). TOF-HRMS (ESI): *m/z* [M -C_3H_6+ H]$^+$ calcd for [$C_{10}H_{11}NO_3$]$^+$: 194.0812; found: 194.0816.

2-isopropoxyphenyl cyclobutylcarbamate (**3c**). White solid. 368mg, 74% yield. $C_{14}H_{19}NO_3$; M W: 249.31. ^{1}H NMR (500 MHz, $CDCl_3$) δ 7.15 – 7.10 (m, 1H), 7.08 (d, J = 7.9 Hz, 1H), 6.95 (dd, J = 8.2, 1.5 Hz, 1H), 6.94 – 6.87 (m, 1H), 5.26 (d, J = 8.0 Hz, 1H), 4.50 (hept, J = 6.1 Hz, 1H), 4.21 (h, J = 8.3 Hz, 1H), 2.36 (q, J = 7.7 Hz, 2H), 1.99 – 1.88 (m, 2H), 1.75 – 1.65 (m, 2H), 1.32 (d, J = 6.1 Hz, 6H, 2CH_3). ^{13}C NMR (126 MHz, $CDCl_3$) δ 153.37 (C=O), 150.23, 141.38, 126.30, 123.53, 120.88, 115.62, 71.63, 46.52, 31.37 (2C), 22.26 (2C), 14.89. TOF-HRMS (ESI): *m/z* [M + H]$^+$ calcd for [$C_{14}H_{20}NO_3$]$^+$: 250.1438; found: 250.1440.

2-isopropoxyphenyl cyclopentylcarbamate (**3d**). White solid. 447mg, 85% yield. $C_{15}H_{21}NO_3$; M W: 263.34. ^{1}H NMR (600 MHz, $CDCl_3$) δ 7.17 – 7.04 (m, 2H), 6.99 – 6.93 (m, 1H), 6.90 (t, J = 7.7 Hz, 1H), 4.04 – 5.05 (d, 1H), 4.51 (p, J = 6.0 Hz, 1H), 4.03 - 4.06 (q, 1H), 2.00 (dt, J = 12.5, 6.2 Hz, 2H), 1.76 – 1.44 (m, 6H, 3CH_2), 1.33 (d, J = 6.1 Hz, 6H, 2CH_3). ^{13}C NMR (151 MHz, $CDCl_3$) δ 154.03 (C=O), 150.33, 141.56, 126.31, 123.64, 120.94, 115.66, 71.63, 53.20, 33.29 (2C), 23.69 (2C), 22.33 (2C). TOF-HRMS (ESI): *m/z* [M + H]$^+$ calcd for [$C_{15}H_{22}NO_3$]$^+$: 264.1594; found: 264.1597.

2-isopropoxyphenyl cyclohexylcarbamate (**3e**). White solid. 428mg, 77% yield. $C_{16}H_{23}NO_3$; M W: 277.36. ^{1}H NMR (600 MHz, $CDCl_3$) δ 7.18 – 7.04 (m, 2H), 6.98 – 6.86 (m, 2H), 4.51 (p, J = 6.0 Hz, 1H), 2.00 (d, J = 9.9 Hz, 1H), 1.77 – 1.67 (m, 1H), 1.61 (dt, J = 12.5, 3.6 Hz, 2H), 1.47 – 1.29 (m, 3H), 1.35 – 1.38 (dt, 2H), 1.32 – 1.33 (d, 6H, 2CH_3), 1.26 – 1.10 (m, 3H). ^{13}C NMR (151 MHz, $CDCl_3$) δ 153.70 (C=O),

150.37, 141.60, 126.31, 123.67, 120.97, 115.73, 71.64, 50.20, 33.43, 25.69 (2C), 24.92 (2C), 22.34 (2C). TOF-HRMS (ESI): *m/z* $[M + H]^+$ calcd for $[C_{16}H_{24}NO_3]^+$: 278.1751; found: 278.1751.

2-isopropoxyphenyl phenylcarbamate (**3f**). White solid. 384mg, 71% yield. $C_{16}H_{17}NO_3$; M W: 271.32. ^{1}H NMR (500 MHz, $CDCl_3$) δ 7.52 – 7.46 (m, 1H), 7.45 (s, 1H), 7.34 (d, J = 7.3 Hz, 1H), 7.32 (d, J = 7.4 Hz, 1H), 7.20 (d, J = 7.9 Hz, 1H), 7.18 – 7.15 (m, 1H), 7.12 – 7.08 (m, 2H), 7.00 (d, J = 6.8 Hz, 1H), 6.95 (t, J = 7.7 Hz, 1H), 4.56 (hept, J = 6.1 Hz, 1H), 1.33 (d, J = 6.0 Hz, 6H, $2CH_3$). ^{13}C NMR (126 MHz, $CDCl_3$) δ 151.23 (C=O), 149.90, 140.56, 137.54, 128.95 (2C), 126.51, 123.54, 123.28 (2C), 120.60, 118.46, 115.18, 71.31, 21.97 (2C). TOF-HRMS (ESI): *m/z* $[M + H]^+$ calcd for $[C_{16}H_{18}NO_3]^+$: 272.1281; found: 272.1291.

naphthalen-1-yl methylcarbamate (**4a**). White solid. 307mg, 76% yield. $C_{12}H_{11}NO_2$; M W: 201.22. ^{1}H NMR (400 MHz, $CDCl_3$) δ 7.98 – 7.93 (m, 1H), 7.89 – 7.84 (m, 1H), 7.72 (d, J = 8.2 Hz, 1H), 7.52 (d, J = 5.5 Hz, 1H), 7.50 (d, J = 4.9 Hz, 1H), 7.48 – 7.44 (m, 1H), 7.30 (d, J = 7.5 Hz, 1H), 5.21 (s, 1H), 2.95 (d, J = 4.9 Hz, 3H, CH_3). TOF-HRMS (ESI): *m/z* $[M + H]^+$ calcd for $[C_{12}H_{12}NO_2]^+$: 202.0863; found: 202.0785.

naphthalen-1-yl cyclopropylcarbamate (**4b**). White solid. 381mg, 84% yield. $C_{14}H_{13}NO_2$; M W: 227.26. ^{1}H NMR (400 MHz, DMSO) δ 8.26 (d, J = 3.3 Hz, 1H), 8.02 – 7.95 (m, 1H), 7.92 – 7.86 (m, 1H), 7.80 (d, J = 8.2 Hz, 1H), 7.58 (d, J = 2.5 Hz, 1H), 7.57 – 7.53 (m, 1H), 7.51 (t, J = 7.9 Hz, 1H), 7.30 (d, J = 7.5 Hz, 1H), 2.63 (tq, J = 7.2, 3.6 Hz, 1H), 0.67 (dt, J = 6.8, 3.3 Hz, 2H), 0.57 (dd, J = 5.1, 2.4 Hz, 2H). ^{13}C NMR (101 MHz, DMSO) δ 155.20 (C=O), 146.69, 134.16, 127.97, 127.28, 126.49 (2C), 125.80, 125.19, 121.15, 118.58, 23.24, 5.98 (2C). TOF-HRMS (ESI): *m/z* $[M + H]^+$ calcd for $[C_{14}H_{14}NO_2]^+$: 228.1019; found: 228.0999.

naphthalen-1-yl cyclobutylcarbamate (**4c**). White solid. 379mg, 79% yield.

$C_{15}H_{15}NO_2$; M W: 241.29. ^{1}H NMR (500 MHz, DMSO) δ 8.36 (d, J = 8.0 Hz, 1H), 7.99 – 7.96 (m, 1H), 7.88 (d, J = 6.6 Hz, 1H), 7.80 (d, J = 8.2 Hz, 1H), 7.60 – 7.57 (m, 1H), 7.57 – 7.54 (m, 1H), 7.52 – 7.47 (m, 1H), 7.29 (d, J = 7.5 Hz, 1H), 4.06 (h, J = 8.0 Hz, 1H), 2.28 – 2.19 (m, 2H), 2.09 – 1.99 (m, 2H), 1.64 (dd, J = 24.9, 10.0 Hz, 2H). ^{13}C NMR (126 MHz, DMSO) δ 153.76 (C=O), 147.10, 134.57, 128.38, 127.73, 126.90, 126.88, 126.20, 125.56, 121.53, 118.99, 46.34, 30.69 (2C), 14.86. TOF-HRMS (ESI): *m/z* $[M + H]^+$ calcd for $[C_{15}H_{16}NO_2]^+$: 242.1176; found: 242.1178.

naphthalen-1-yl cyclopentylcarbamate (**4d**). White solid. 448mg, 88% yield. $C_{16}H_{17}NO_2$; M W: 255.32. ^{1}H NMR (400 MHz, DMSO) δ 8.07 (d, J = 7.2 Hz, 1H), 7.97 (d, J = 8.9 Hz, 1H), 7.90 (d, J = 7.3 Hz, 1H), 7.79 (d, J = 8.2 Hz, 1H), 7.61 – 7.53 (m, 2H), 7.50 (t, J = 7.9 Hz, 1H), 7.30 (d, J = 7.6 Hz, 1H), 3.89 (h, J = 7.2 Hz, 1H), 1.87 (dd, J = 9.2, 5.4 Hz, 2H), 1.70 (d, J = 7.7 Hz, 2H), 1.61 – 1.48 (m, 4H). ^{13}C NMR (101 MHz, DMSO) δ 153.95 (C=O), 146.79, 134.13, 127.92, 127.33, 126.41 (2C), 125.76, 125.02, 121.14, 118.50, 52.46, 32.31 (2C), 23.37 (2C). TOF-HRMS (ESI): *m/z* $[M + H]^+$ calcd for $[C_{16}H_{18}NO_2]^+$: 256.1332; found: 256.1311.

naphthalen-1-yl cyclohexylcarbamate (**4e**). White solid. 441mg, 82% yield. $C_{17}H_{19}NO_2$; M W: 269.34. ^{1}H NMR (400 MHz, DMSO) δ 7.97 (dd, J = 6.3, 3.1 Hz, 2H), 7.79 (d, J = 8.2 Hz, 1H), 7.59 – 7.53 (m, 1H), 7.50 (t, J = 7.9 Hz, 2H), 7.49 (t, 1H), 7.29 (dd, J = 7.5, 0.8 Hz, 1H), 3.35 (d, J = 7.7 Hz, 1H), 1.89 (d, J = 8.1 Hz, 2H), 1.80 – 1.67 (m, 3H), 1.39 – 1.07 (m, 5H). ^{13}C NMR (101 MHz, DMSO) δ 153.61 (C=O), 146.79, 134.10, 127.87, 127.29, 126.35, 126.32, 125.70, 124.95, 121.10, 118.39, 49.83, 32.54, 25.13 (2C), 24.52 (2C). TOF-HRMS (ESI): *m/z* $[M + H]^+$ calcd for $[C_{17}H_{20}NO_2]^+$: 270.1489; found: 270.1450.

naphthalen-1-yl phenylcarbamate (**4f**). White solid. 396mg, 75% yield. $C_{17}H_{13}NO_2$; M W: 263.30. ^{1}H NMR (400 MHz, $CDCl_3$) δ 10.13 (d, J = 1.6 Hz, 1H), 8.67 (s, 1H), 8.14 (d, J = 7.8 Hz, 1H), 7.81 (d, J = 8.4 Hz, 1H), 7.57 (d, J = 8.8 Hz, 1H), 7.49 – 7.46

(m, 1H), 7.46 – 7.44 (m, 1H), 7.43 (d, *J* = 1.6 Hz, 1H), 7.33 – 7.31 (m, 1H), 7.30 (d, *J* = 1.6 Hz, 1H), 7.28 (d, *J* = 2.2 Hz, 1H), 6.97 (t, *J* = 7.4 Hz, 1H), 6.88 (d, *J* = 7.0 Hz, 1H). ^{13}C NMR (101 MHz, $CDCl_3$) *δ* 153.19 (C=O), 152.58, 139.75, 134.45, 128.84 (2C), 127.41, 126.46, 126.12, 124.58, 122.00 (2C), 121.86, 118.35, 118.22, 113.90, 108.05. TOF-HRMS (ESI): *m/z* $[M + H]^+$ calcd for $[C_{17}H_{14}NO_2]^+$: 264.1019; found: 264.1017.

2,2-dimethyl-2,3-dihydrobenzofuran-7-yl methylcarbamate (**5a**). White solid. 358 mg, 81% yield. $C_{12}H_{15}NO_3$; M W: 221.26. ^{1}H NMR (400 MHz, $CDCl_3$) *δ* 6.97 (dd, *J* = 7.3, 1.2 Hz, 1H), 6.94 (d, *J* = 7.5 Hz, 1H), 6.82 – 6.74 (m, 1H), 5.03 (s, 1H), 3.03 (d, *J* = 1.0 Hz, 2H), 2.88 (d, *J* = 4.9 Hz, 3H, CH_3), 1.49 (s, 6H, 2CH_3). TOF-HRMS (ESI): *m/z* $[M + H]^+$ calcd for $[C_{12}H_{16}NO_3]^+$: 222.1125; found: 222.1124.

2,2-dimethyl-2,3-dihydrobenzofuran-7-yl cyclopropylcarbamate (**5b**). White solid. 420 mg, 85% yield. $C_{14}H_{17}NO_3$; M W: 247.29. ^{1}H NMR (600 MHz, $CDCl_3$) *δ* 6.93 (t, *J* = 7.3 Hz, 2H), 6.75 (t, 1H), 5.29(s, 1H), 3.01 (s, 2H), 2.66 (s, 1H), 1.46 (s, 6H, 2CH_3), 0.79 – 0.69 (m, 2H), 0.60 (s, 2H). ^{13}C NMR (151 MHz, $CDCl_3$) *δ* 154.79 (C=O), 150.32, 134.93, 129.60, 122.20, 121.95, 120.22, 88.37, 43.28, 28.33 (2C), 23.46, 6.95 (2C). TOF-HRMS (ESI): *m/z* $[M + H]^+$ calcd for $[C_{14}H_{18}NO_3]^+$: 248.1281; found: 248.1281.

2,2-dimethyl-2,3-dihydrobenzofuran-7-yl cyclobutylcarbamate (**5c**). White solid. 397mg, 76% yield. $C_{15}H_{19}NO_3$; M W: 261.32. ^{1}H NMR (600 MHz, $CDCl_3$) *δ* 7.00 – 6.91 (m, 2H), 6.77 (t, *J* = 7.7 Hz, 1H), 5.26 (d, 1H), 4.19 – 4.24 (m, 1H),3.02 (s, 2H), 2.36 (q, *J* = 7.8 Hz, 2H), 1.99 – 1.85 (m, 2H), 1.77 – 1.60 (m, 2H), 1.48 (s, 6H, 2CH_3). ^{13}C NMR (151 MHz, $CDCl_3$) *δ* 153.04 (C=O), 150.34, 134.94, 129.58, 122.16, 122.03, 120.22, 88.34, 46.61, 43.28, 31.44 (2C), 28.33 (2C), 14.99. TOF-HRMS (ESI): *m/z* $[M + H]^+$ calcd for $[C_{15}H_{20}NO_3]^+$: 262.1438; found: 262.1437.

2,2-dimethyl-2,3-dihydrobenzofuran-7-yl cyclopentylcarbamate (**5d**). White solid.

473 mg, 86% yield. $C_{16}H_{21}NO_3$; M W: 275.35. ^{1}H NMR (500 MHz, $CDCl_3$) δ 6.97 (d, J = 7.3 Hz, 1H), 6.95 (d, J = 1.1 Hz, 1H), 6.77 (t, J = 7.7 Hz, 1H), 5.24 – 4.81 (m, 1H), 4.04 (h, J = 6.8 Hz, 1H), 3.03 (s, 2H), 2.00 (dq, J = 12.7, 6.8 Hz, 2H), 1.69 (dd, J = 7.5, 4.3 Hz, 2H), 1.64 – 1.57 (m, 2H), 1.51 (d, J = 5.5 Hz, 2H), 1.49 (s, 6H, $2CH_3$). ^{13}C NMR (126 MHz, $CDCl_3$) δ 153.85 (C=O), 150.45, 135.23, 129.73, 122.24, 122.17, 120.40, 88.51, 53.43, 43.45, 33.43 (2C), 28.52 (2C), 23.89 (2C). TOF-HRMS (ESI): *m/z* $[M + H]^+$ calcd for $[C_{16}H_{22}NO_3]^+$: 276.1594; found: 276.1597.

2,2-dimethyl-2,3-dihydrobenzofuran-7-yl cyclohexylcarbamate (**5e**). White solid. 462 mg, 73% yield. $C_{17}H_{23}NO_3$; M W: 289.38. ^{1}H NMR (500 MHz, $CDCl_3$) δ 6.97 (d, J = 5.2 Hz, 1H), 6.95 (d, J = 5.0 Hz, 1H), 6.77 (t, J = 7.7 Hz, 1H), 5.00 (d, J = 8.0 Hz, 1H), 3.64 – 3.49 (m, 1H), 3.03 (s, 2H), 2.01 (dd, J = 12.9, 3.9 Hz, 2H), 1.72 (dd, J = 9.6, 4.1 Hz, 2H), 1.68 – 1.55 (m, 2H), 1.49 (s, 6H, $2CH_3$), 1.40 – 1.31 (m, 2H), 1.22 – 1.17 (m, 2H). ^{13}C NMR (126 MHz, $CDCl_3$) δ 153.24 (C=O), 150.23, 135.02, 129.47, 121.97, 121.90, 120.14, 88.24, 50.30, 43.21, 33.28 (2C), 28.28 (2C), 25.60, 24.87 (2C). TOF-HRMS (ESI): *m/z* $[M + H]^+$ calcd for $[C_{17}H_{24}NO_3]^+$: 290.1751; found: 290.1751.

2,2-dimethyl-2,3-dihydrobenzofuran-7-yl phenylcarbamate (**5f**). White solid. 413 mg, 73 % yield. $C_{17}H_{17}NO_3$; M W: 283.33. ^{1}H NMR (600 MHz, $CDCl_3$) δ 7.42 (d, J = 7.9 Hz, 2H), 7.30 (t, J = 8.0 Hz, 2H), 7.07 (t, J = 7.4 Hz, 2H), 7.02 – 6.97 (m, 2H), 6.80 (t, 1H), 3.04 (s, 2H), 1.48 (s, 6H, $2CH_3$). ^{13}C NMR (151 MHz, $CDCl_3$) δ 151.15 (C=O), 150.42, 137.75, 134.48, 129.84 (2C), 129.27, 123.89, 122.67, 122.01 (2C), 120.37, 118.82, 88.67, 43.28, 28.33 (2C). TOF-HRMS (ESI): *m/z* $[M + H]^+$ calcd for $[C_{17}H_{18}NO_3]^+$: 284.1281; found: 284.1283.

6. ¹H-NMR and ¹³C-NMR spectra

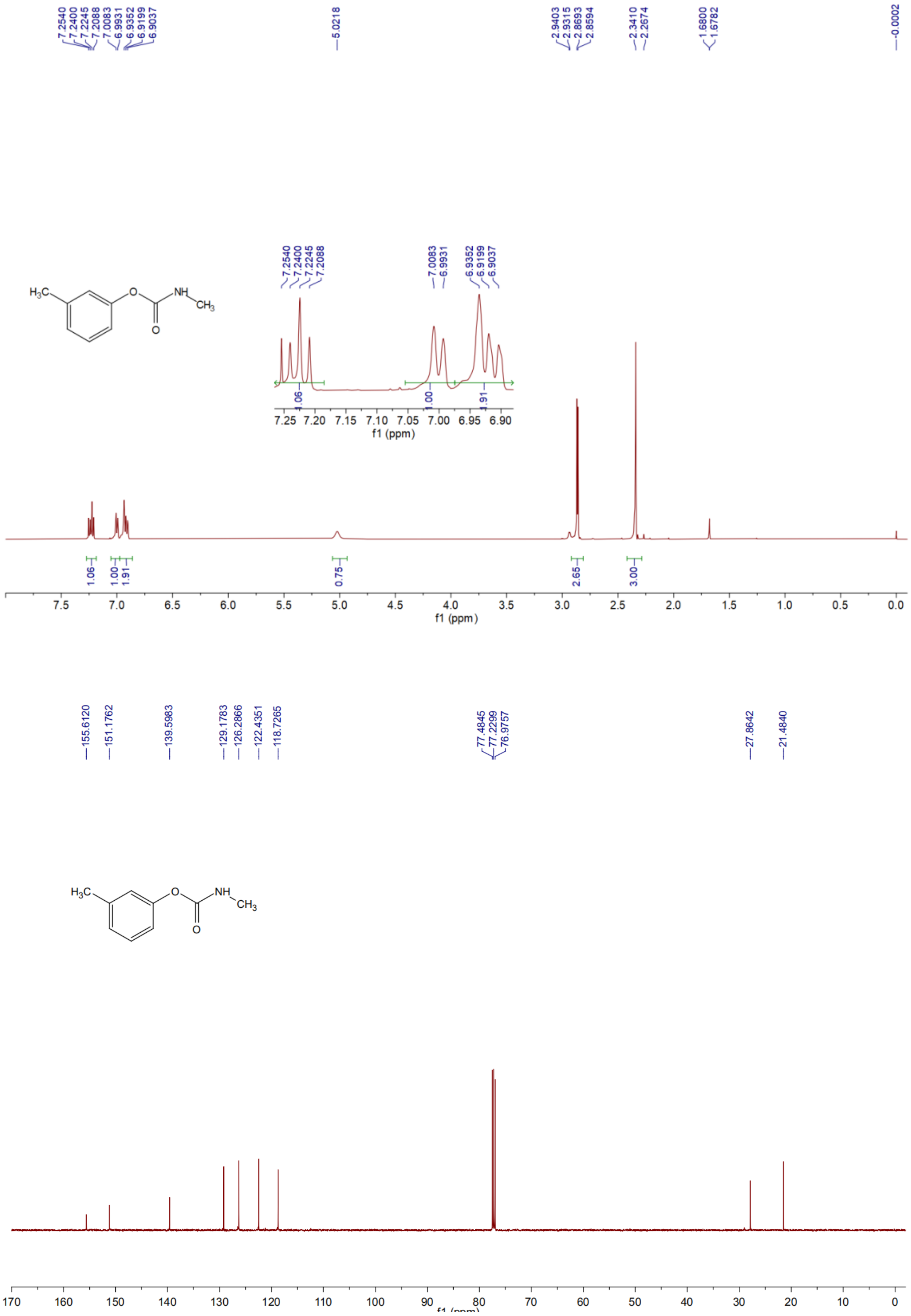

Figure S1. ¹H-NMR and ¹³C-NMR spectra of compound **1a**.

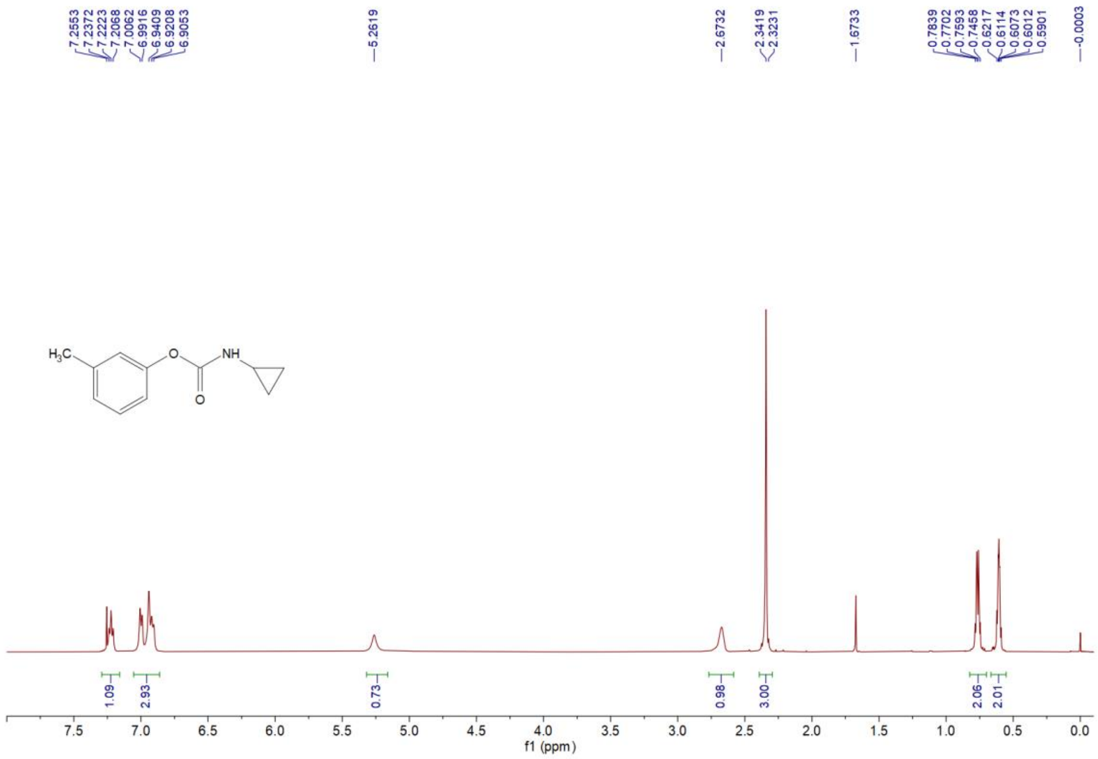

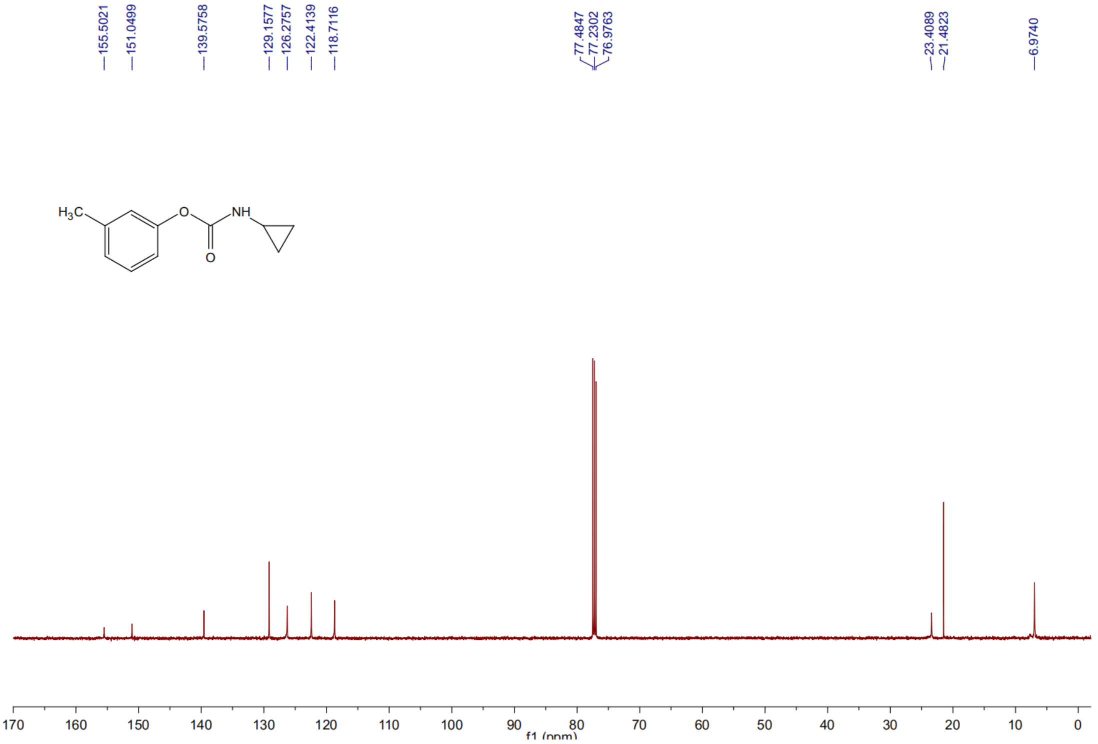

Figure S2. ^{1}H-NMR and ^{13}C-NMR spectra of compound **1b**.

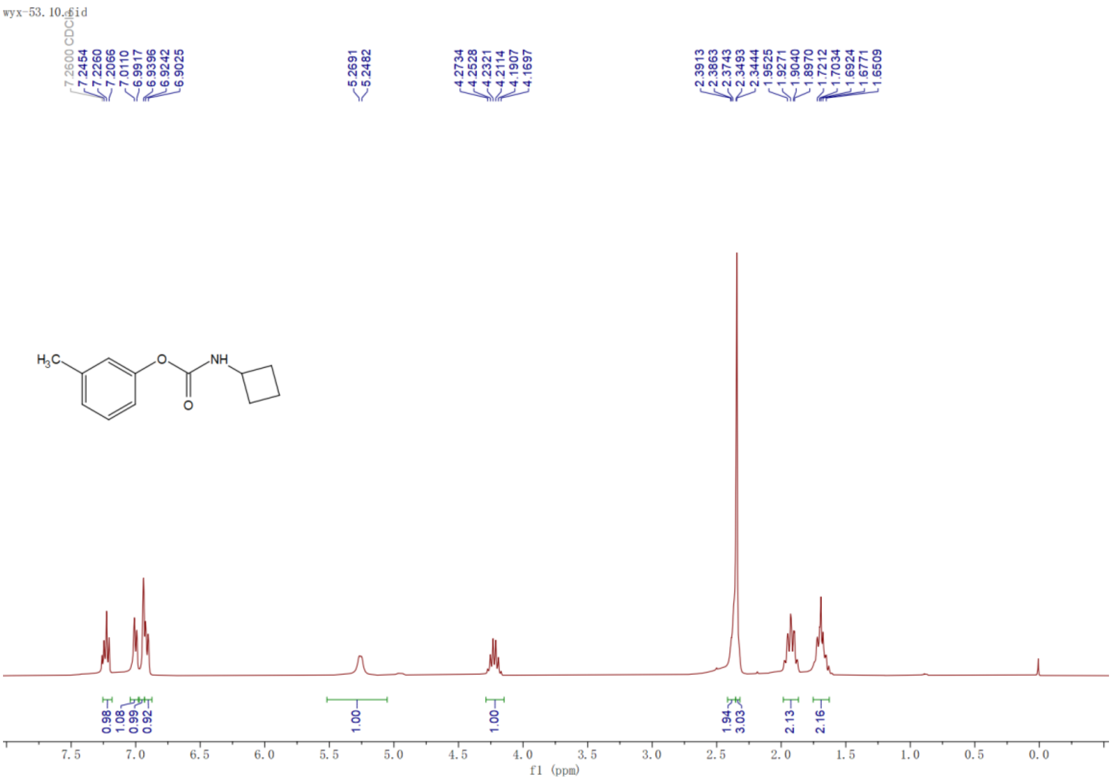

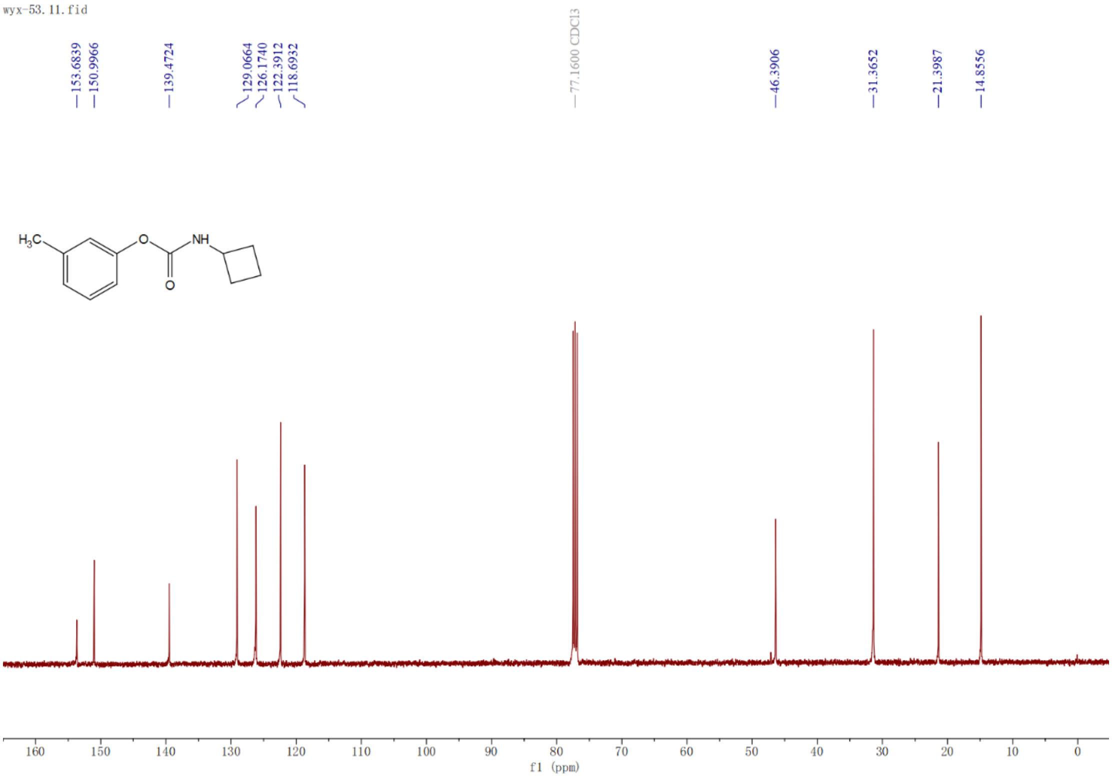

Figure S3. ^{1}H-NMR and ^{13}C-NMR spectra of compound **1c**.

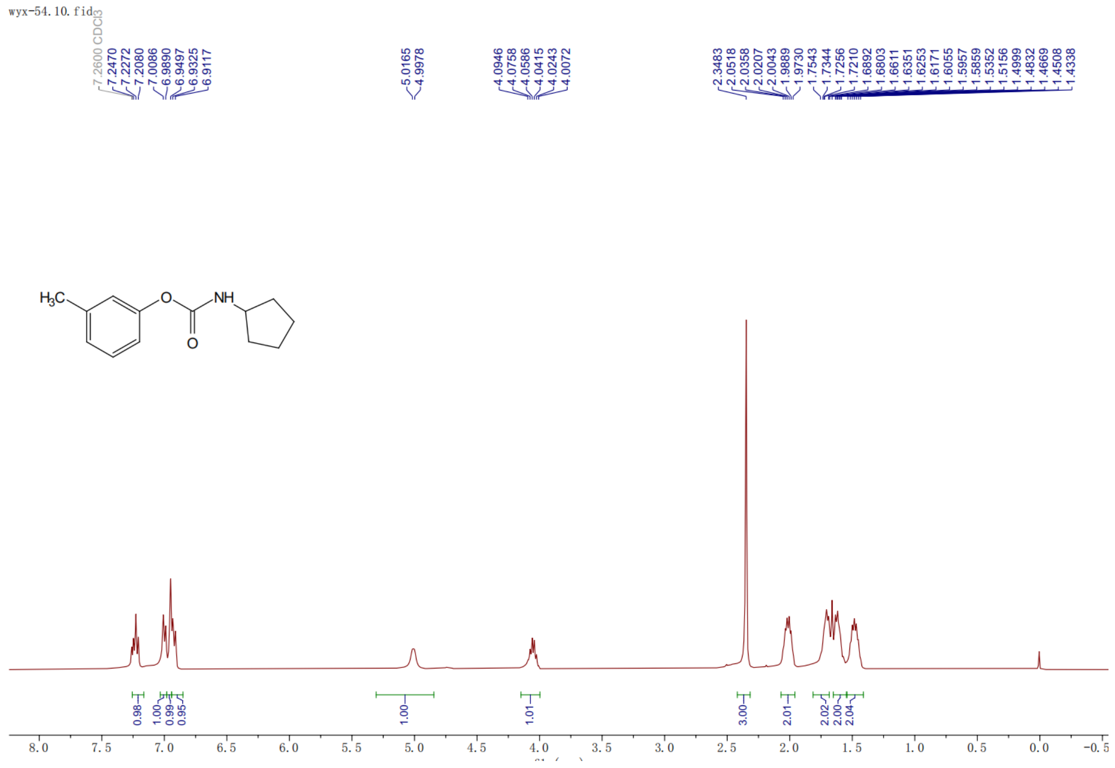

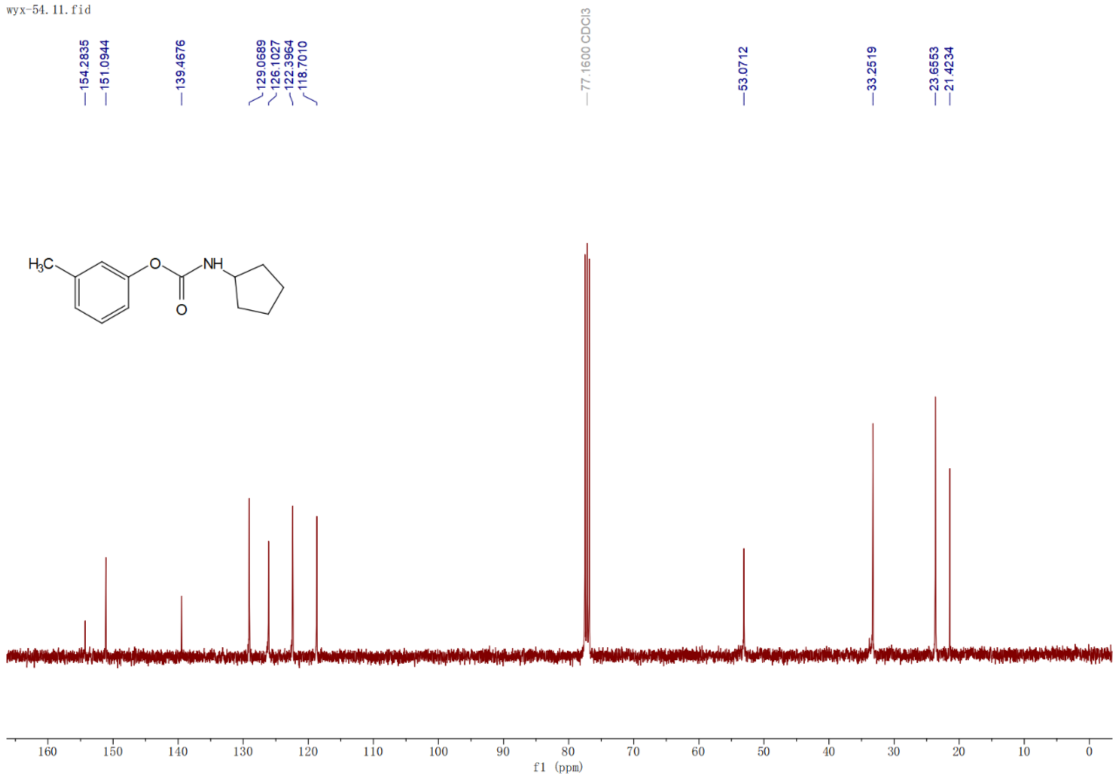

Figure S4. ^{1}H-NMR and ^{13}C-NMR spectra of compound **1d**.

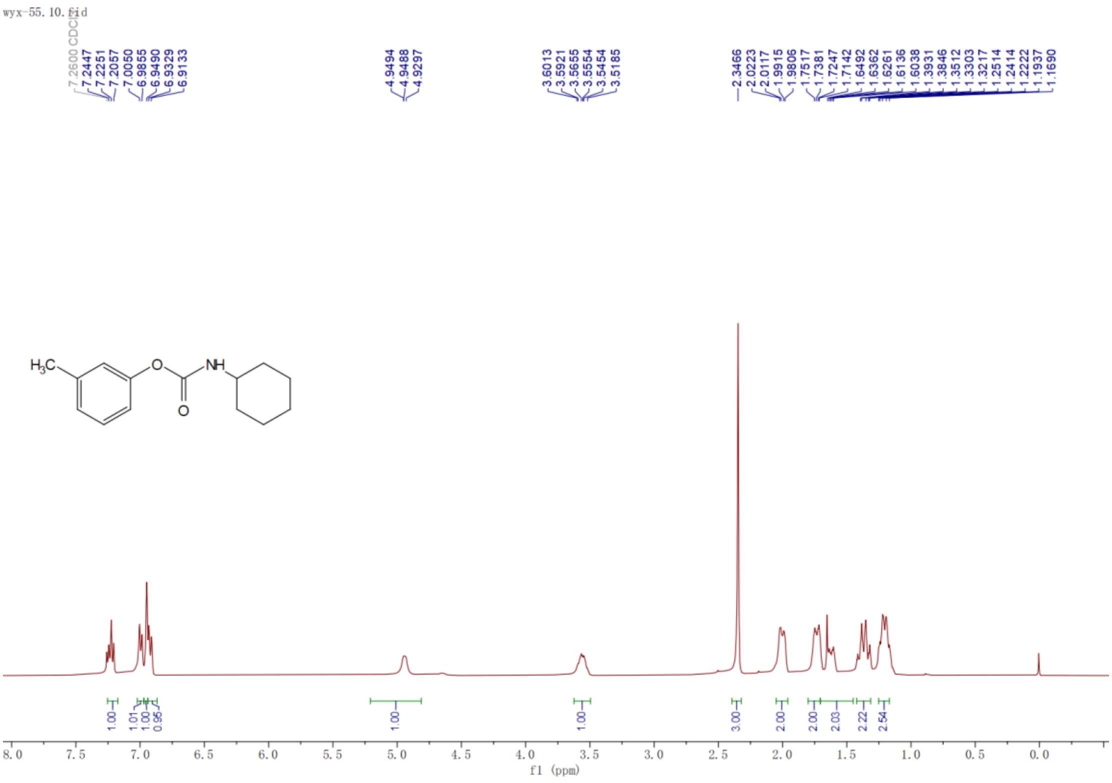

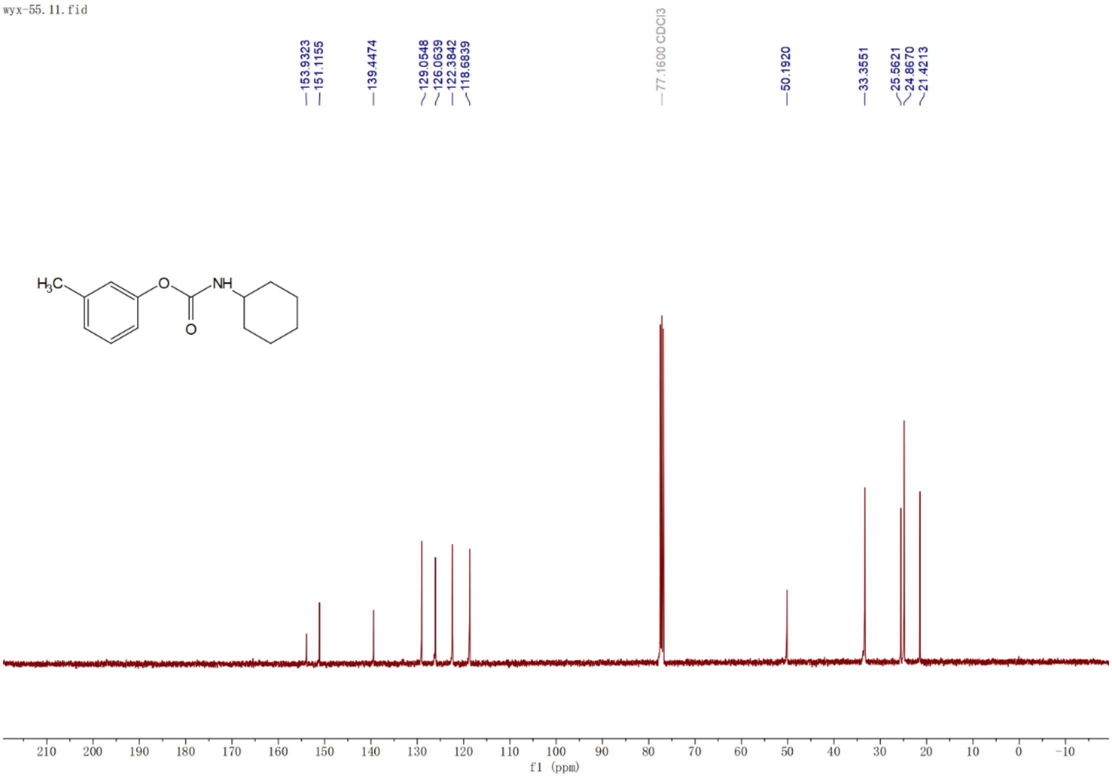

Figure S5. ^{1}H-NMR and ^{13}C-NMR spectra of compound **1e**.

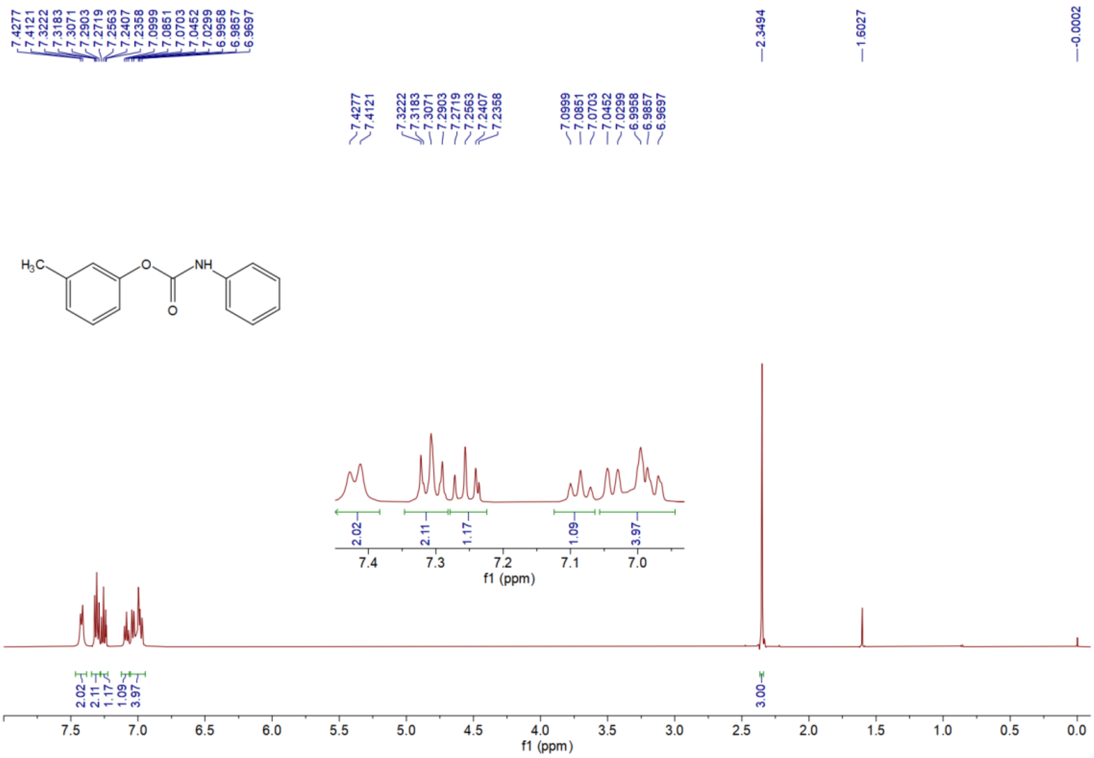

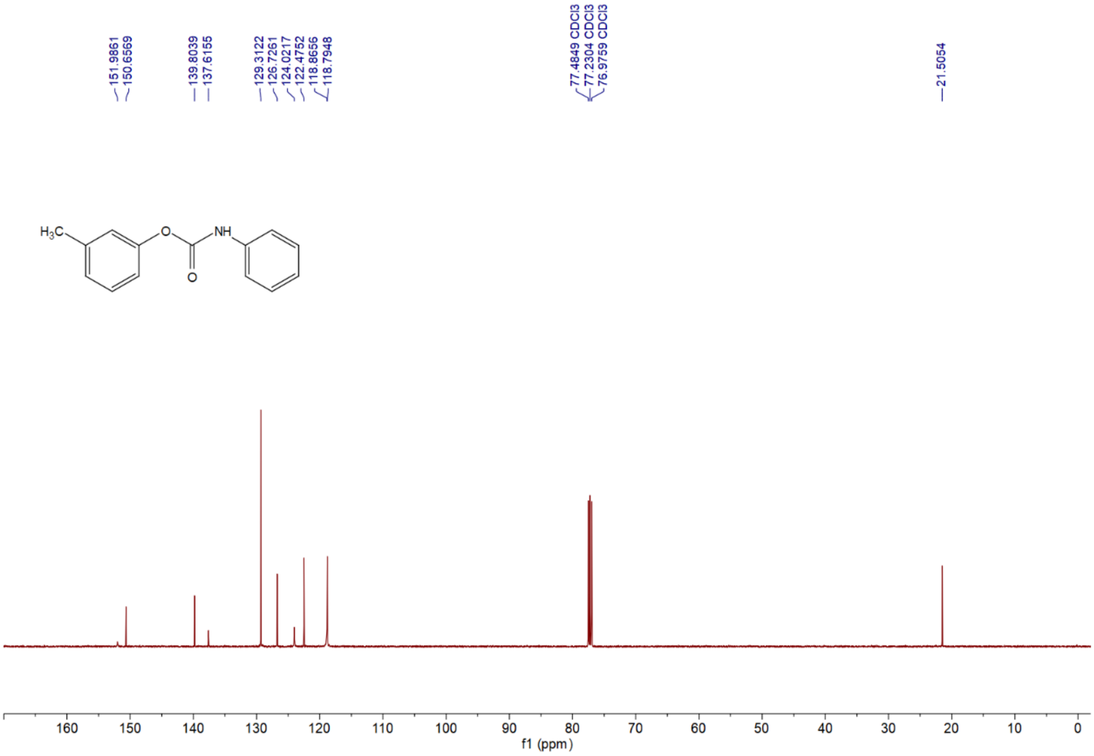

Figure S6. ^{1}H-NMR and ^{13}C-NMR spectra of compound **1f**.

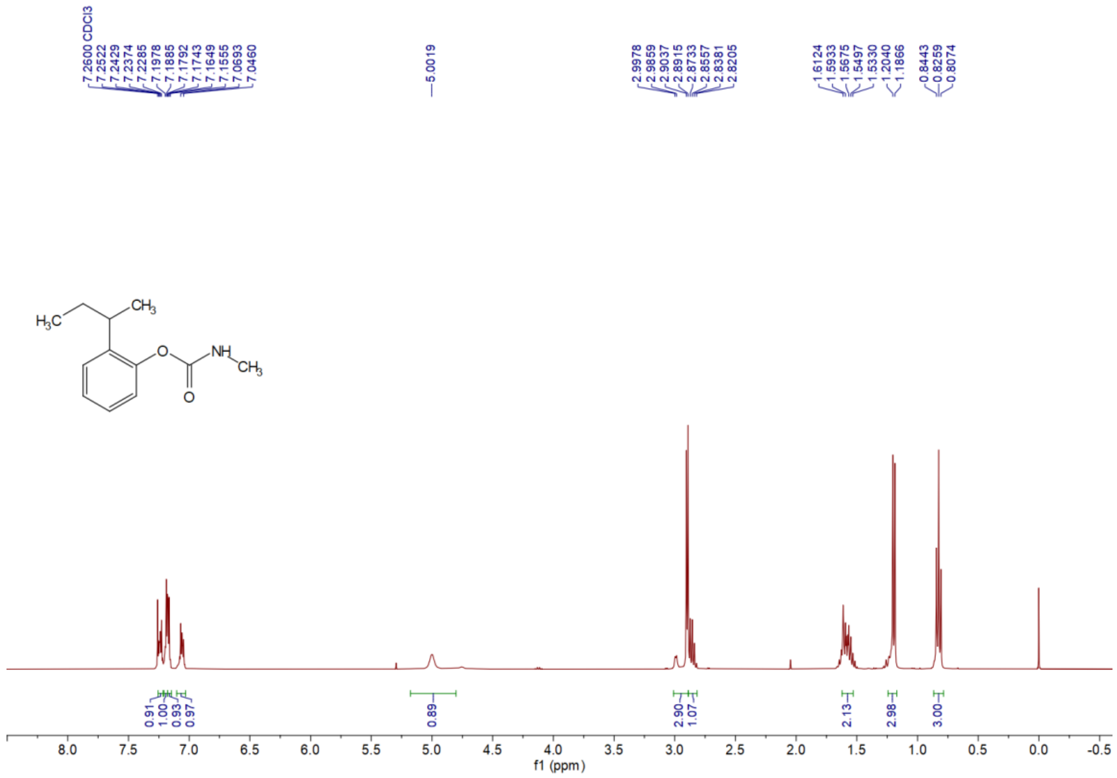

Figure S7. ^{1}H-NMR spectra of compound **2a**.

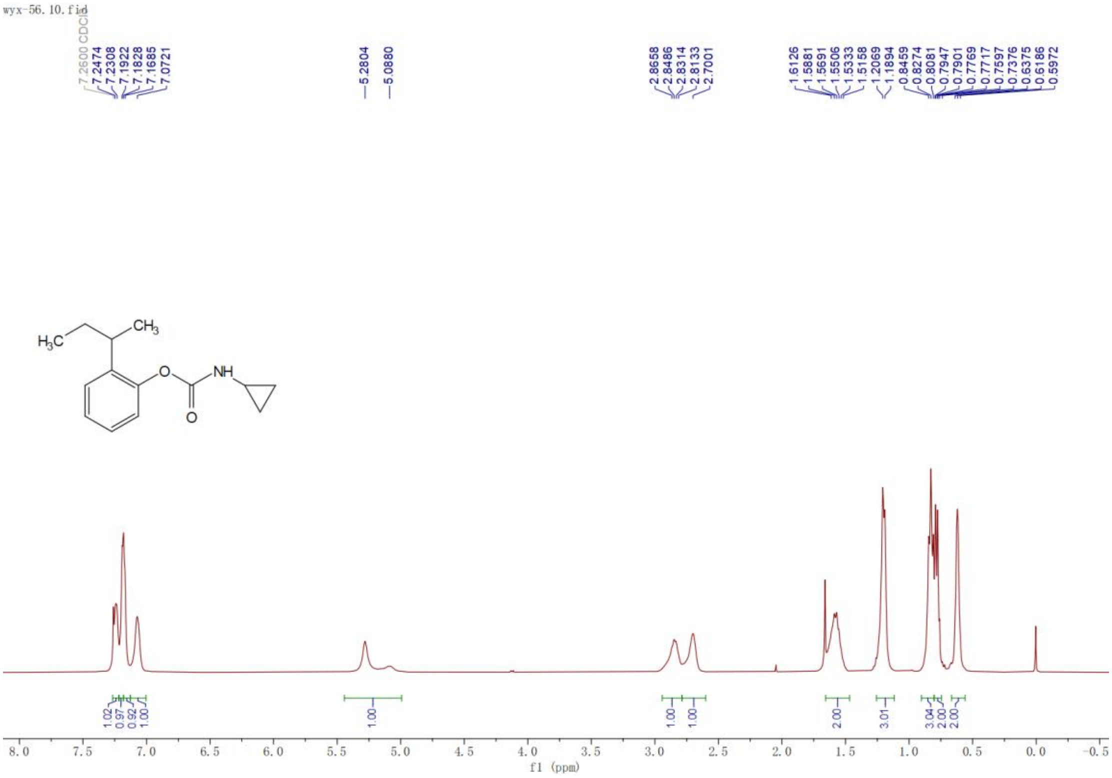

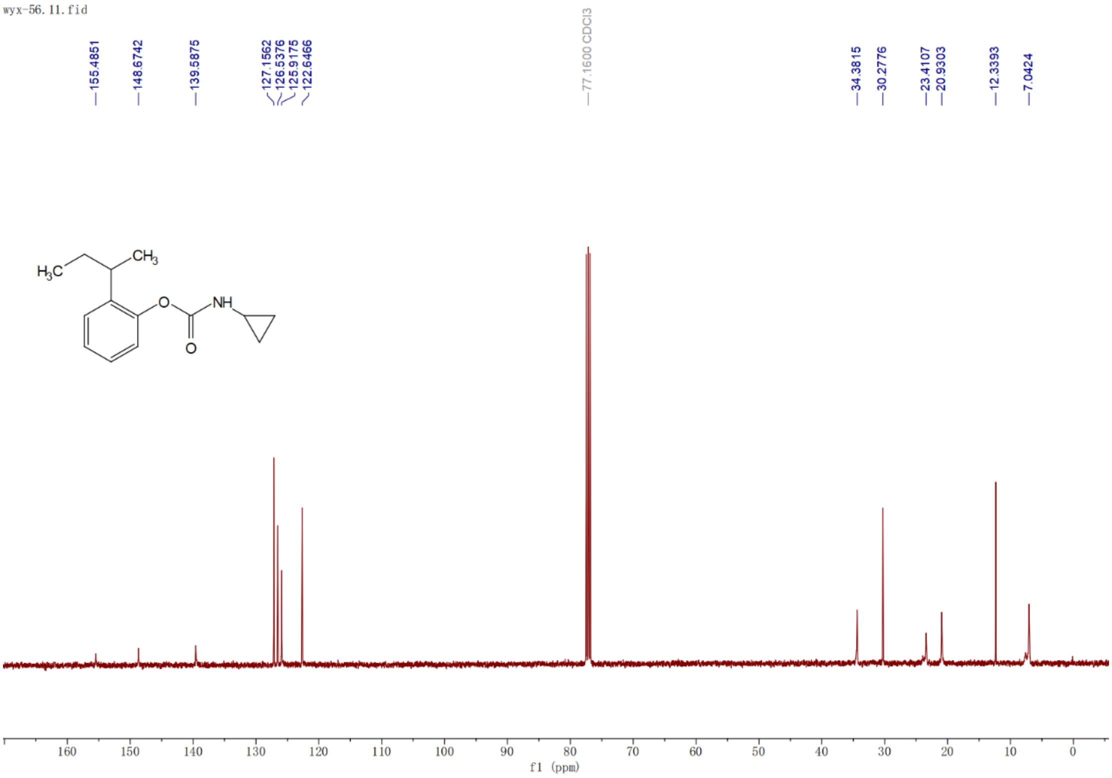

Figure S8. ^{1}H-NMR and ^{13}C-NMR spectra of compound **2b**.

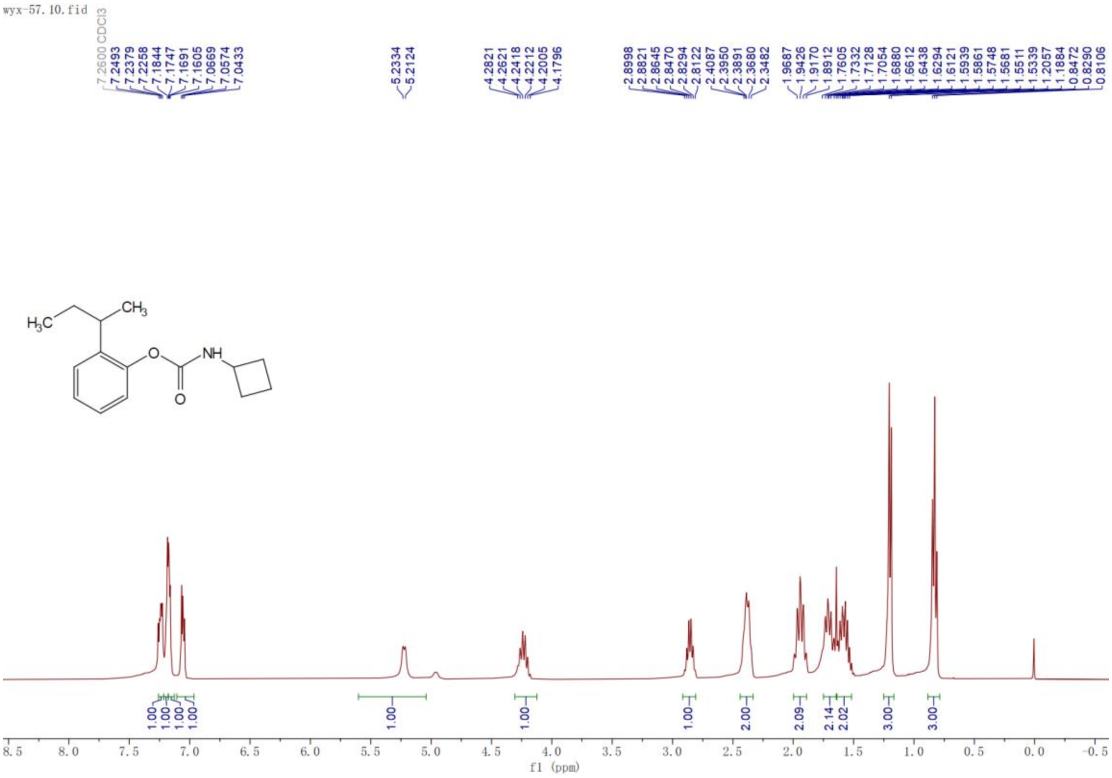

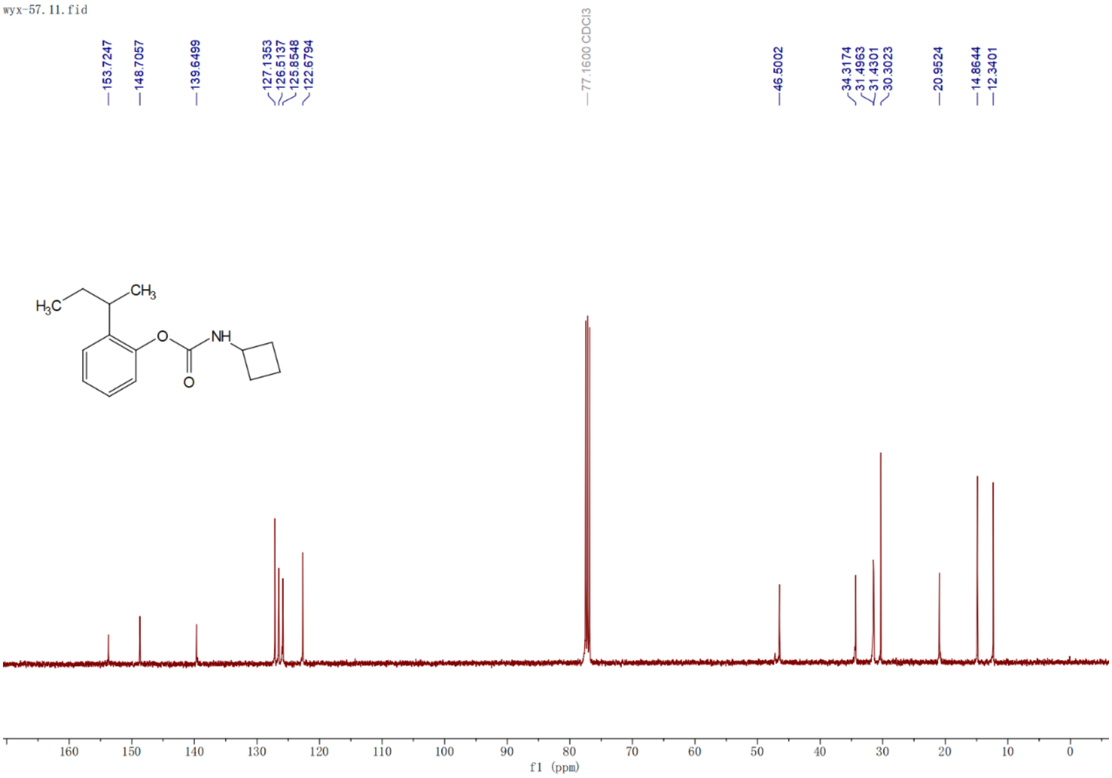

Figure S9. ^{1}H-NMR and ^{13}C-NMR spectra of compound **2c**.

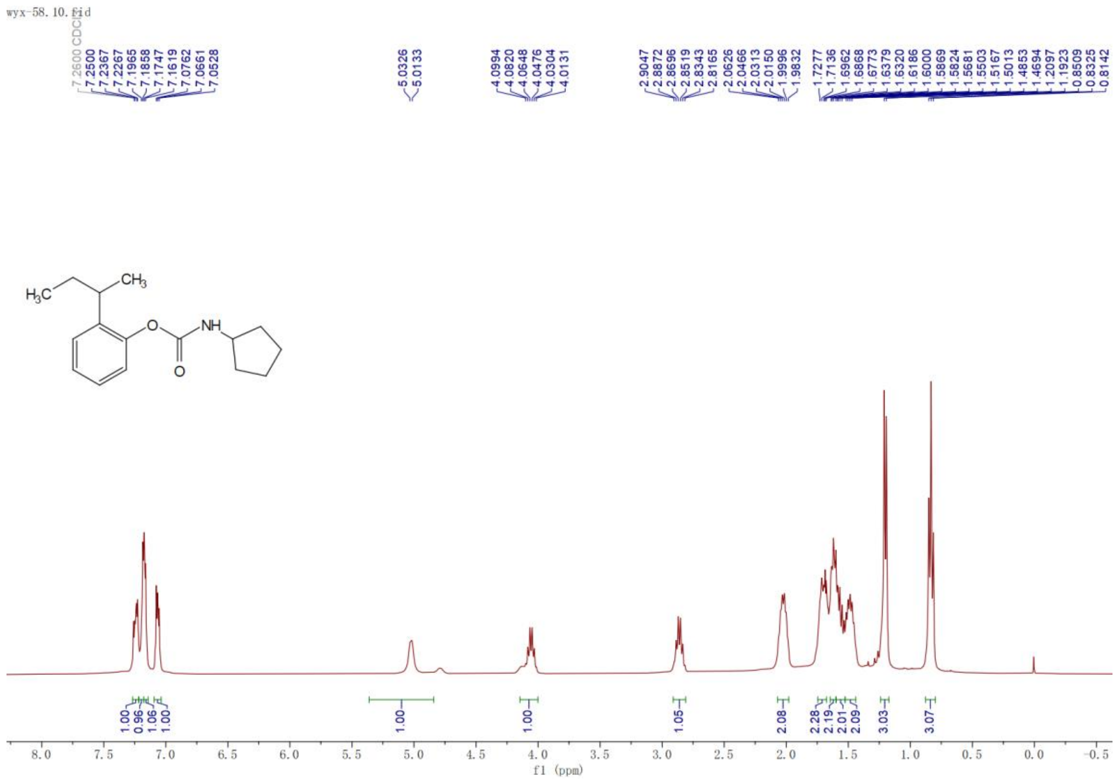

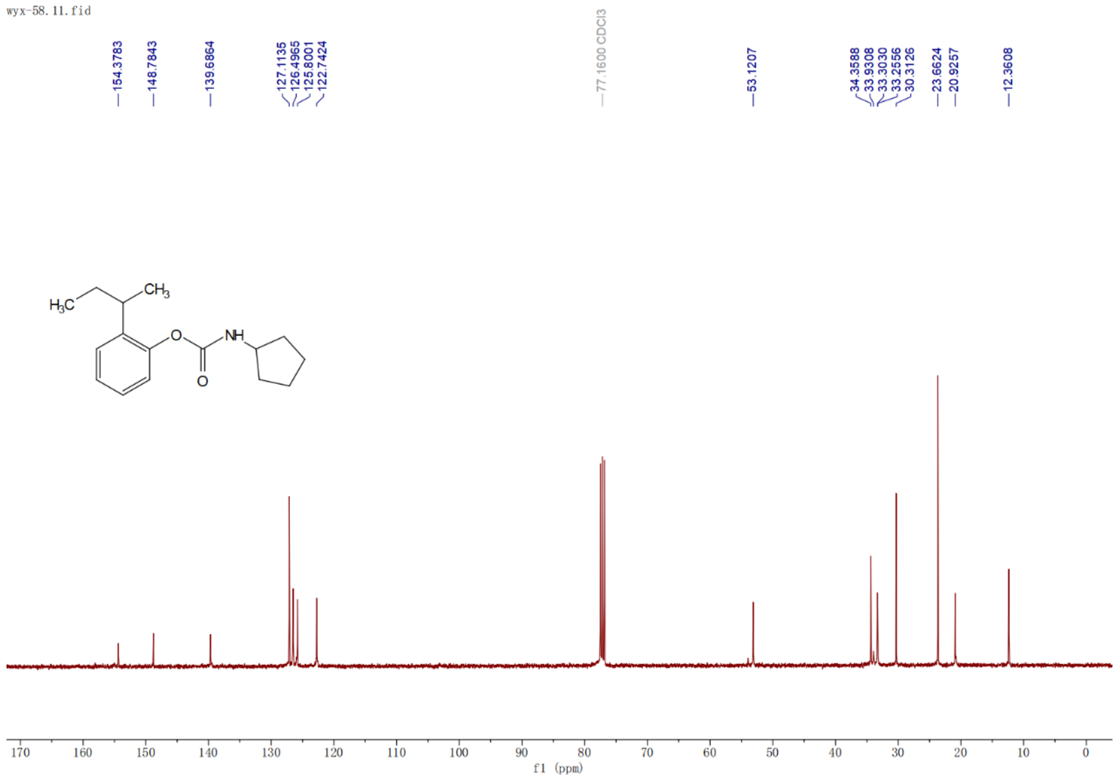

Figure S10. ^{1}H-NMR and ^{13}C-NMR spectra of compound **2d**.

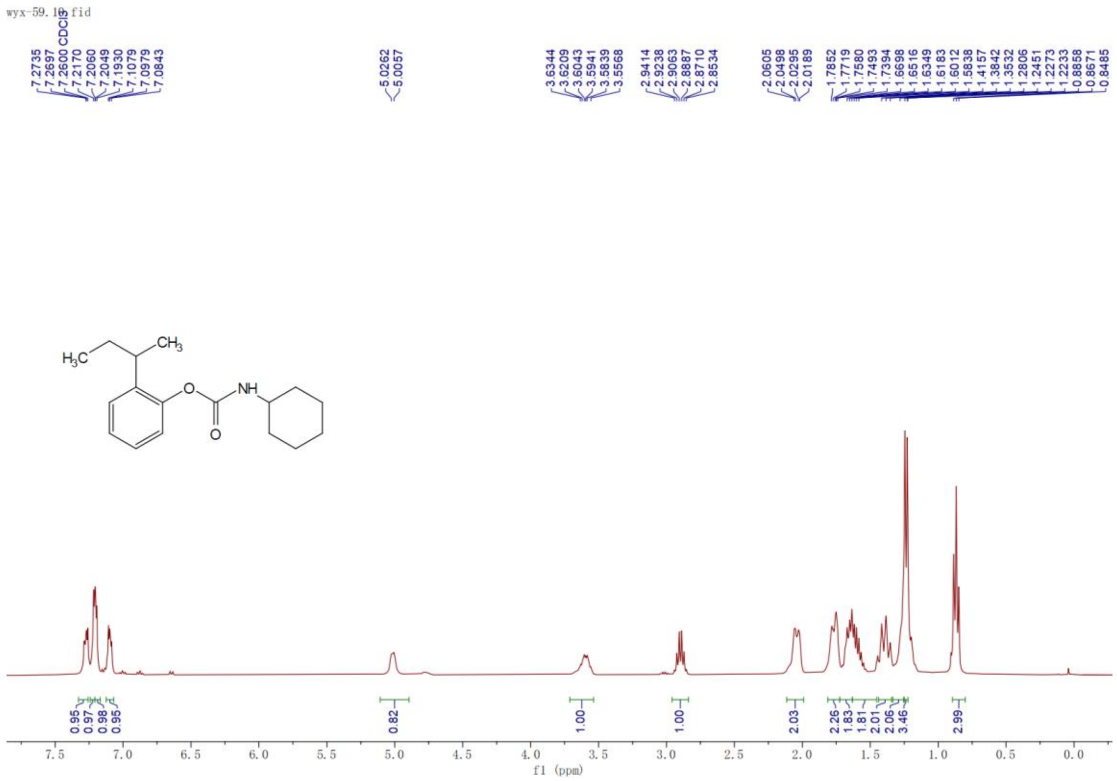

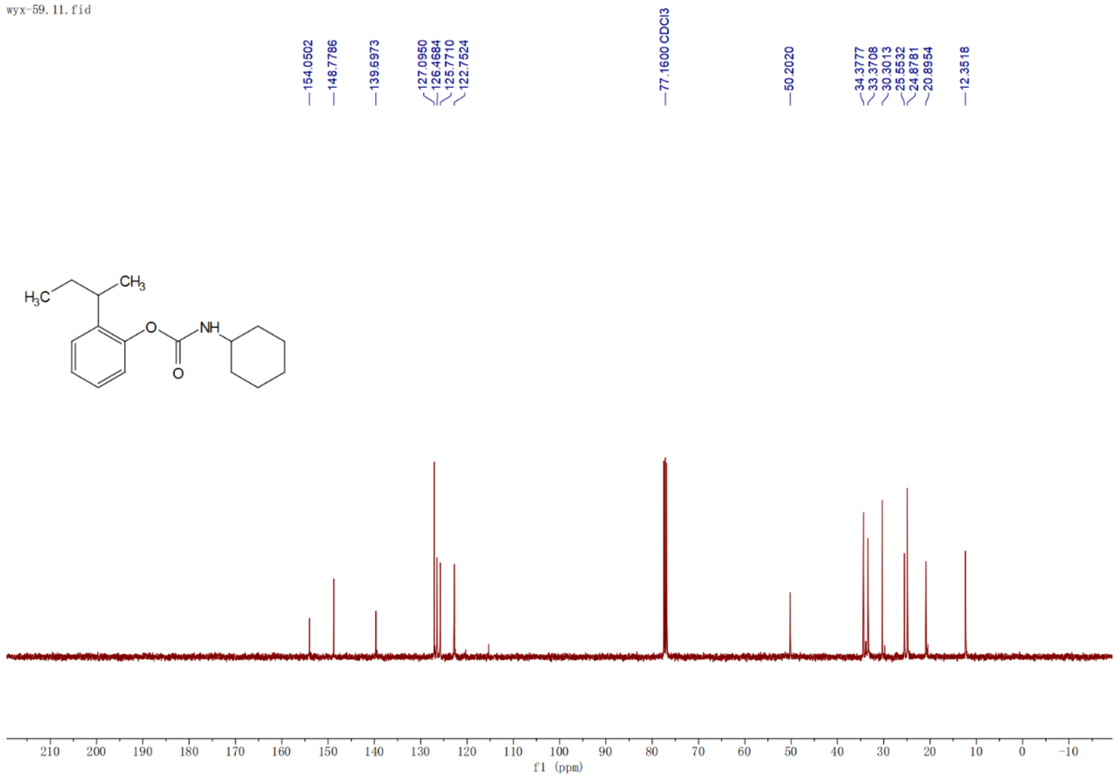

Figure S11. ^{1}H-NMR and ^{13}C-NMR spectra of compound **2e**.

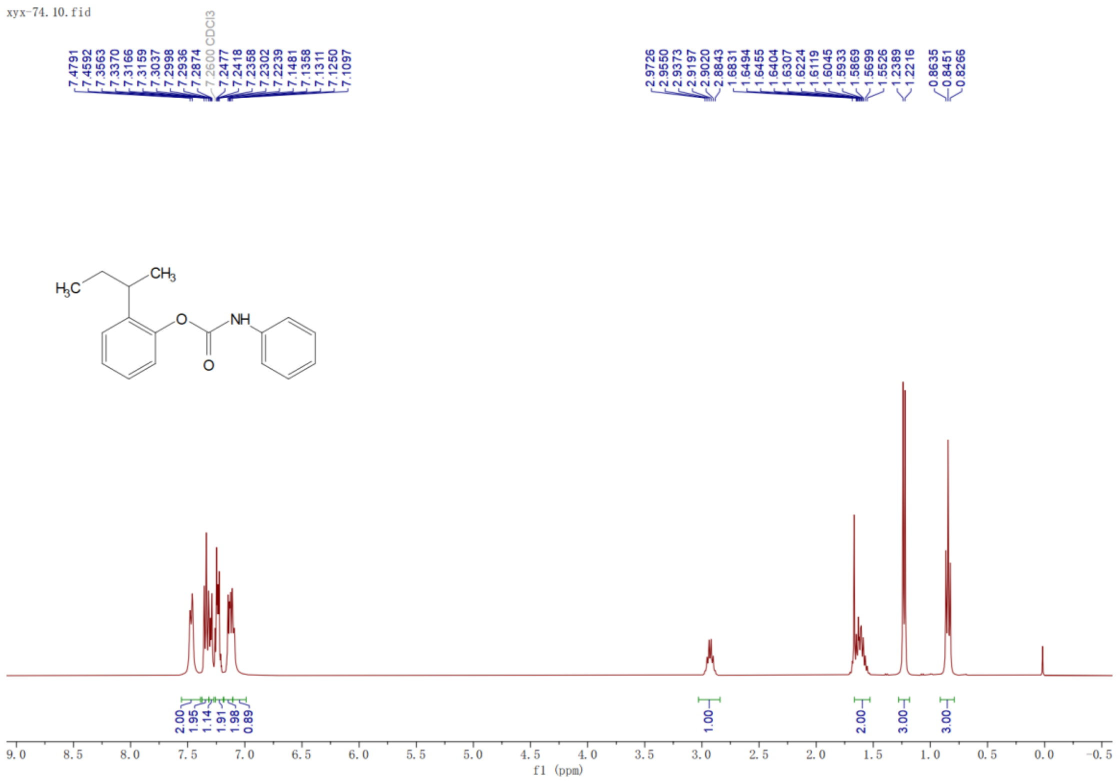

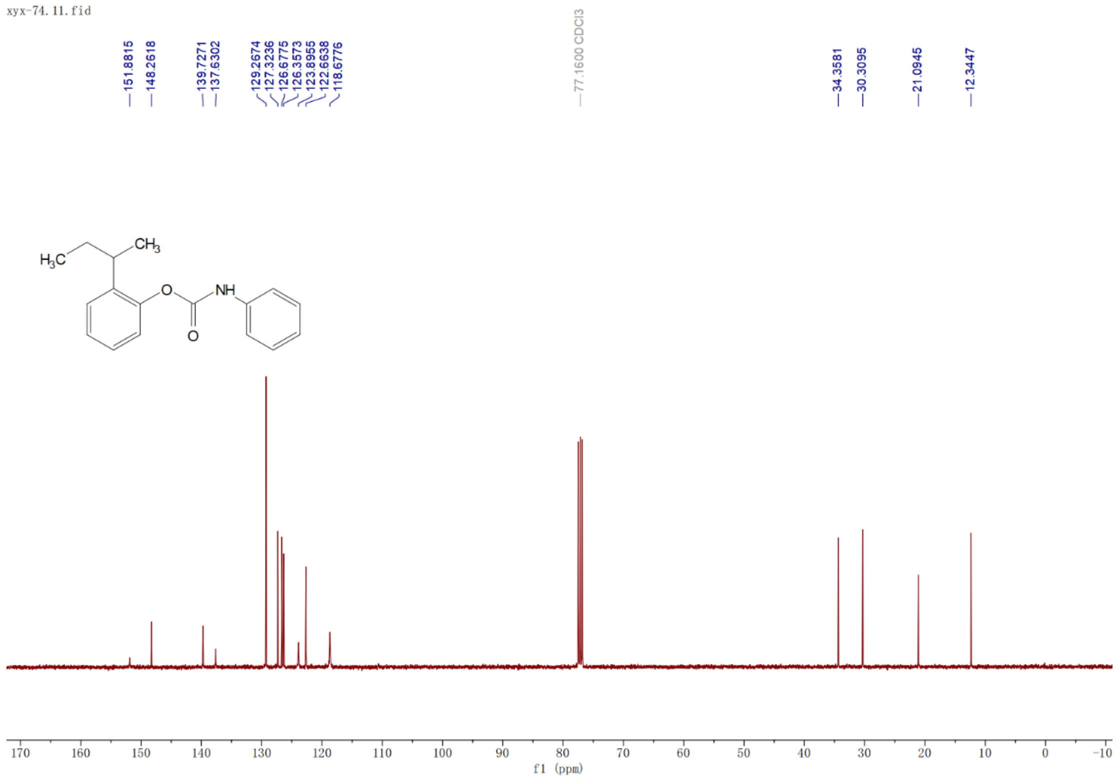

Figure S12. ^{1}H-NMR and ^{13}C-NMR spectra of compound **2f**.

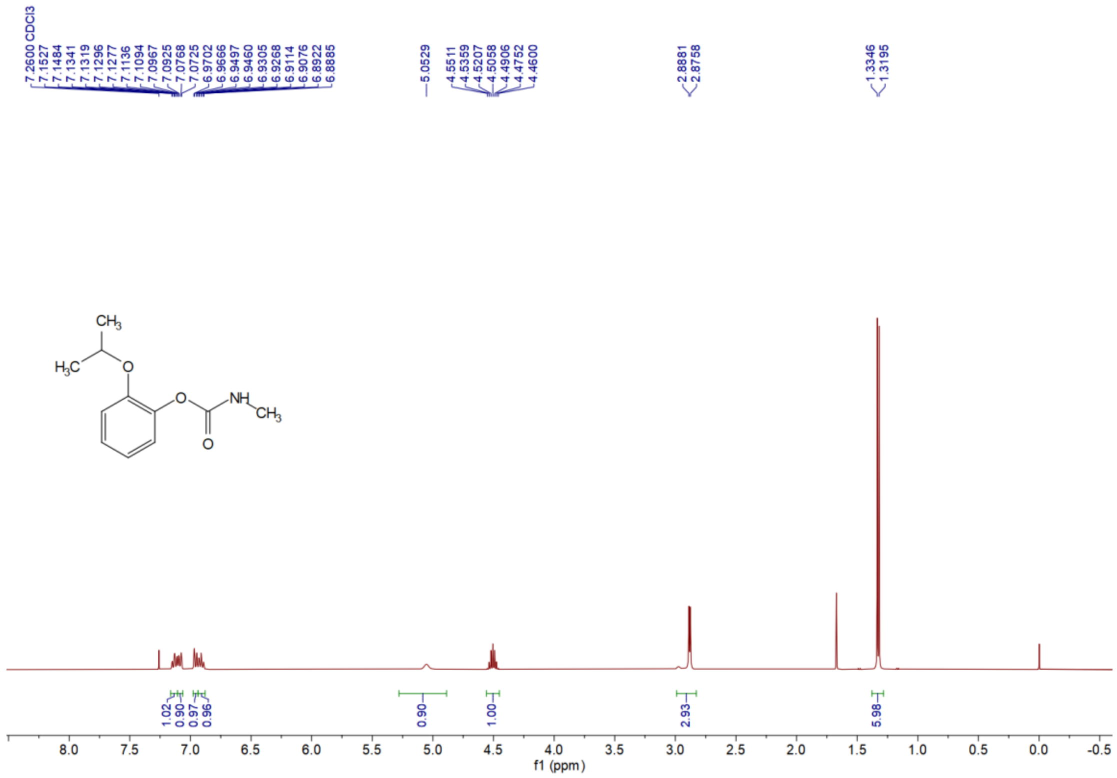

Figure S13. ^{1}H-NMR spectra of compound **3a**.

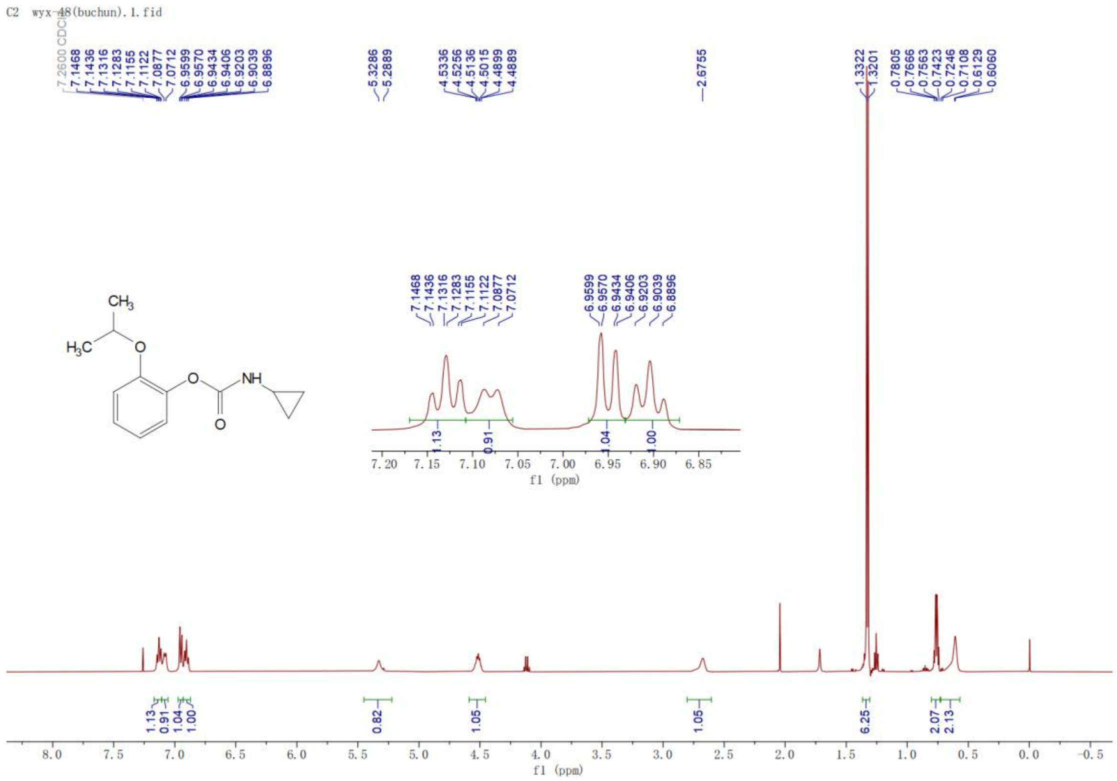

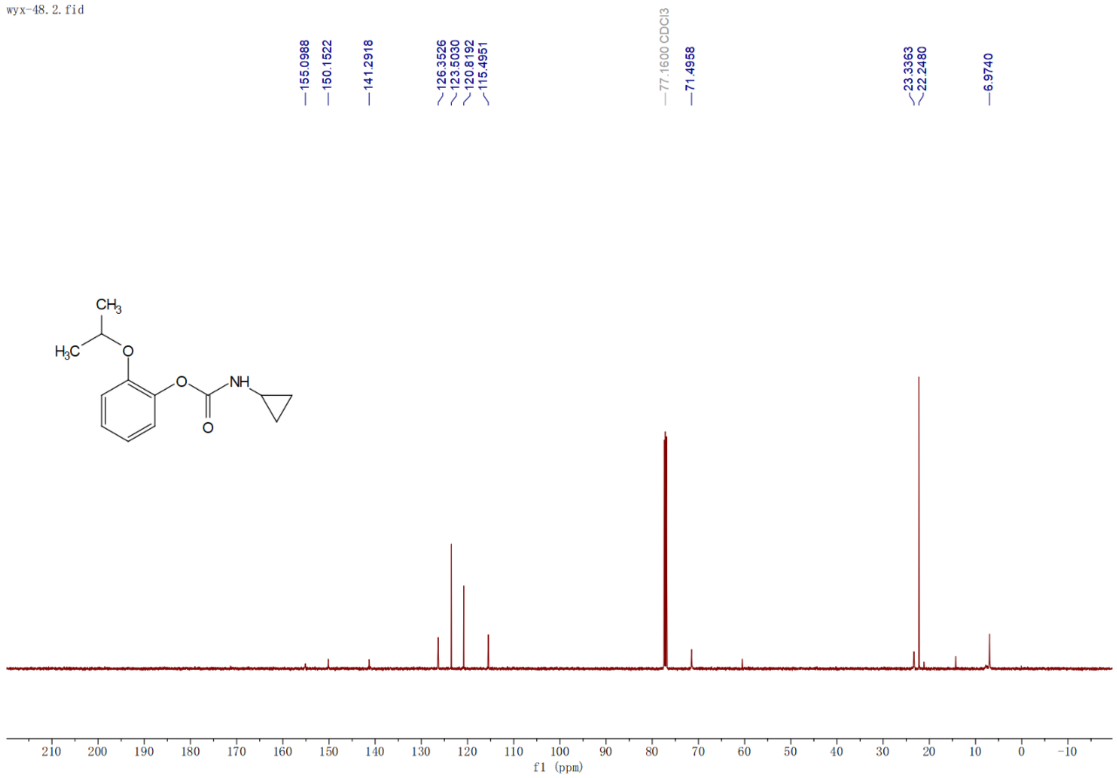

Figure S14. ^{1}H-NMR and ^{13}C-NMR spectra of compound **3b**.

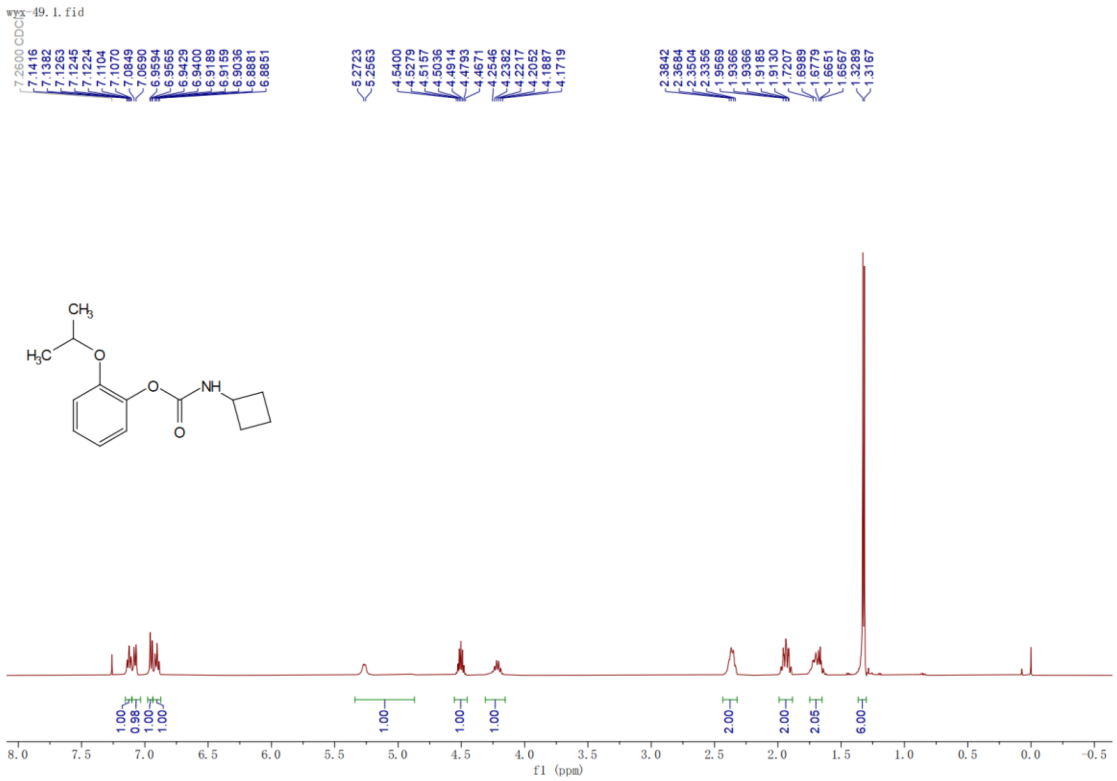

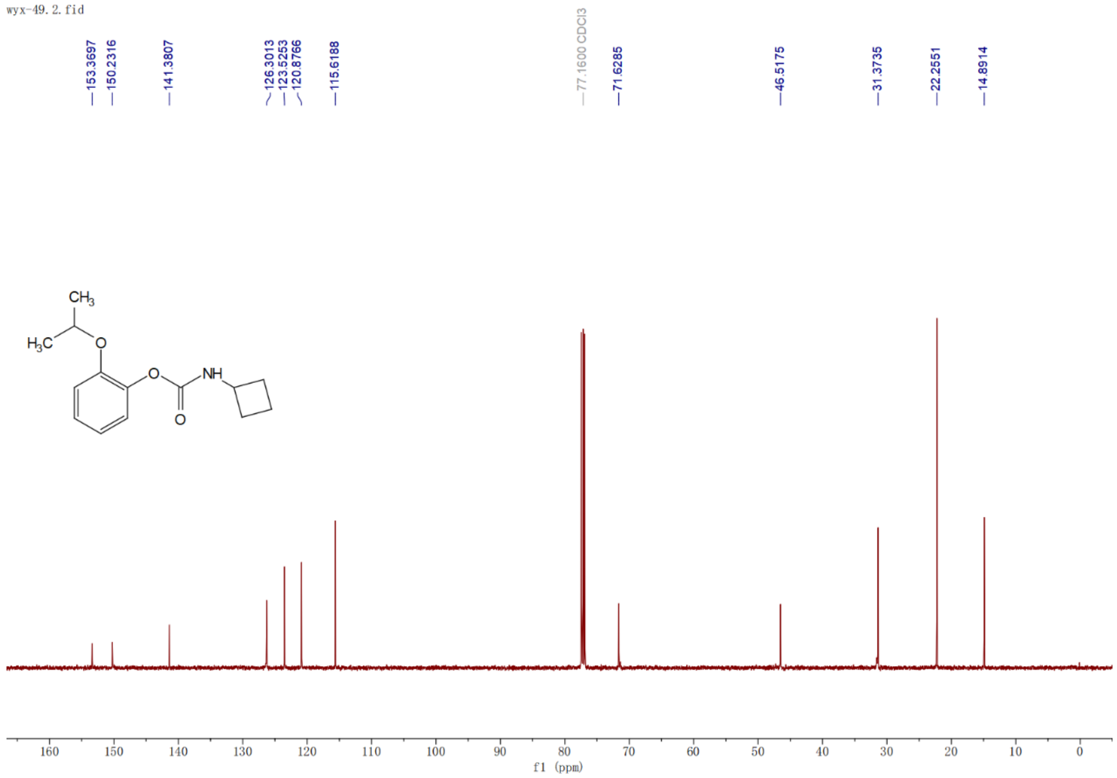

Figure S15. ^{1}H-NMR and ^{13}C-NMR spectra of compound **3c**.

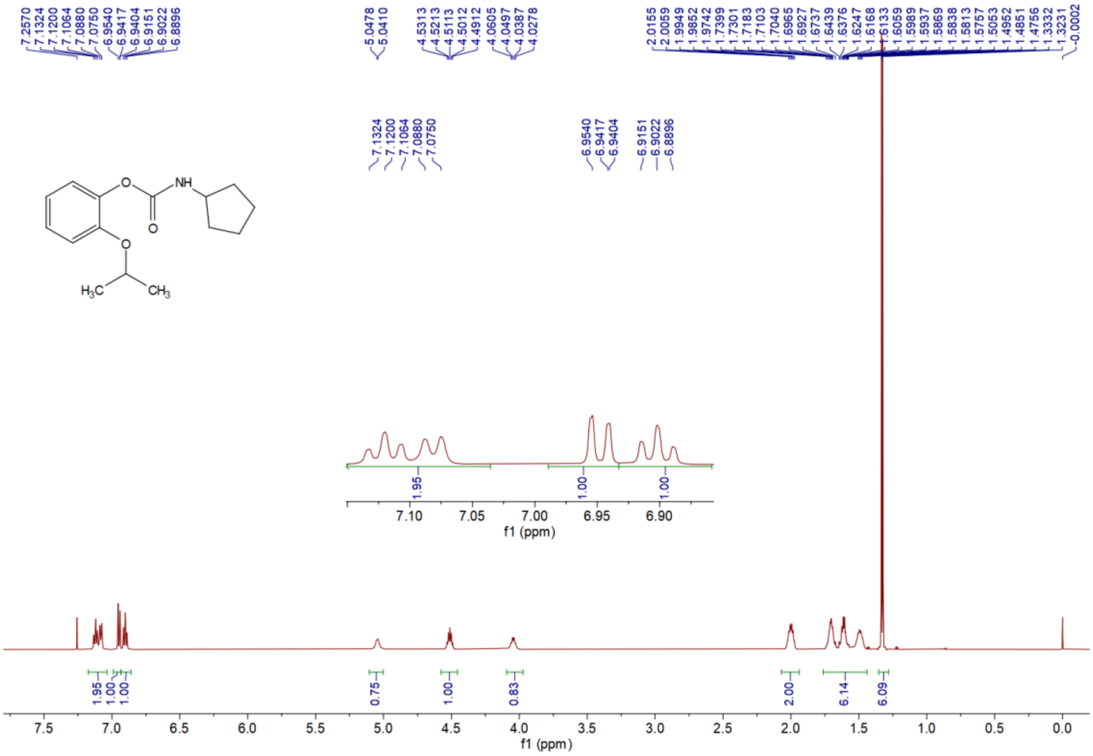

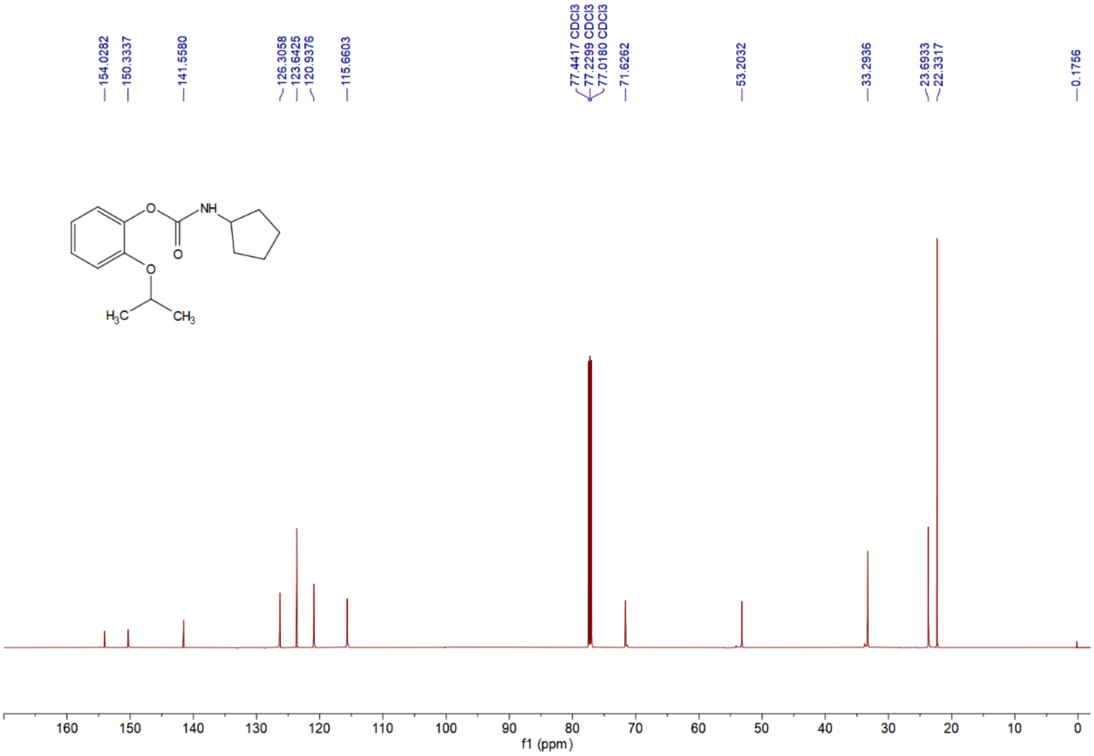

Figure S16. ^{1}H-NMR and ^{13}C-NMR spectra of compound **3d**.

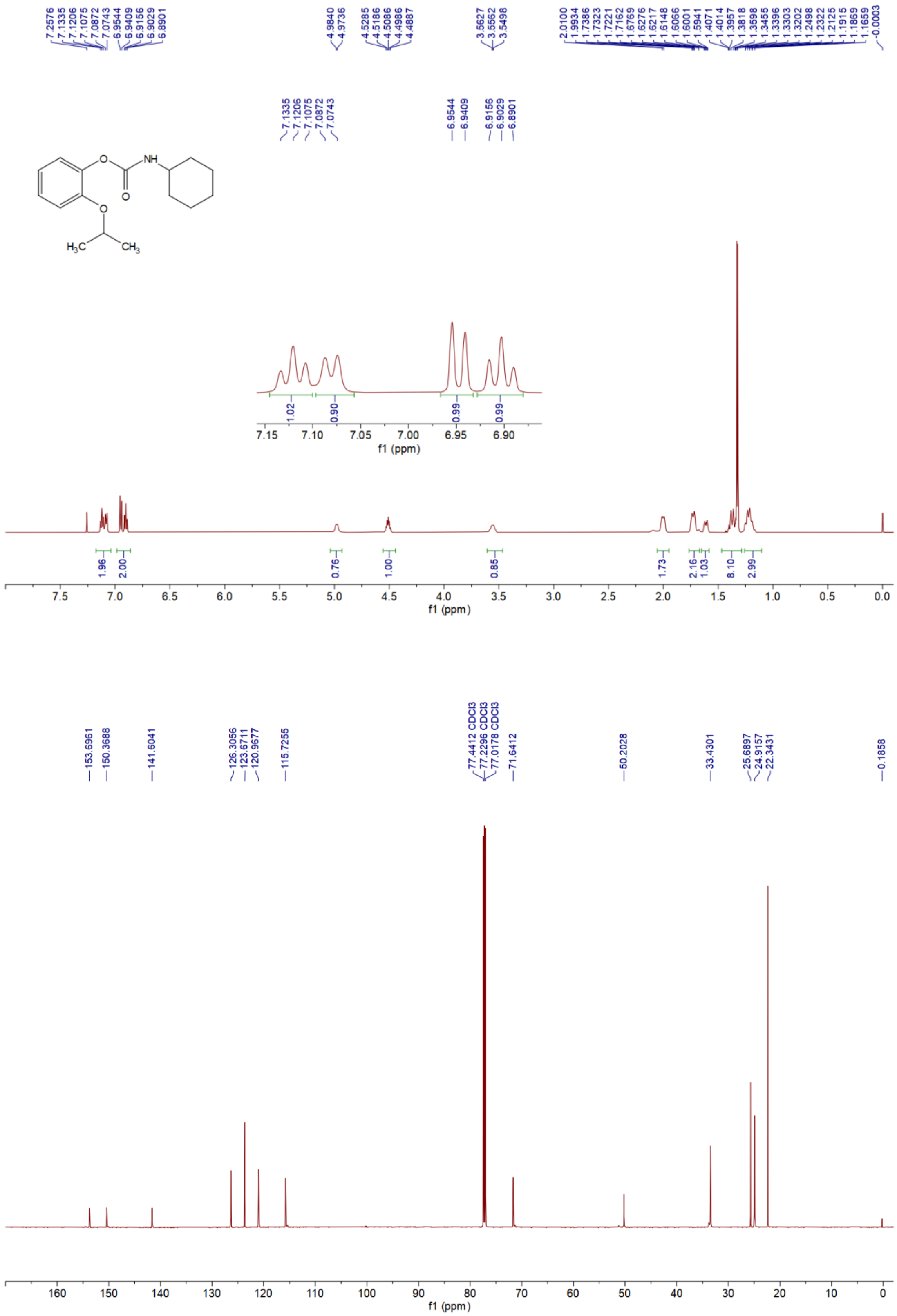

Figure S17. ^{1}H-NMR and ^{13}C-NMR spectra of compound **3e**.

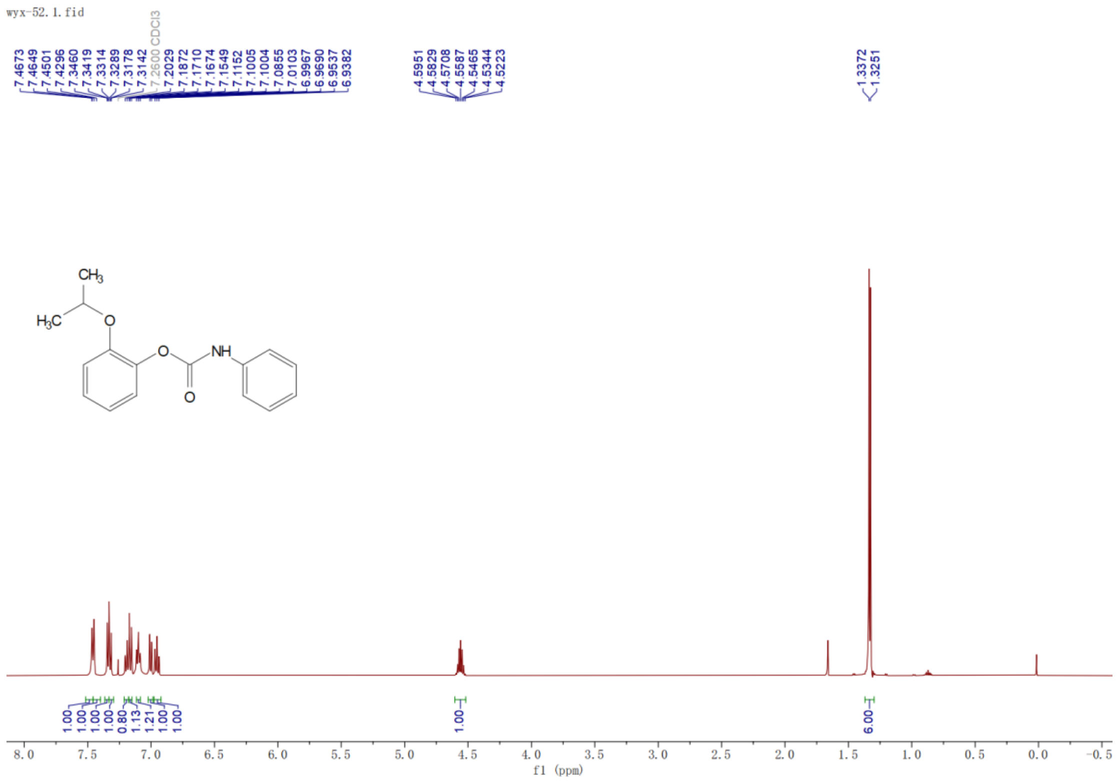

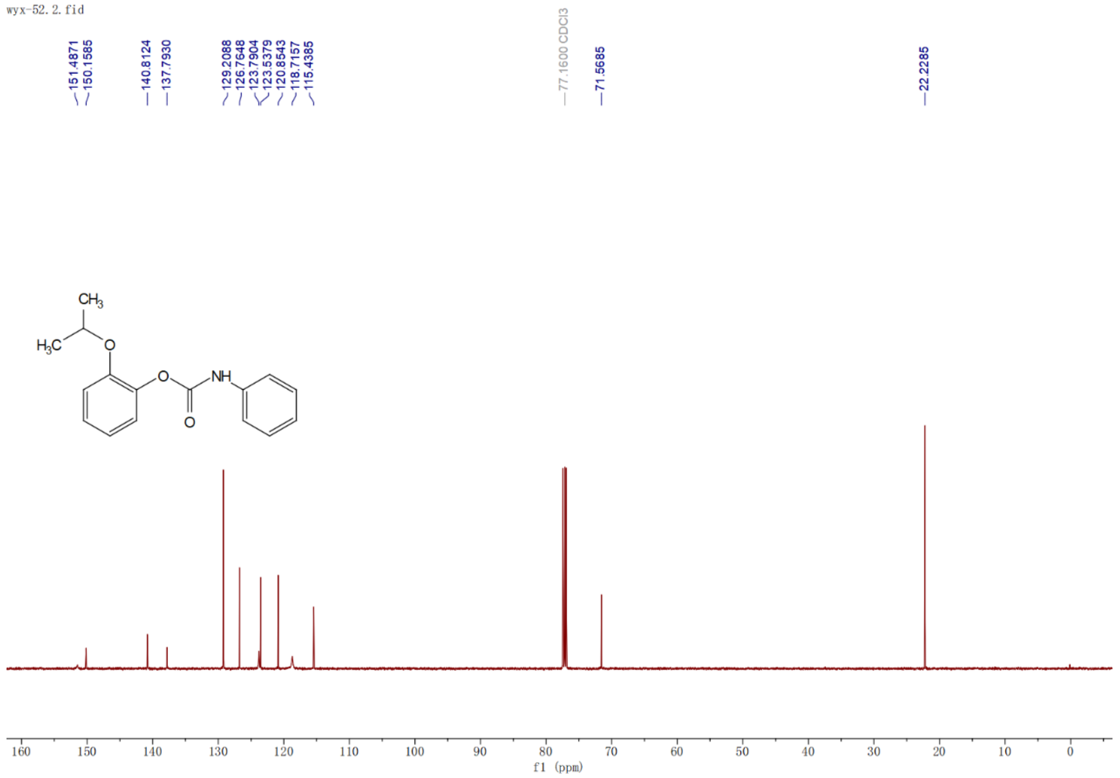

Figure S18. ^{1}H-NMR and ^{13}C-NMR spectra of compound **3f**.

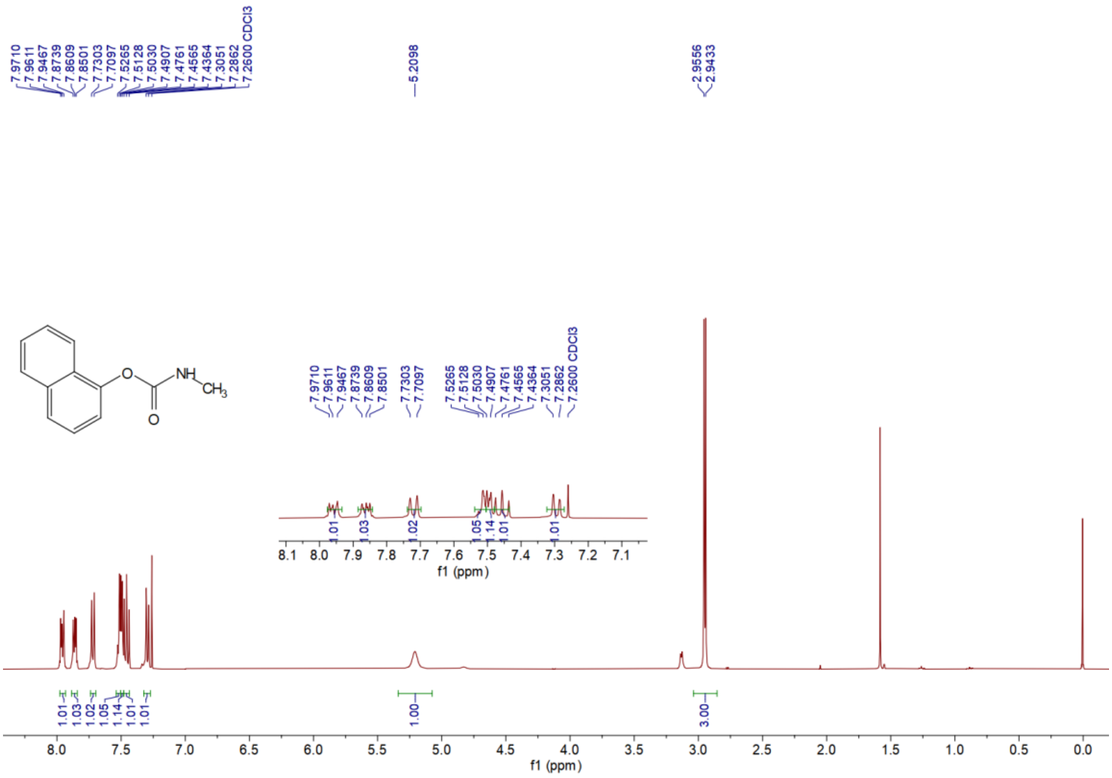

Figure S19. ^{1}H-NMR spectra of compound **4a**.

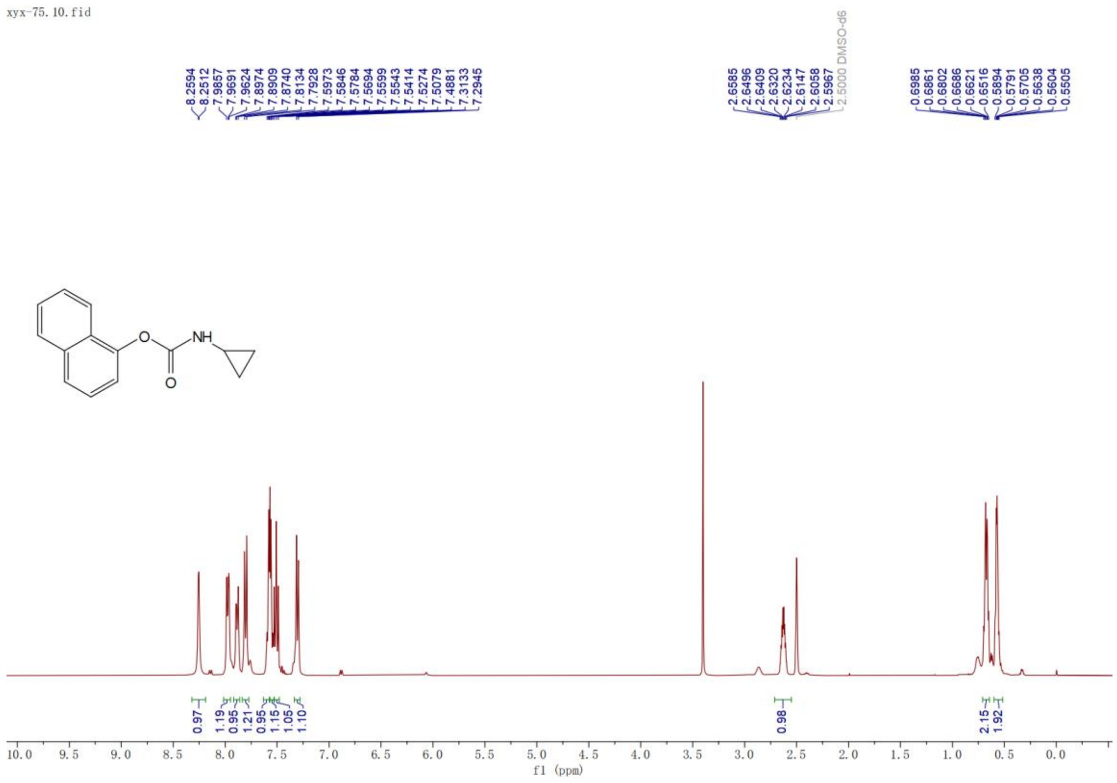

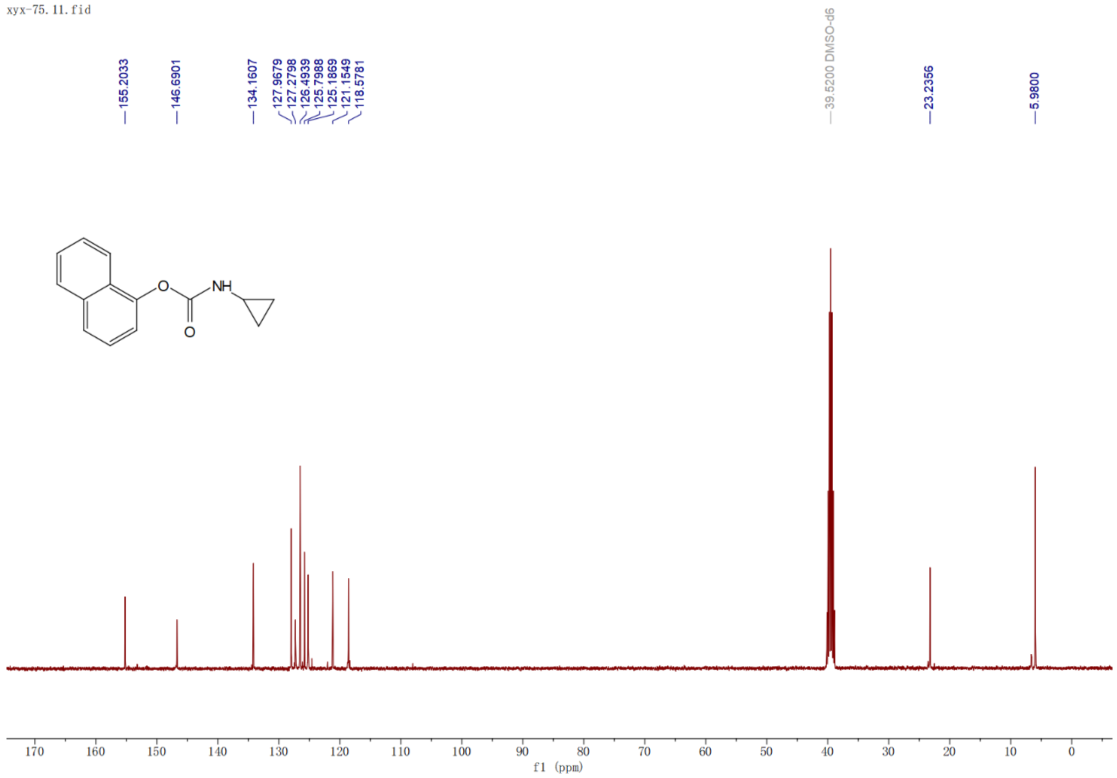

Figure S20. ^{1}H-NMR and ^{13}C-NMR spectra of compound **4b**.

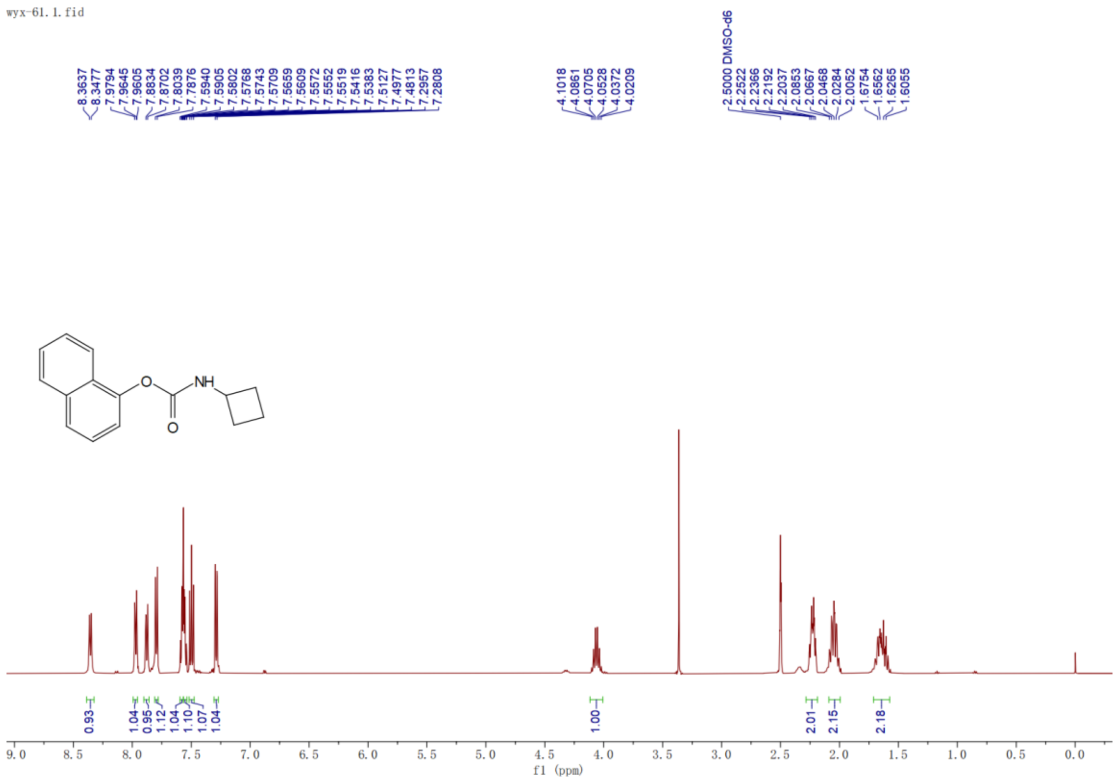

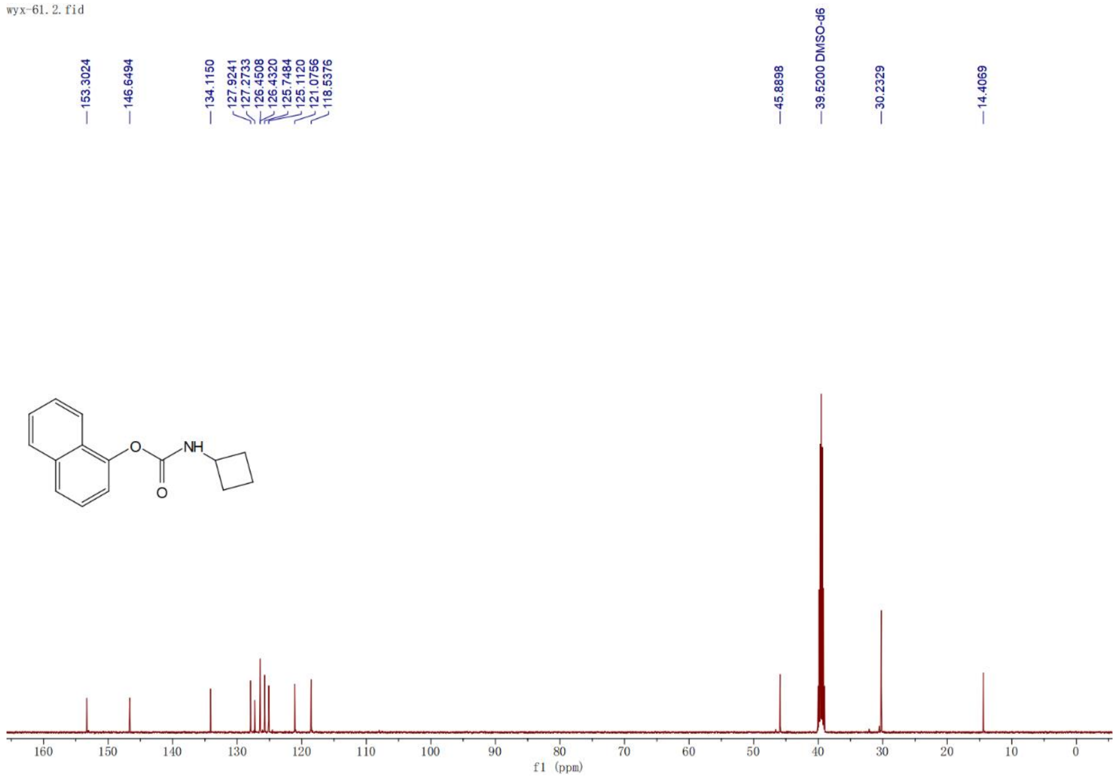

Figure S21. ^{1}H-NMR and ^{13}C-NMR spectra of compound **4c**.

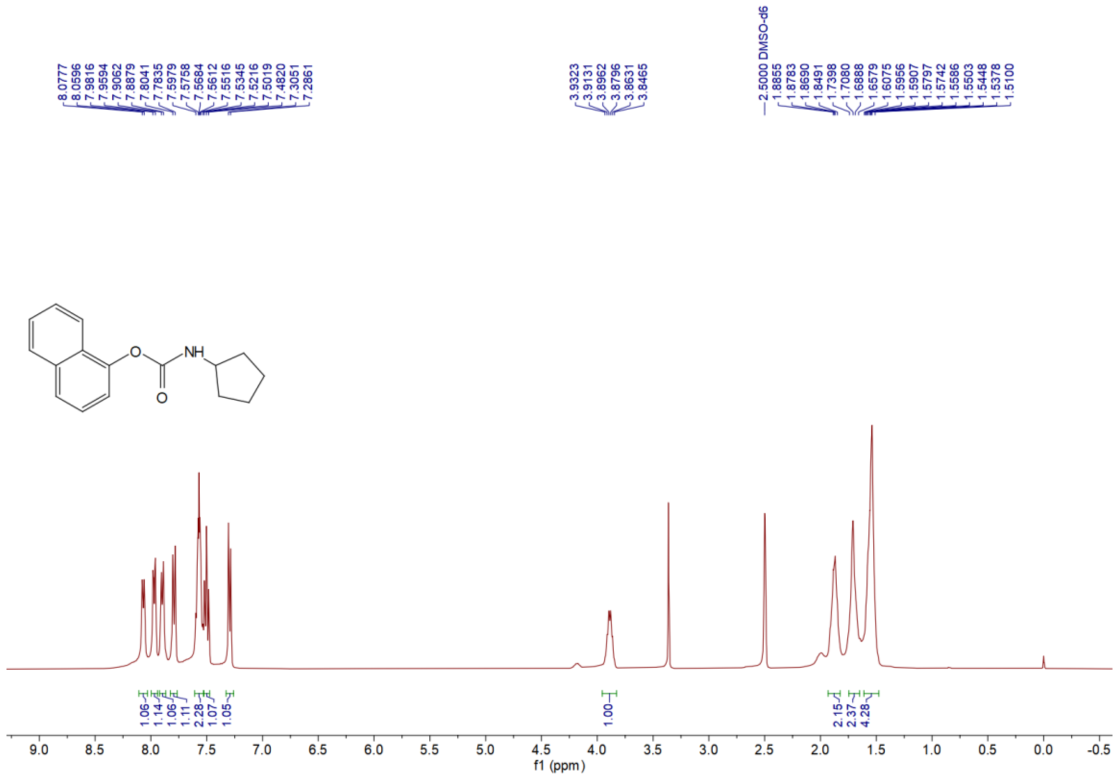

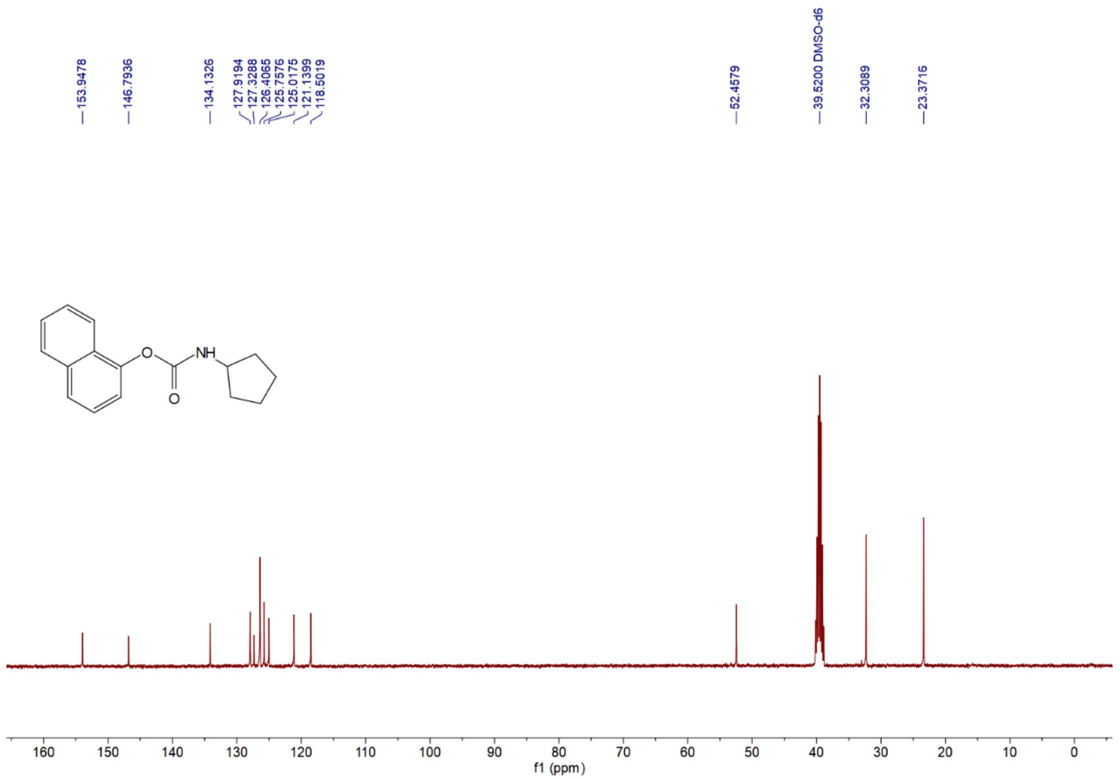

Figure S22. ^{1}H-NMR and ^{13}C-NMR spectra of compound **4d**.

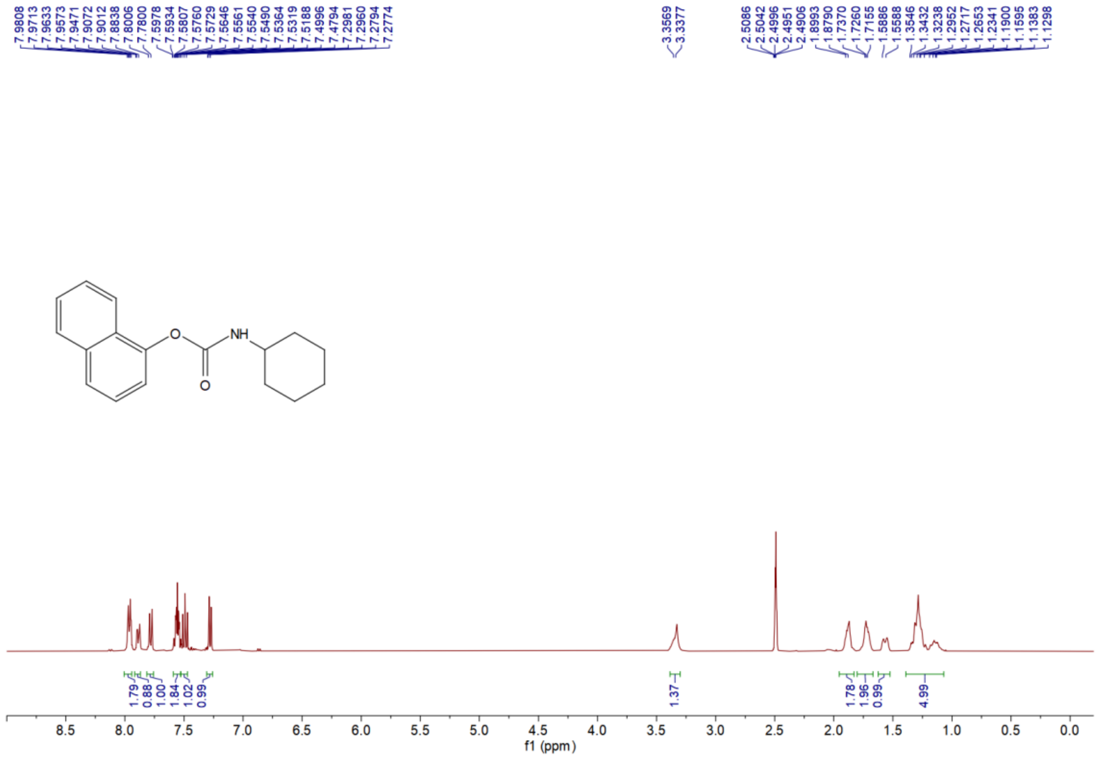

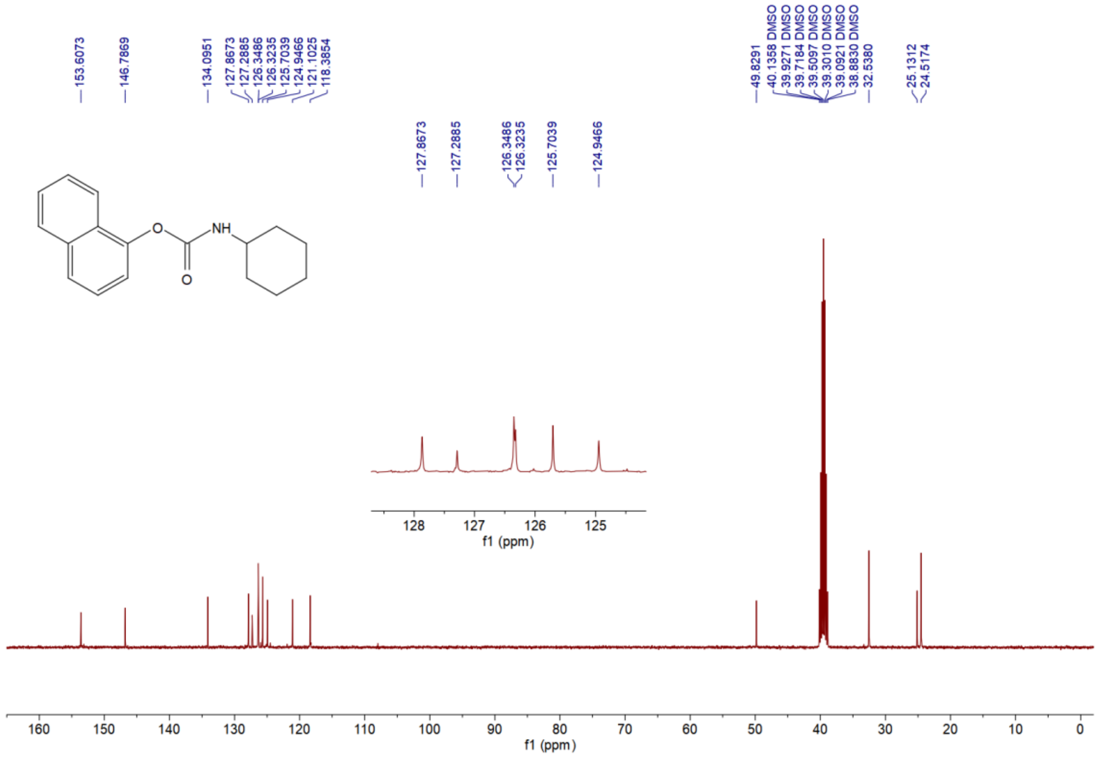

Figure S23. ^{1}H-NMR and ^{13}C-NMR spectra of compound **4e**.

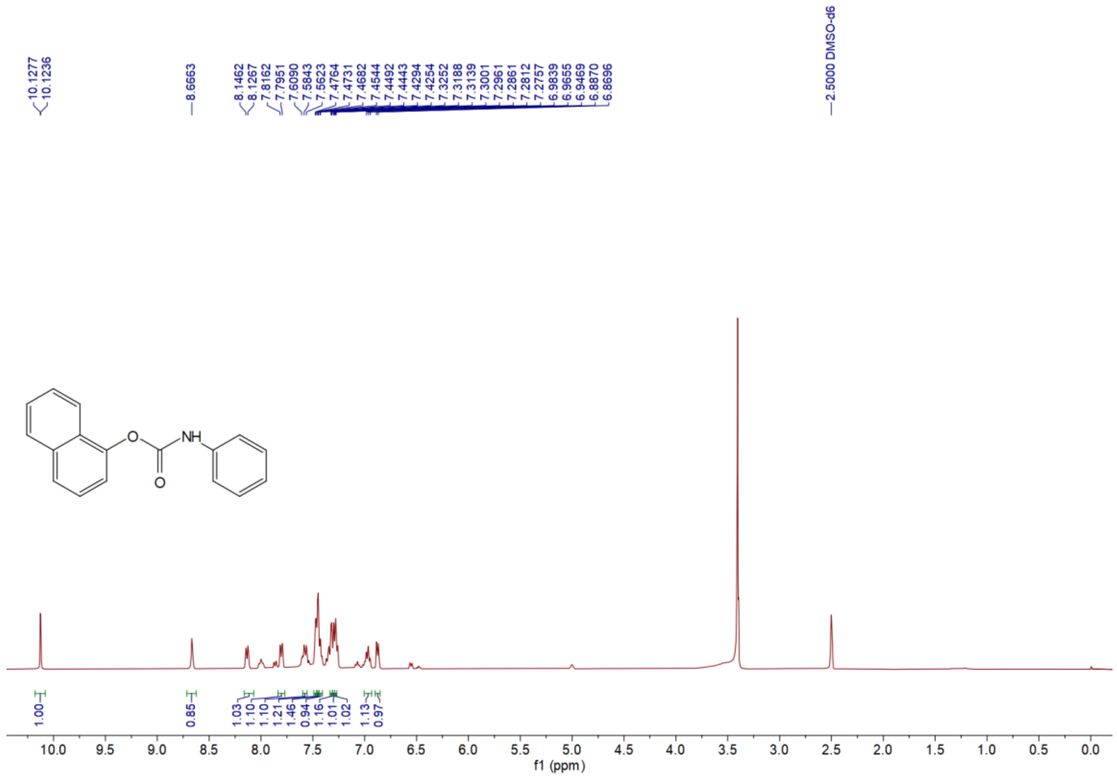

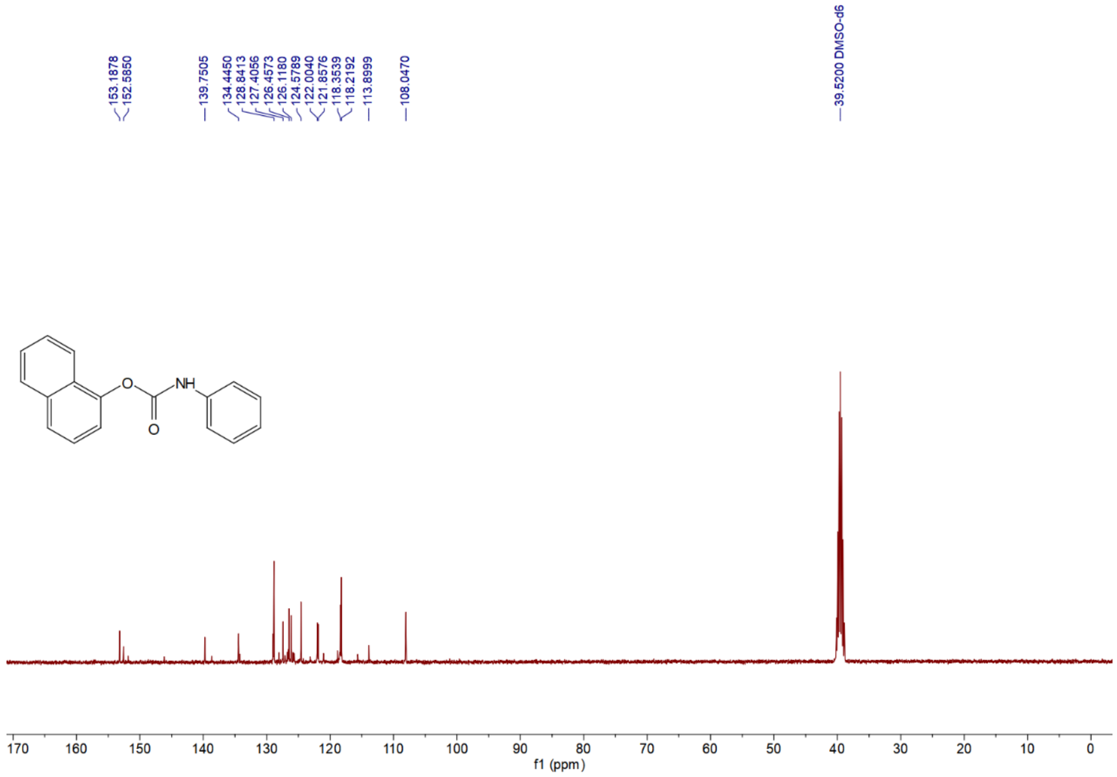

Figure S24. ^{1}H-NMR and ^{13}C-NMR spectra of compound **4f**.

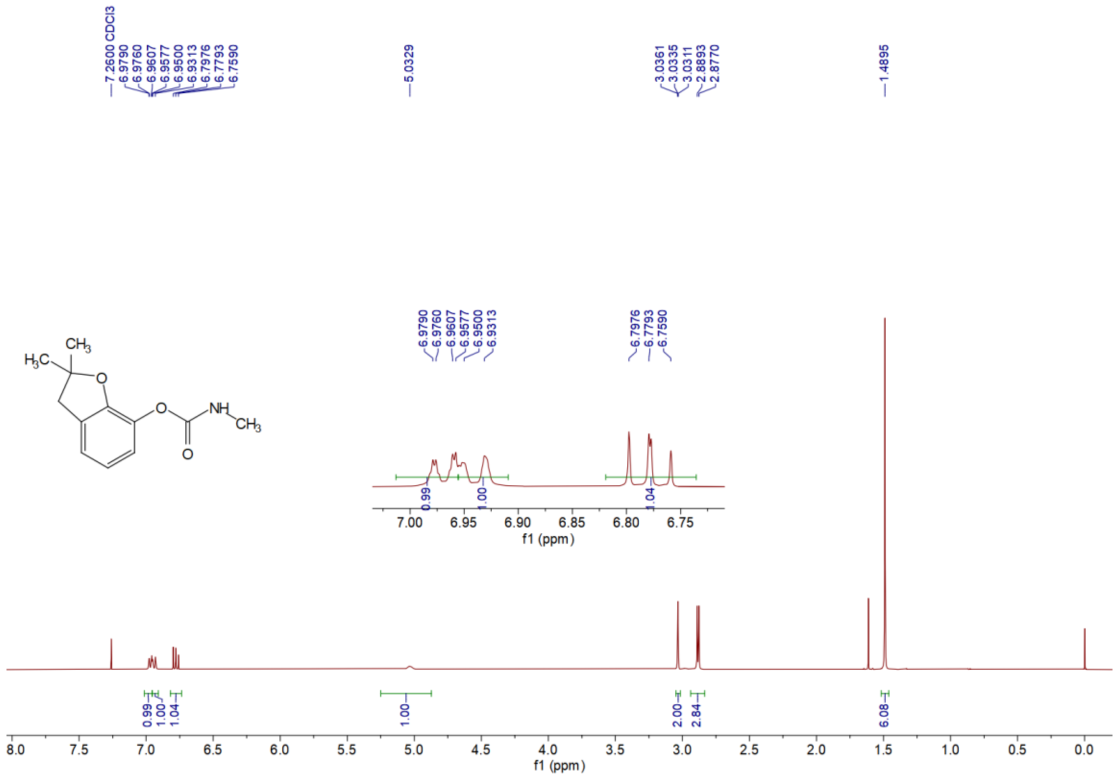

Figure S25. ^{1}H-NMR spectra of compound **5a**.

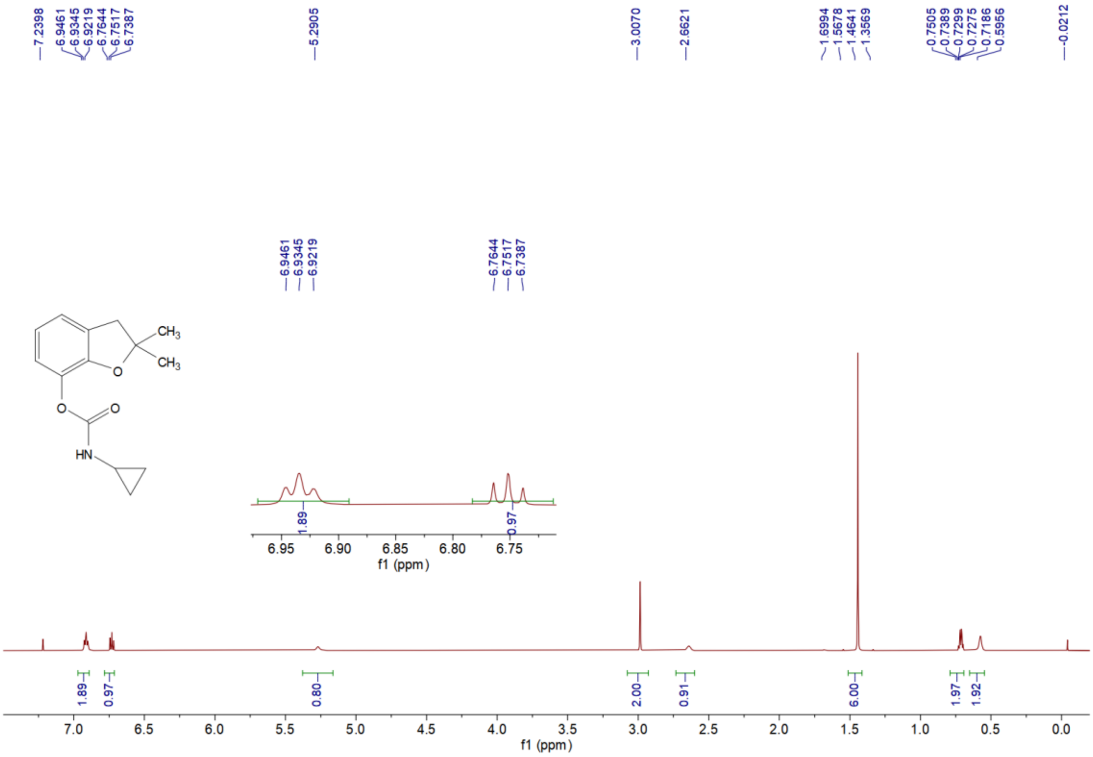

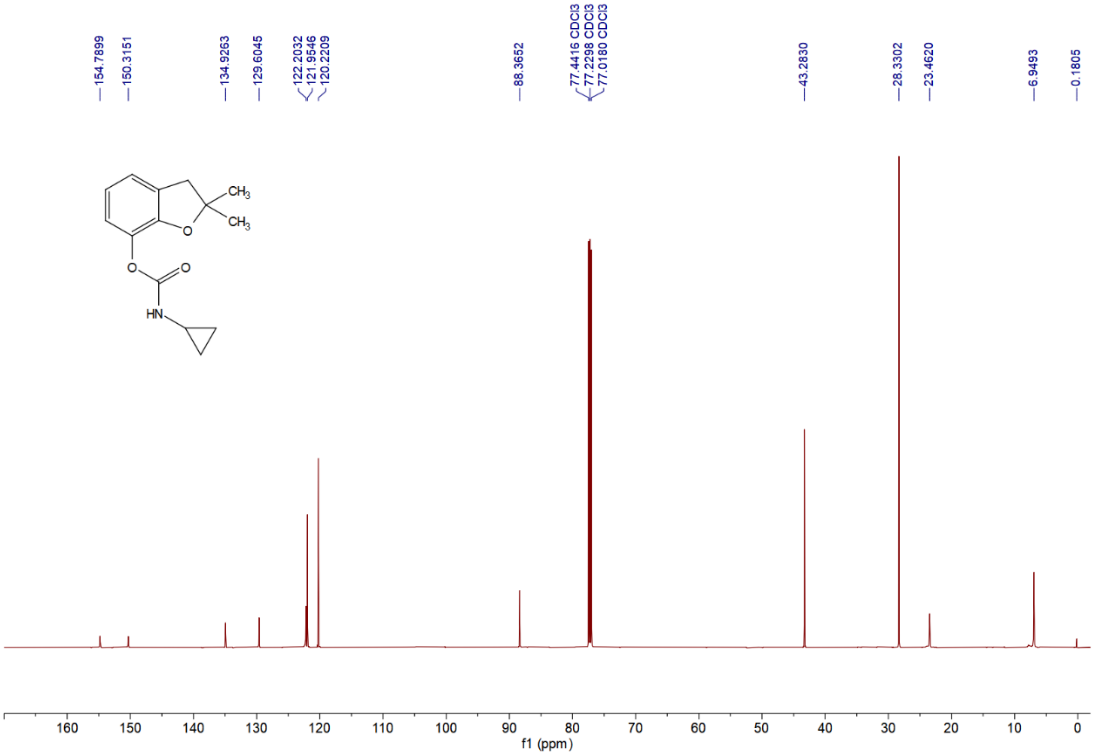

Figure S26. ^{1}H-NMR and ^{13}C-NMR spectra of compound **5b**.

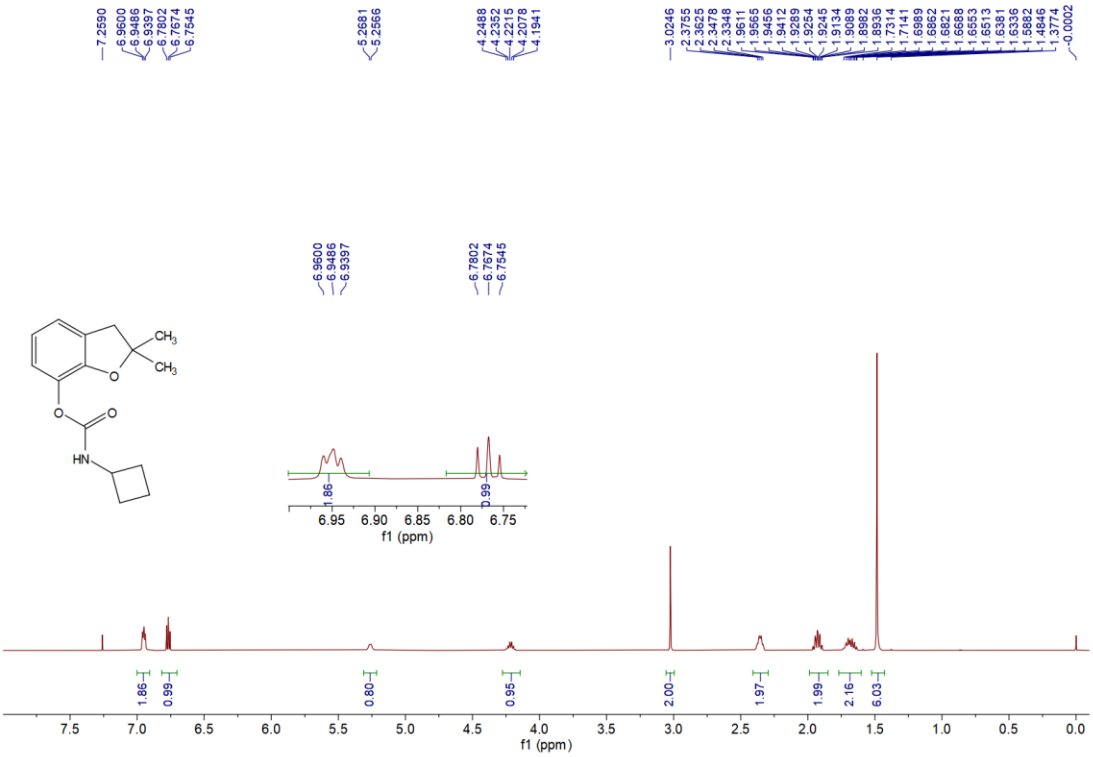

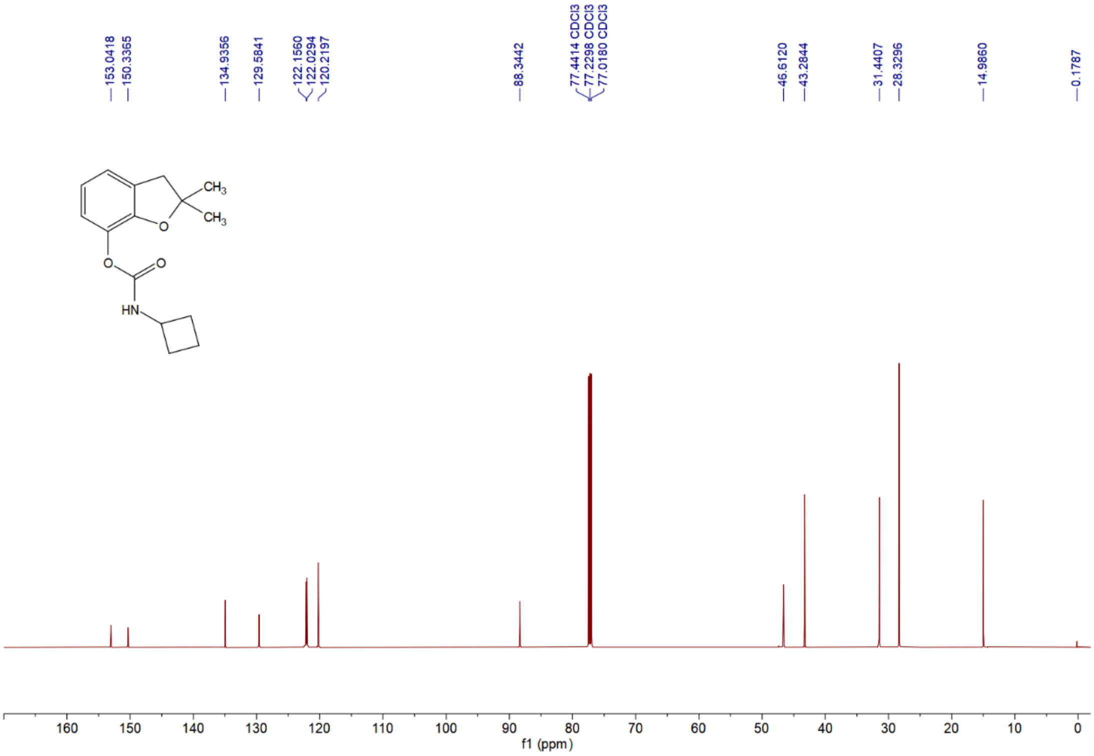

Figure S27. ^{1}H-NMR and ^{13}C-NMR spectra of compound **5c**.

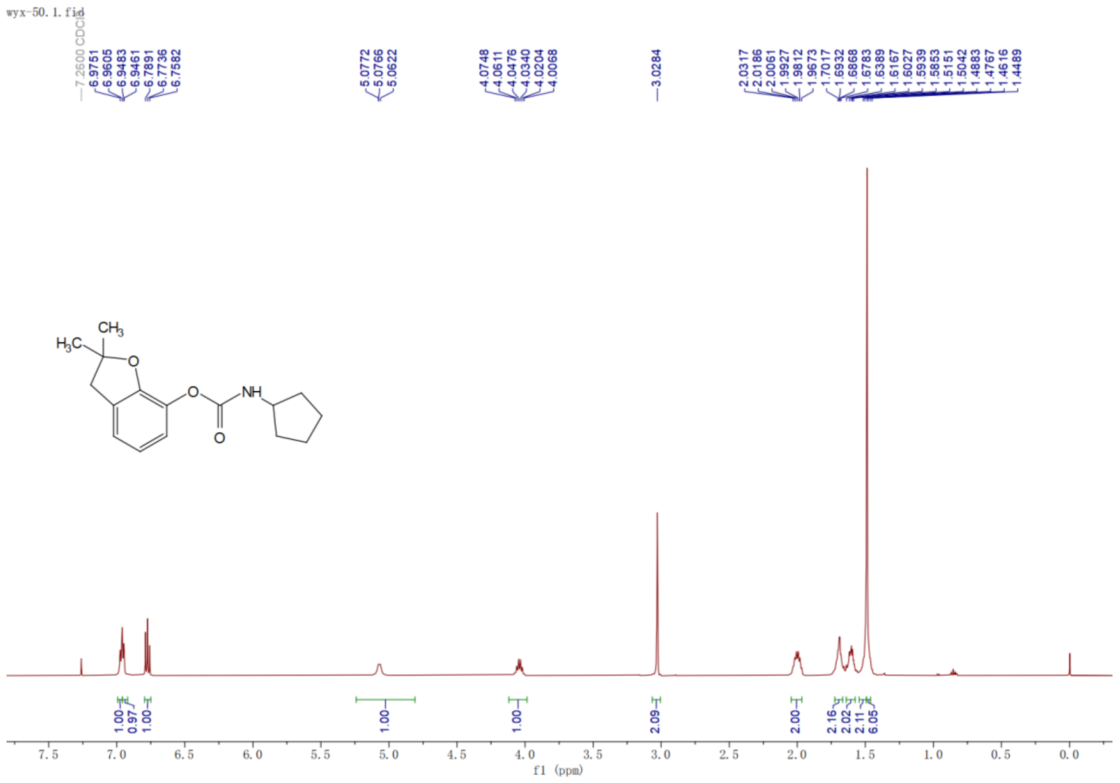

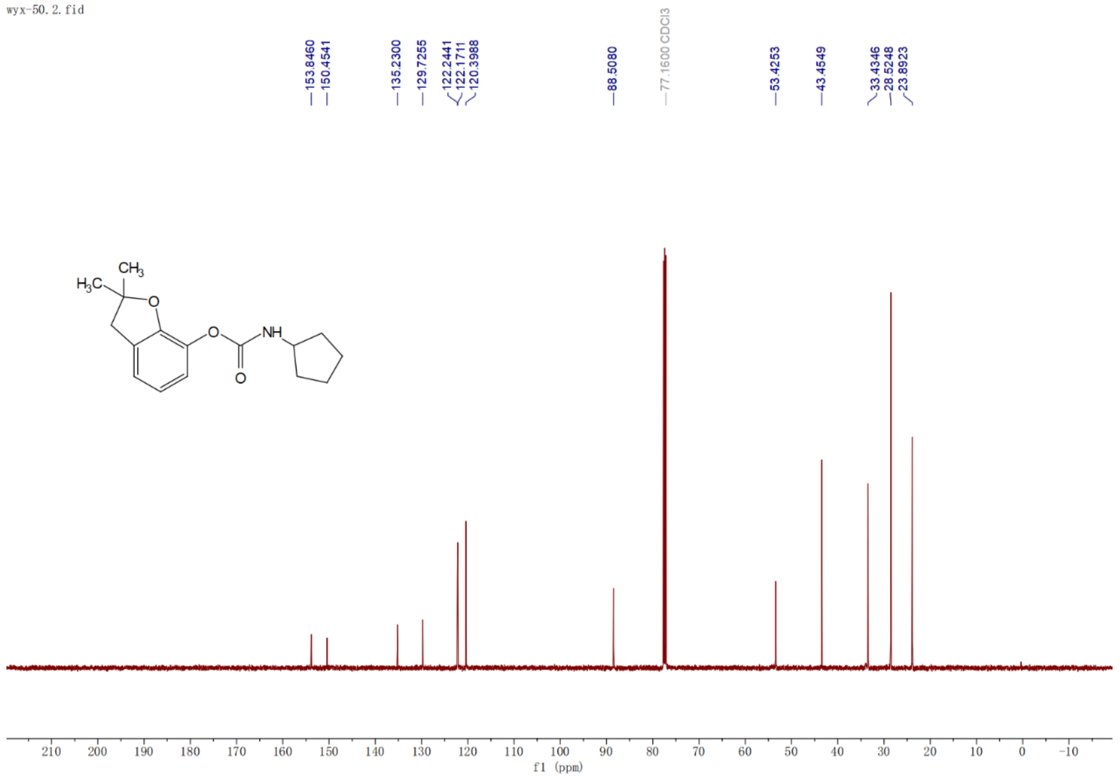

Figure S28. ^{1}H-NMR and ^{13}C-NMR spectra of compound **5d.**

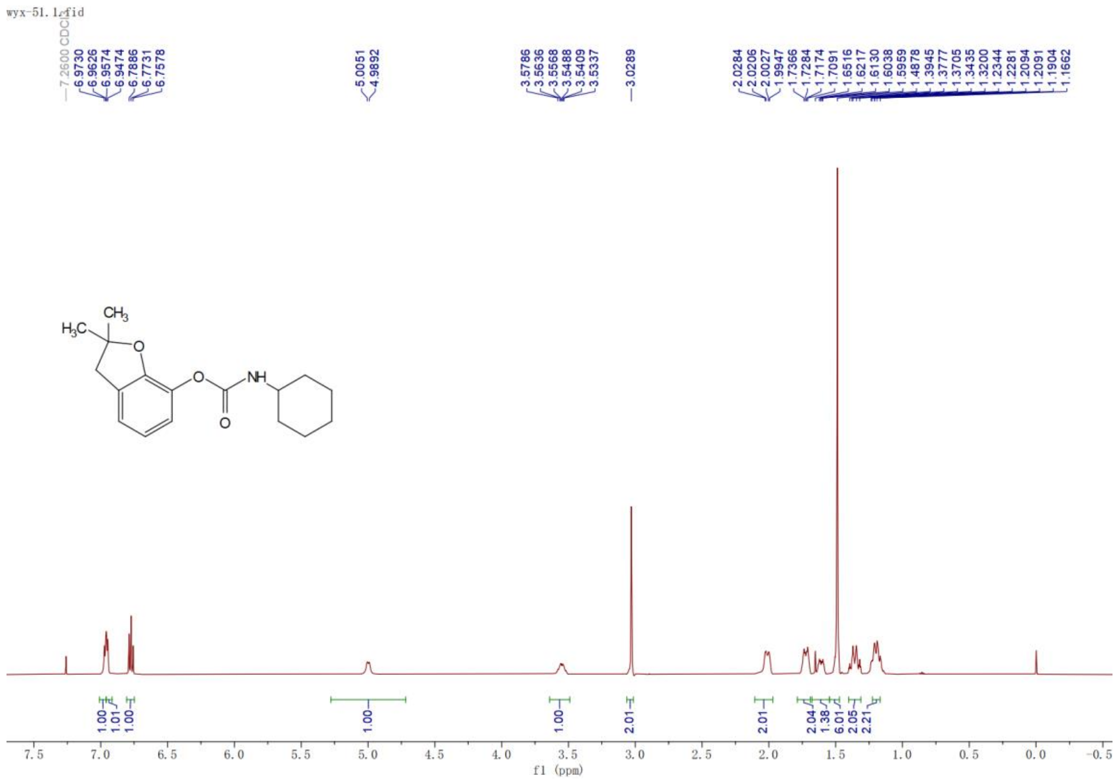

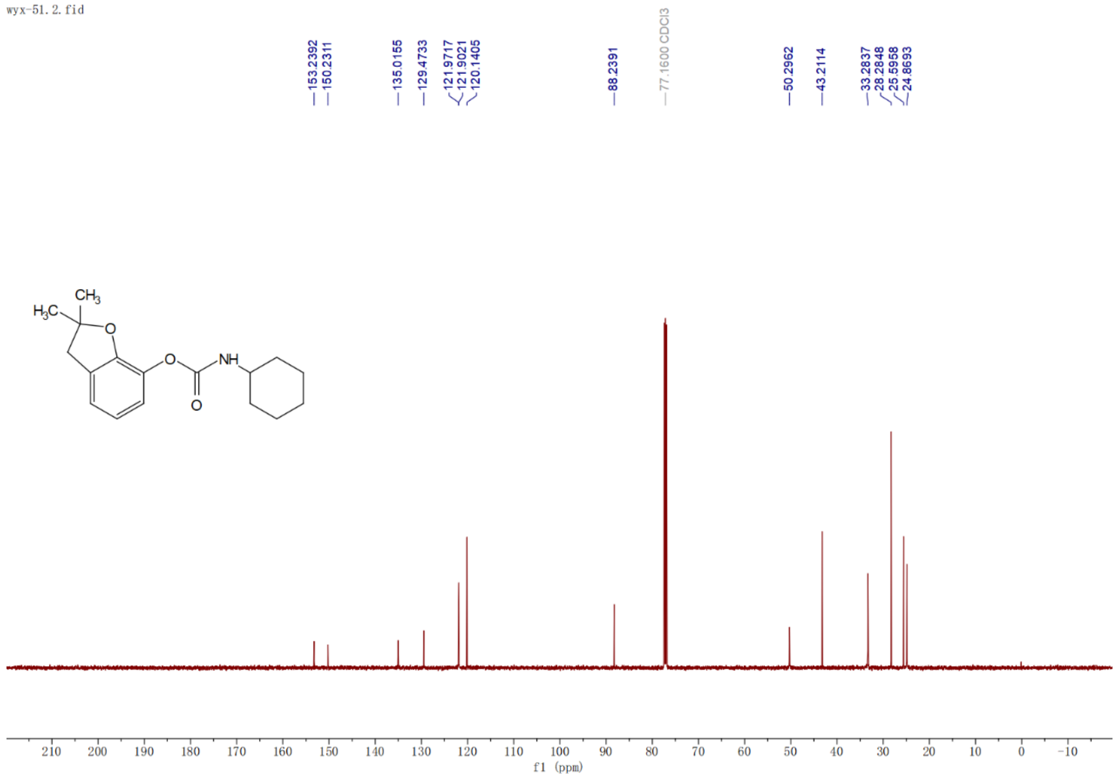

Figure S29. ^{1}H-NMR and ^{13}C-NMR spectra of compound **5e**.

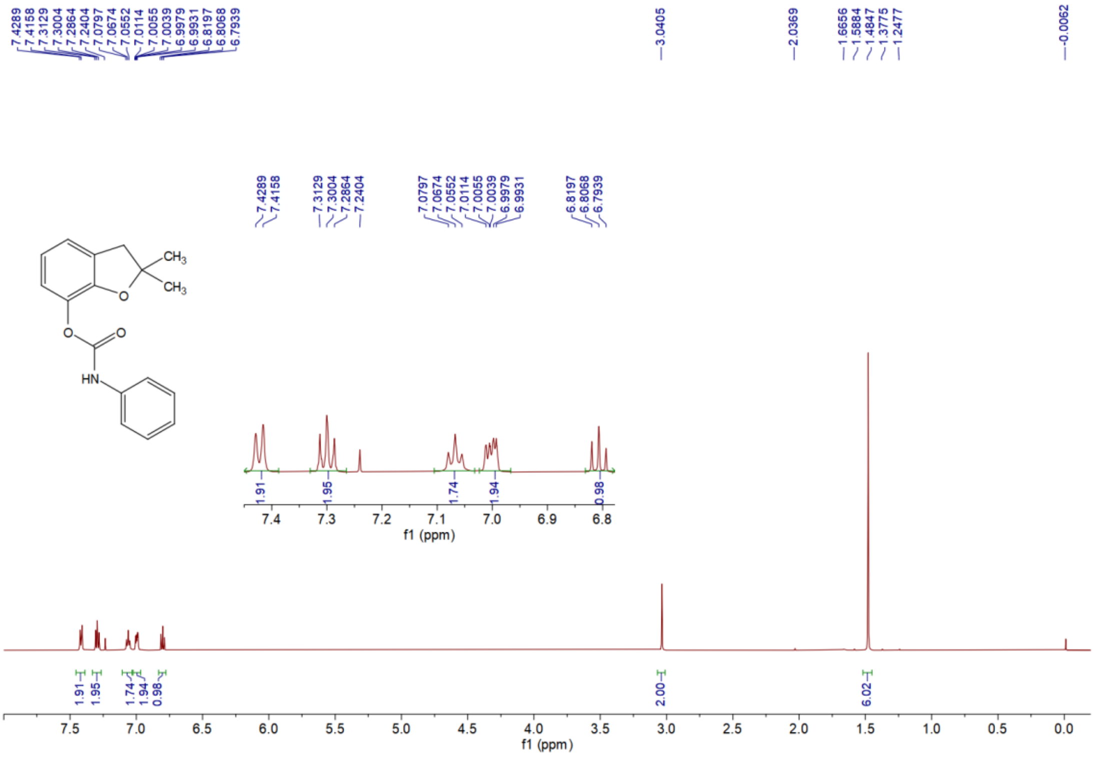

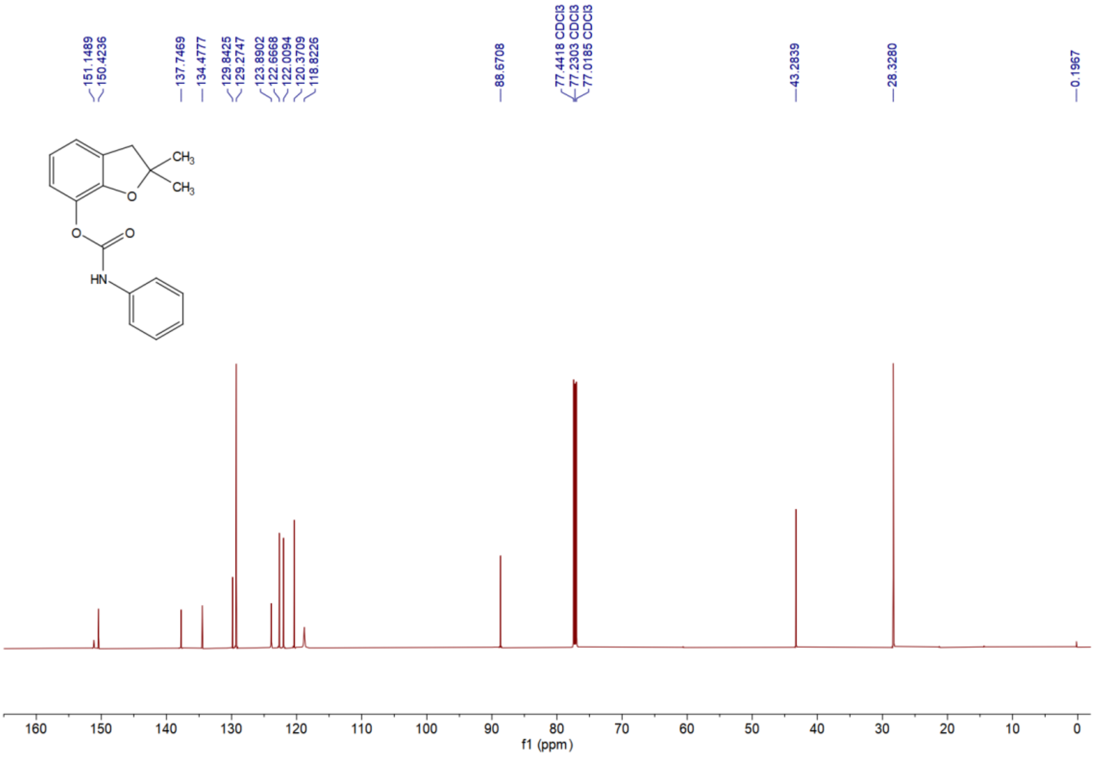

Figure S30. ^{1}H-NMR and ^{13}C-NMR spectra of compound **5f**.

7. TOF-HRMS (ESI) spectra

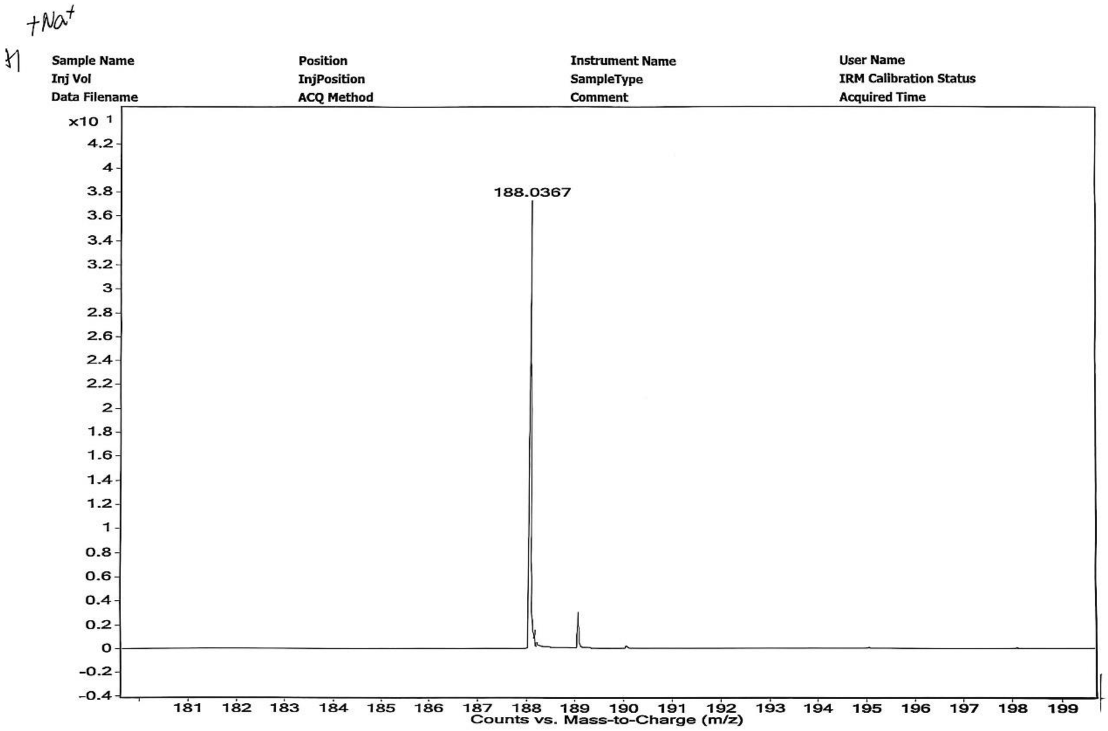

Figure S31. TOF-HRMS (ESI) spectra of compound **1a**.

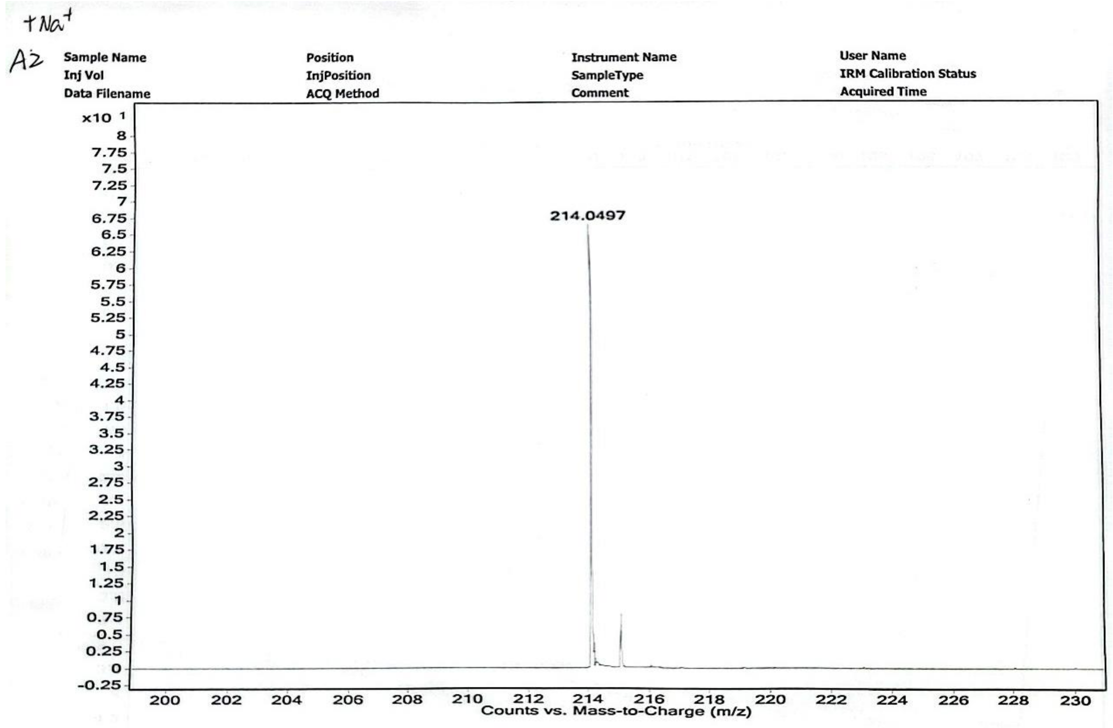

Figure S32. TOF-HRMS (ESI) spectra of compound **1b**.

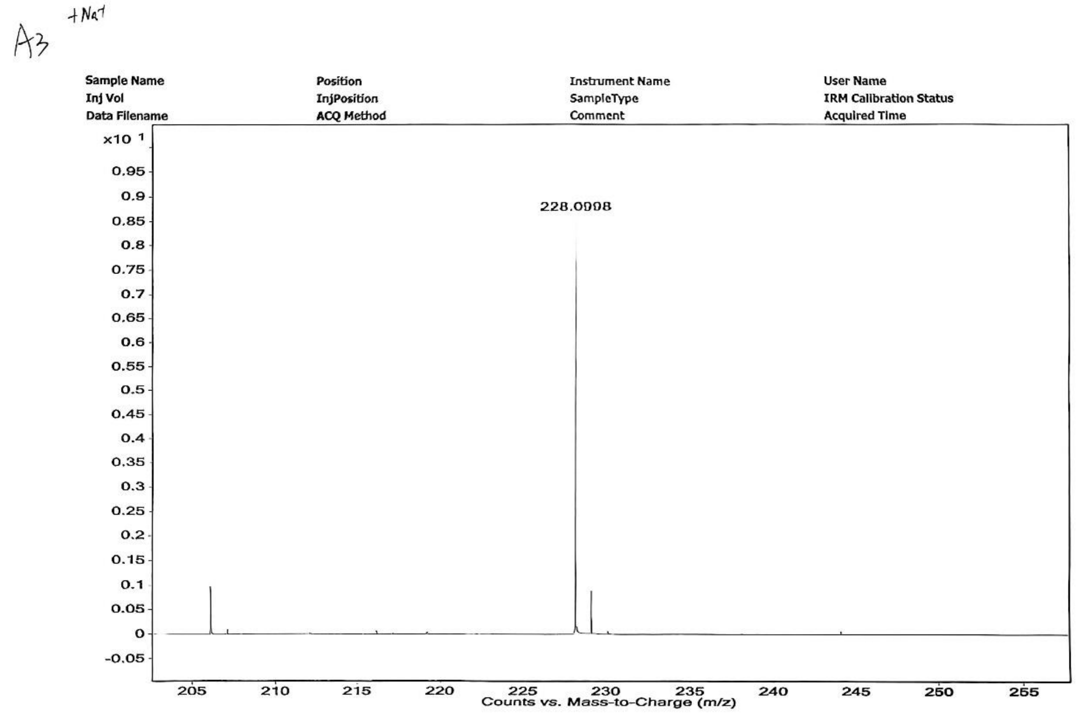

Figure S33. TOF-HRMS (ESI) spectra of compound **1c**.

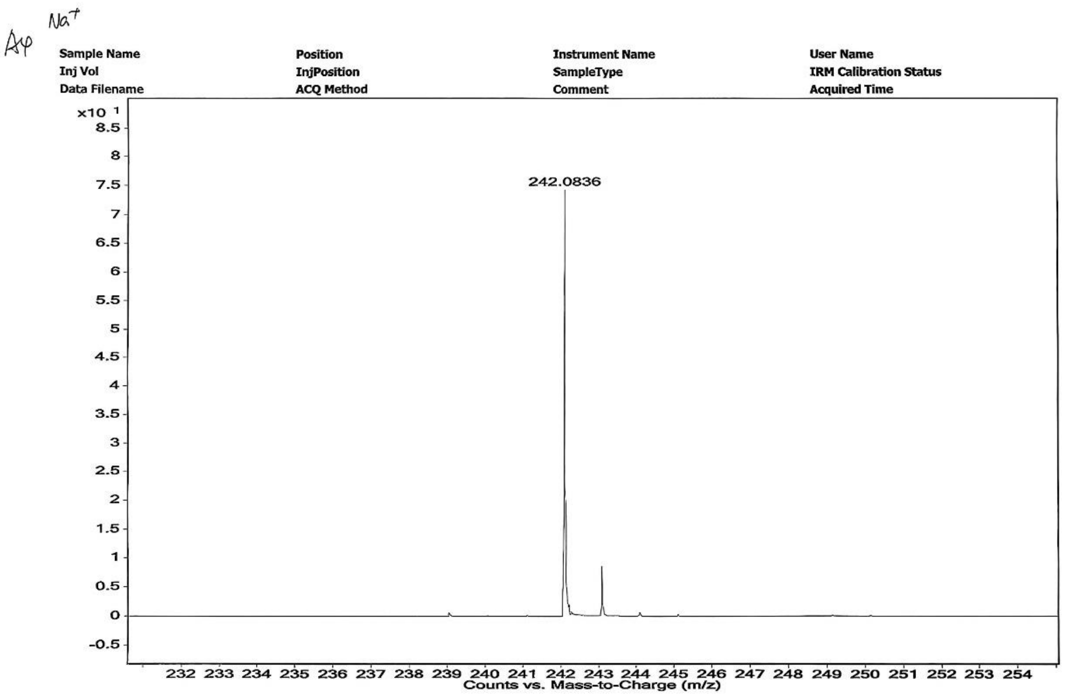

Figure S34. TOF-HRMS (ESI) spectra of compound **1d**.

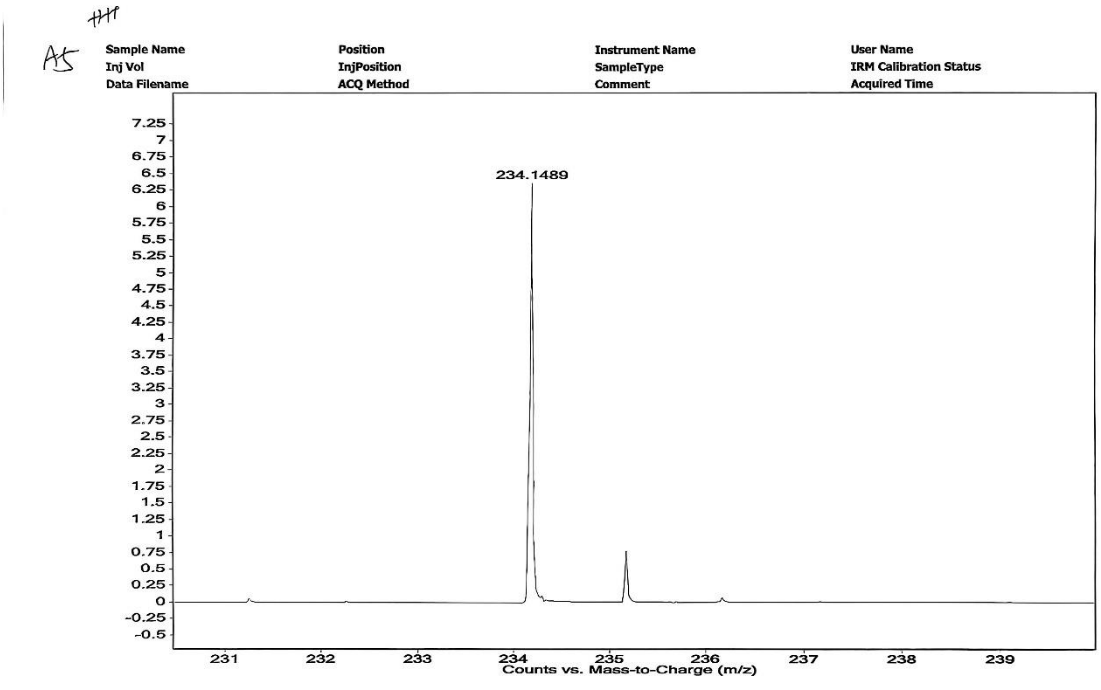

Figure S35. TOF-HRMS (ESI) spectra of compound **1e**.

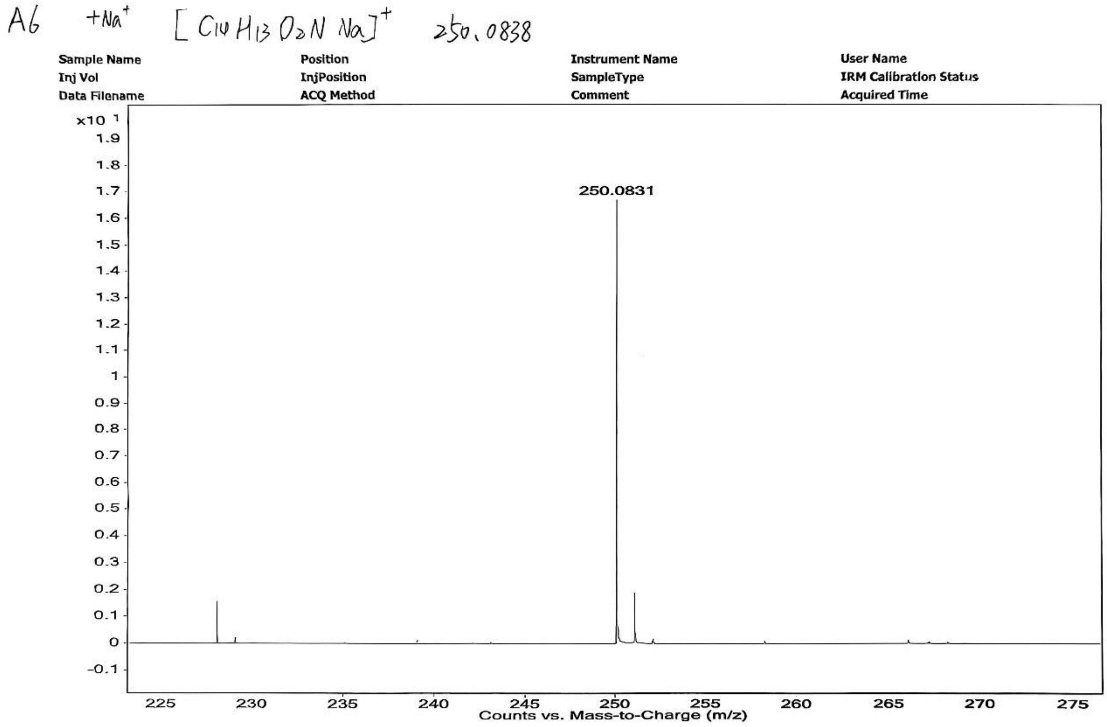

Figure S36. TOF-HRMS (ESI) spectra of compound **1f**.

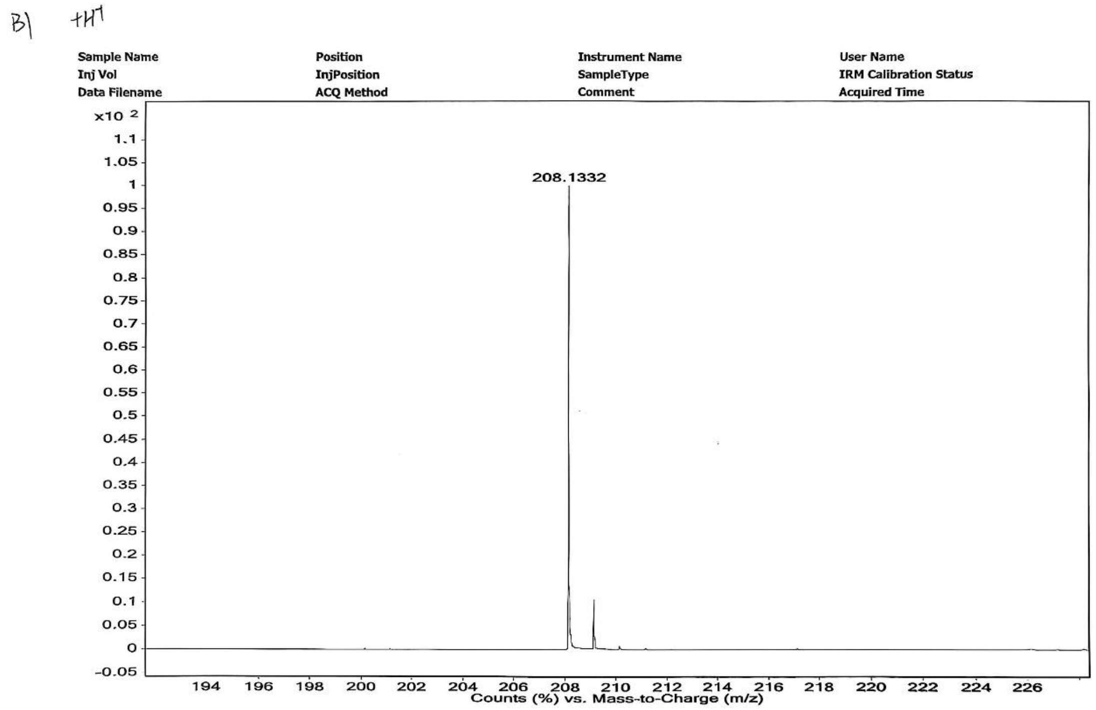

Figure S37. TOF-HRMS (ESI) spectra of compound **2a**.

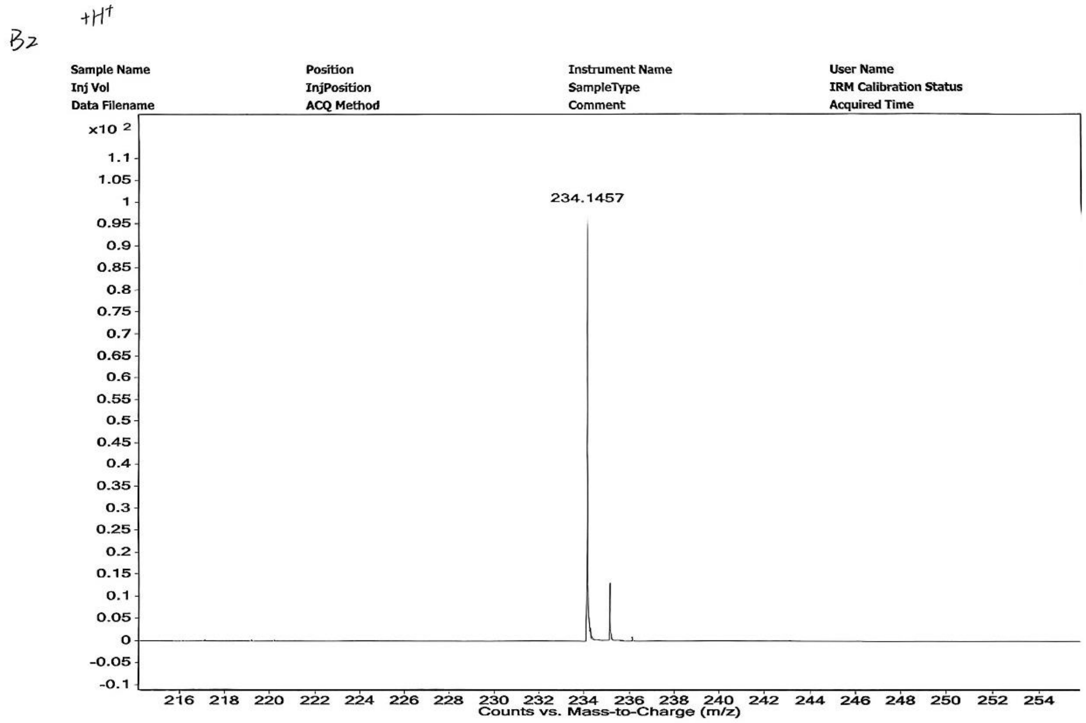

Figure S38. TOF-HRMS (ESI) spectra of compound **2b**.

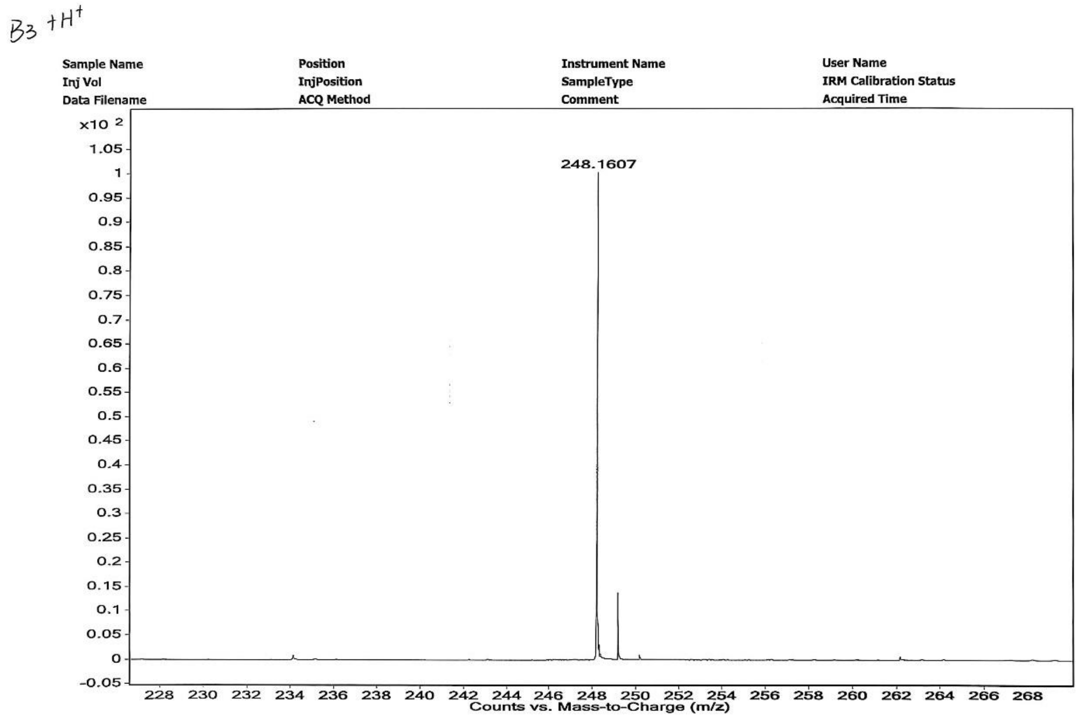

Figure S39. TOF-HRMS (ESI) spectra of compound **2c**.

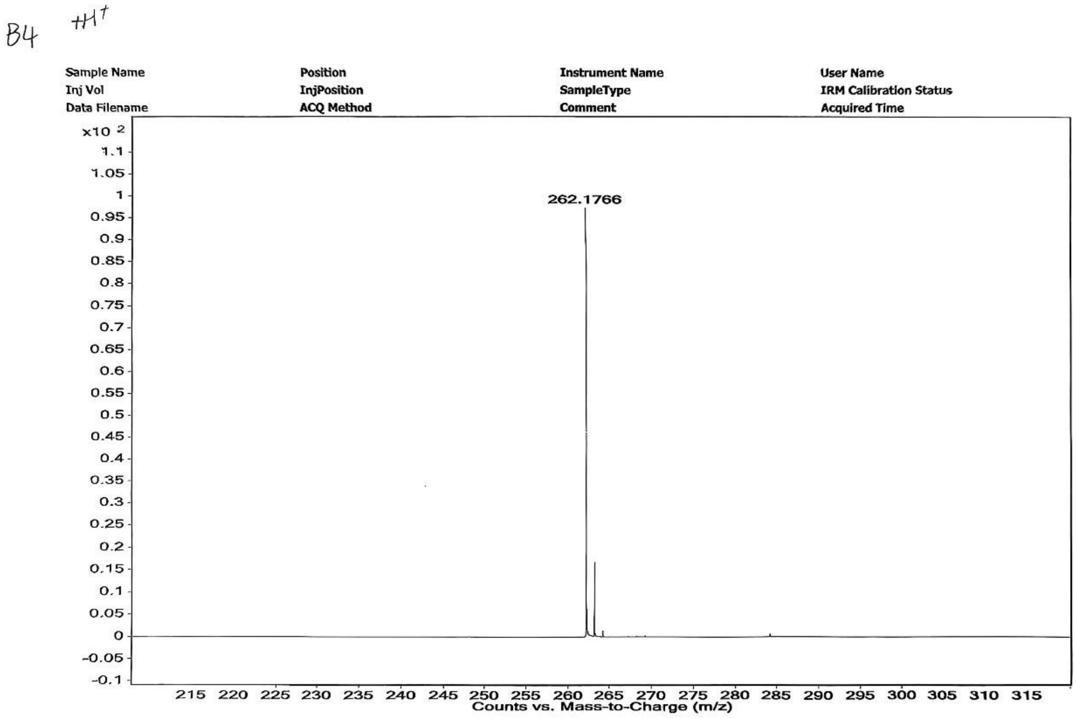

Figure S40. TOF-HRMS (ESI) spectra of compound **2d**.

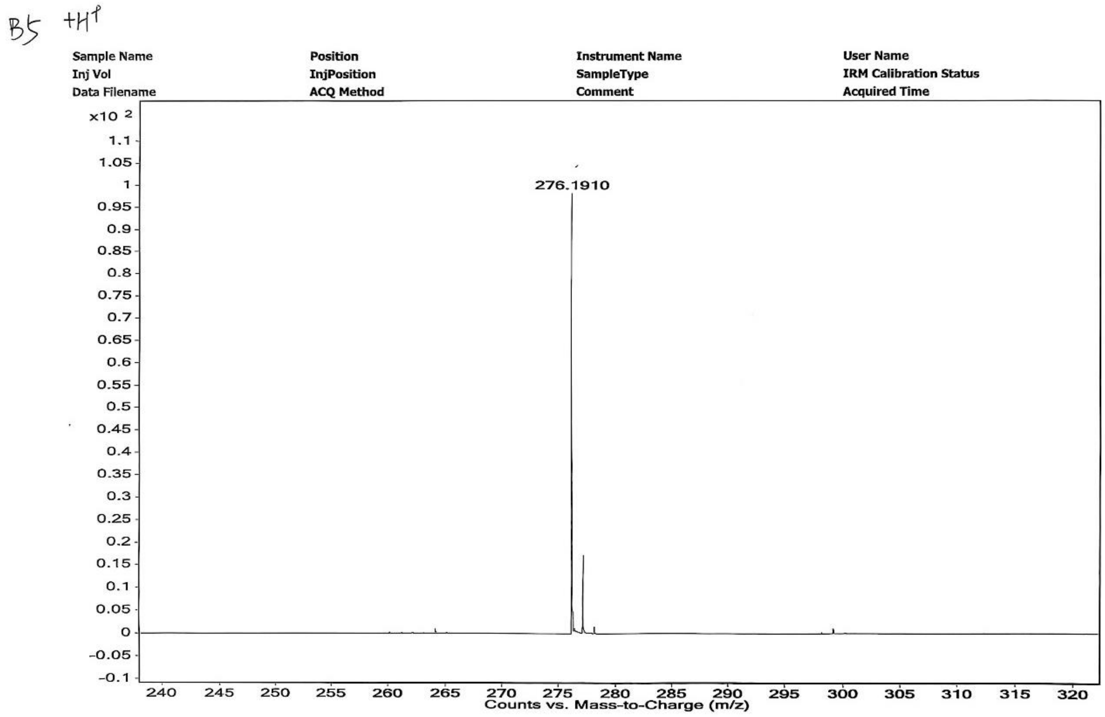

Figure S41. TOF-HRMS (ESI) spectra of compound **2e**.

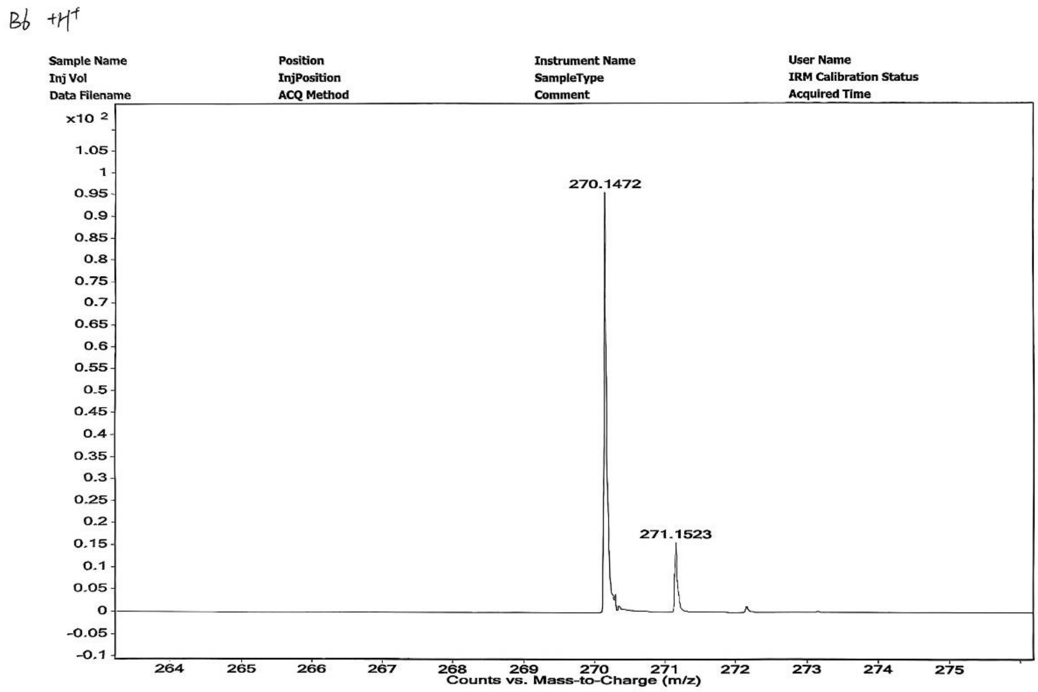

Figure S42. TOF-HRMS (ESI) spectra of compound **2f**.

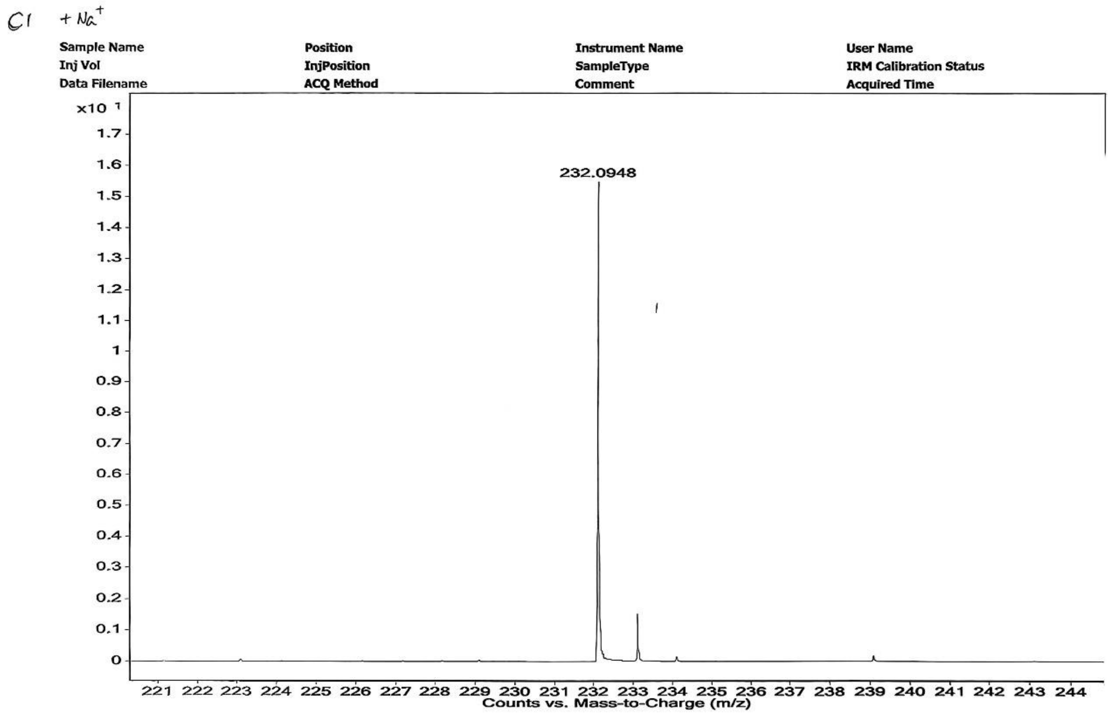

Figure S43. TOF-HRMS (ESI) spectra of compound **3a**.

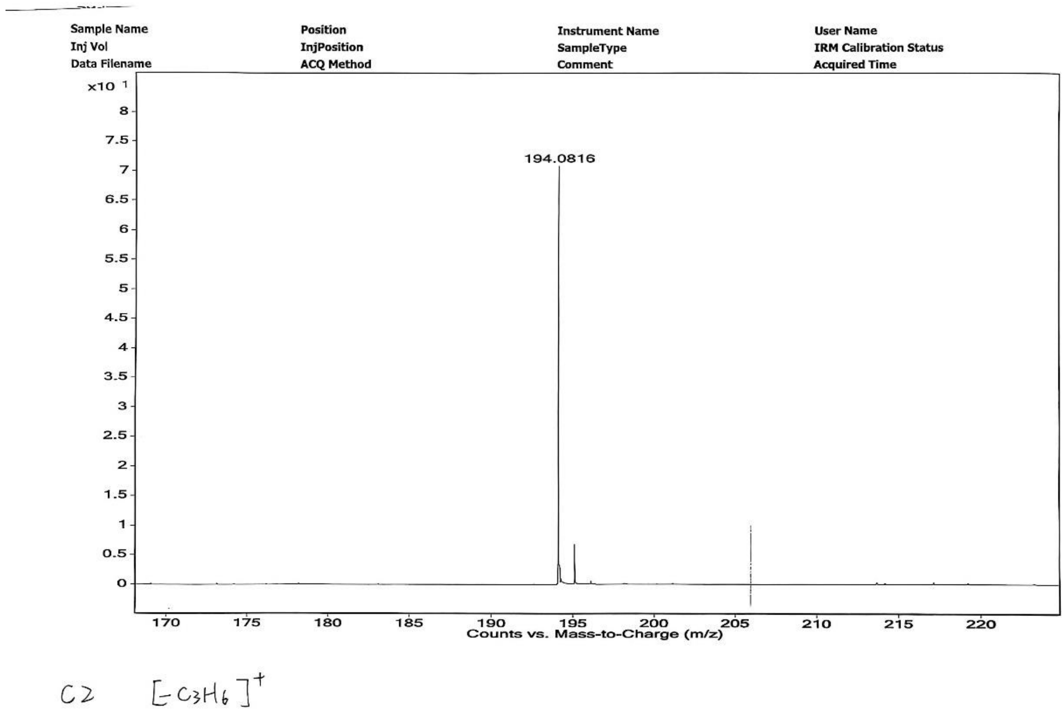

Figure S44. TOF-HRMS (ESI) spectra of compound **3b**.

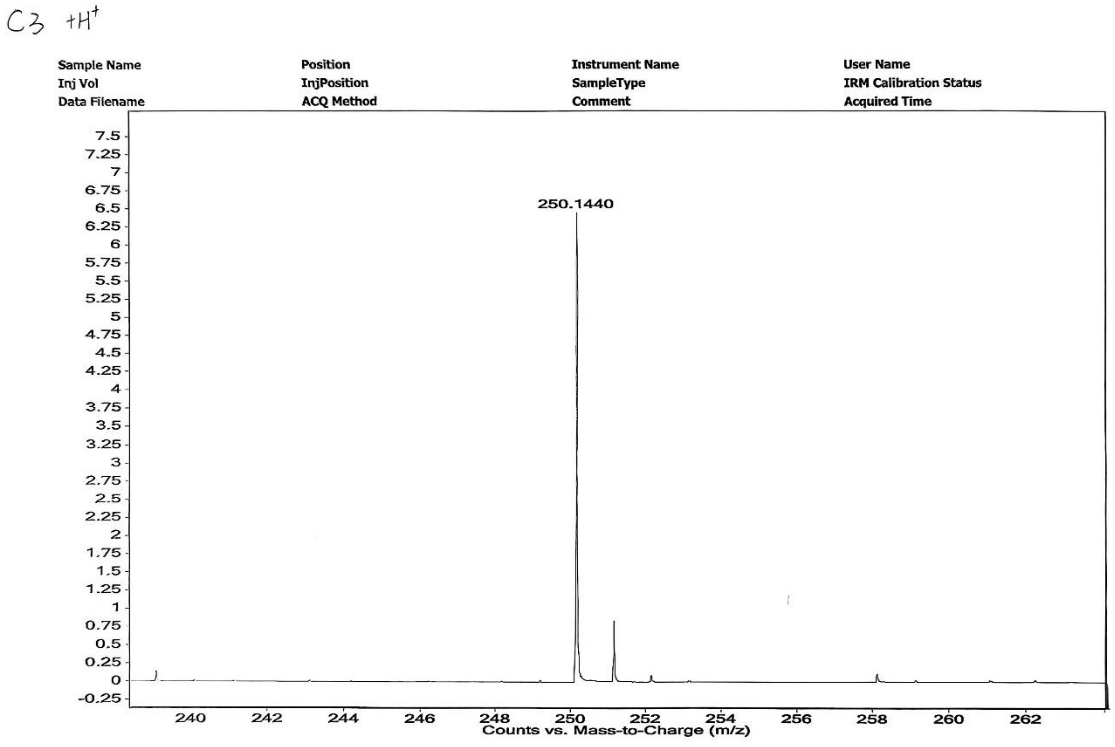

Figure S45. TOF-HRMS (ESI) spectra of compound **3c**.

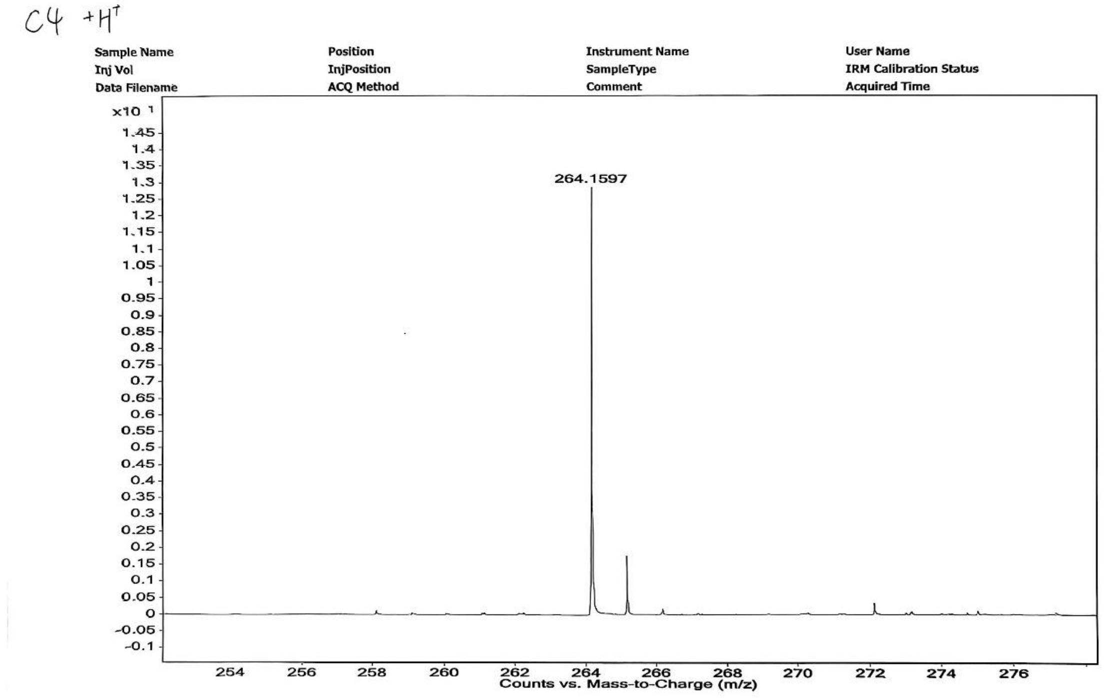

Figure S46. TOF-HRMS (ESI) spectra of compound **3d**.

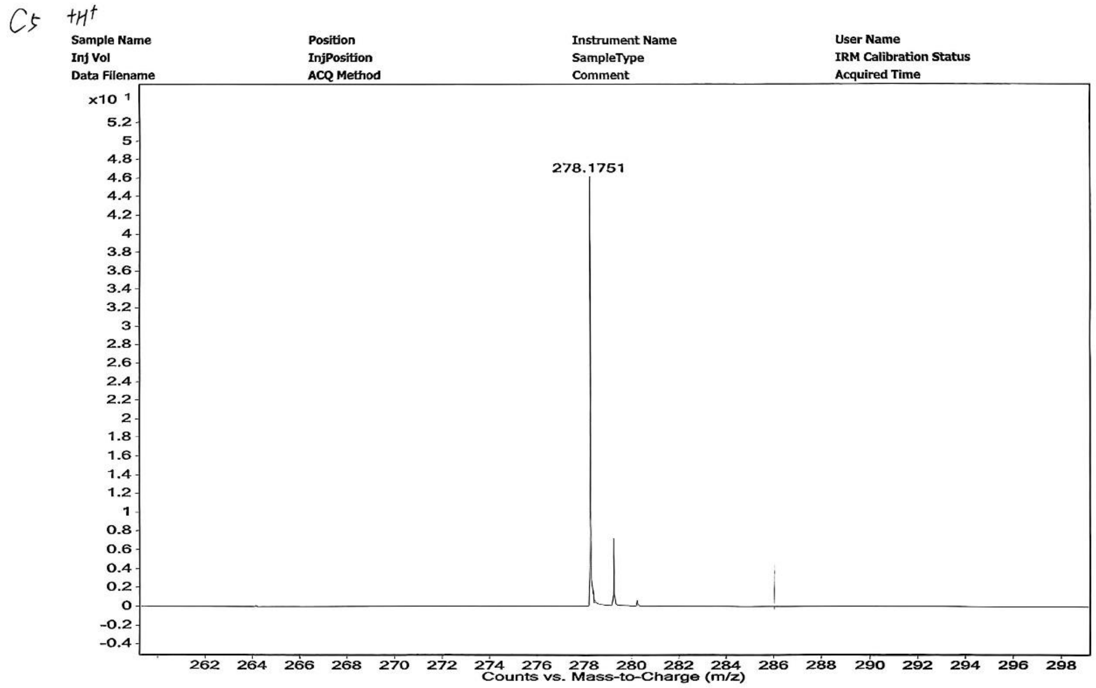

Figure S47. TOF-HRMS (ESI) spectra of compound **3e.**

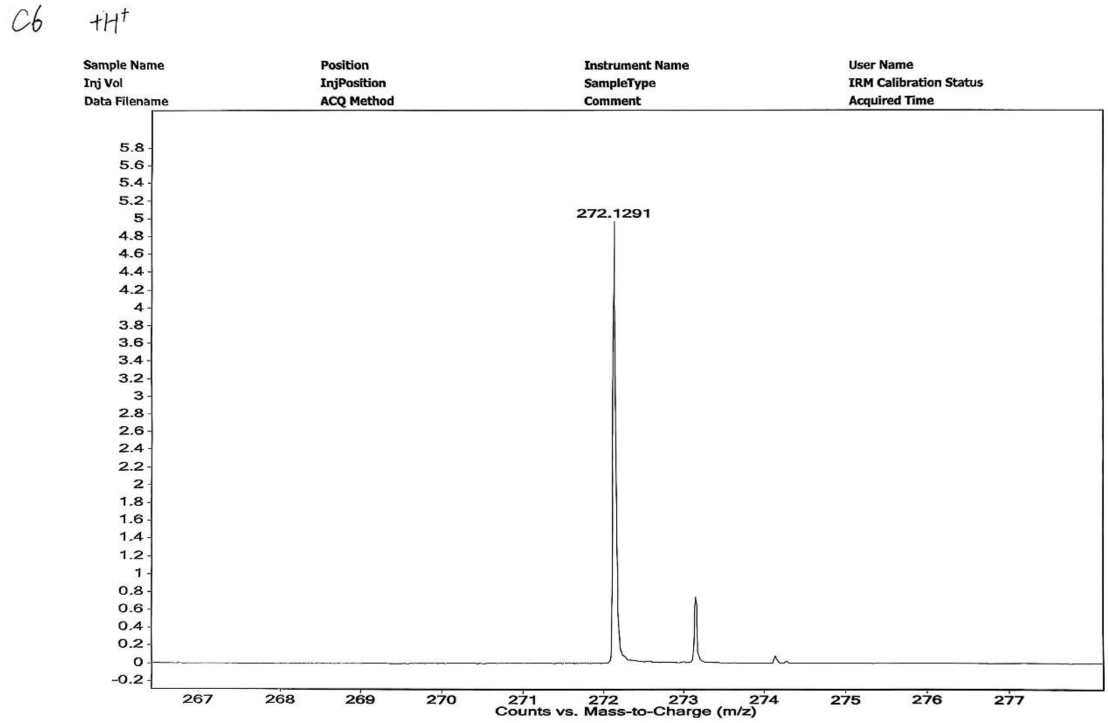

Figure S48. TOF-HRMS (ESI) spectra of compound **3f**.

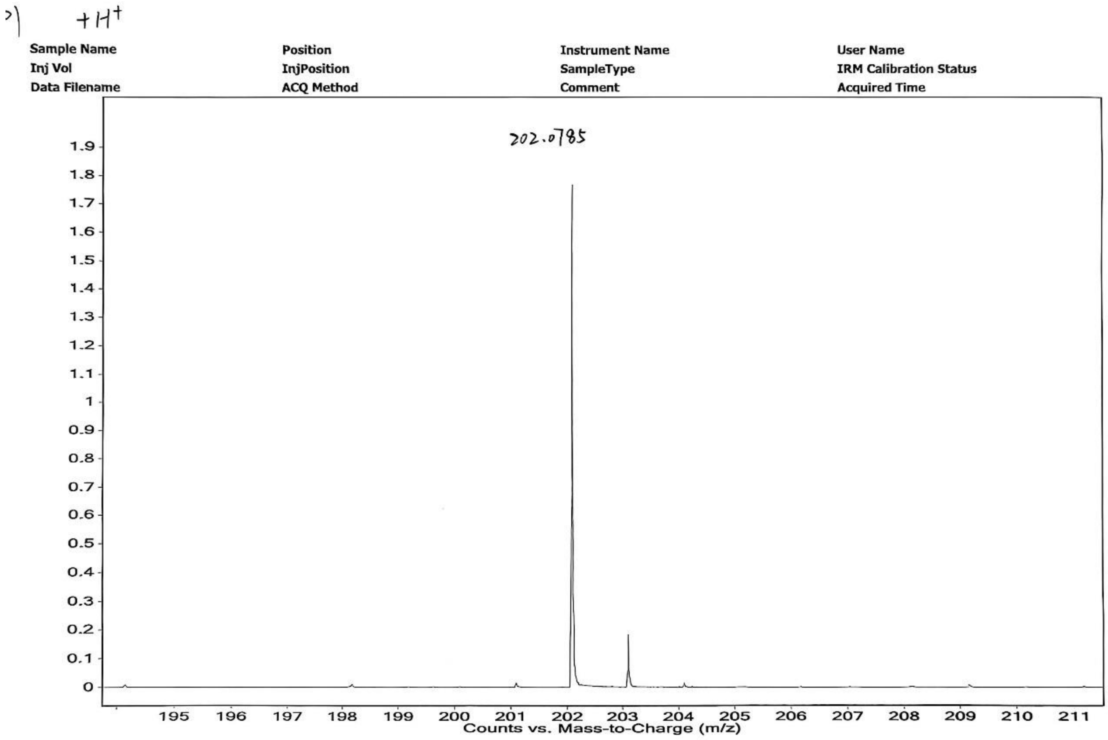

Figure S49. TOF-HRMS (ESI) spectra of compound **4a**.

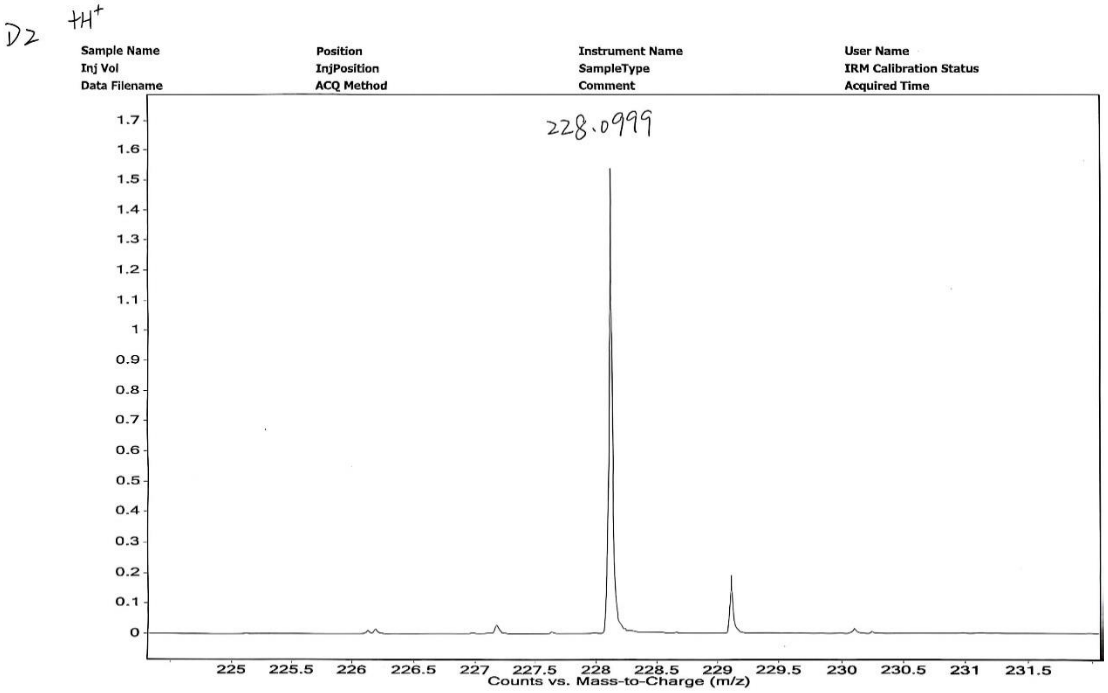

Figure S50. TOF-HRMS (ESI) spectra of compound **4b**.

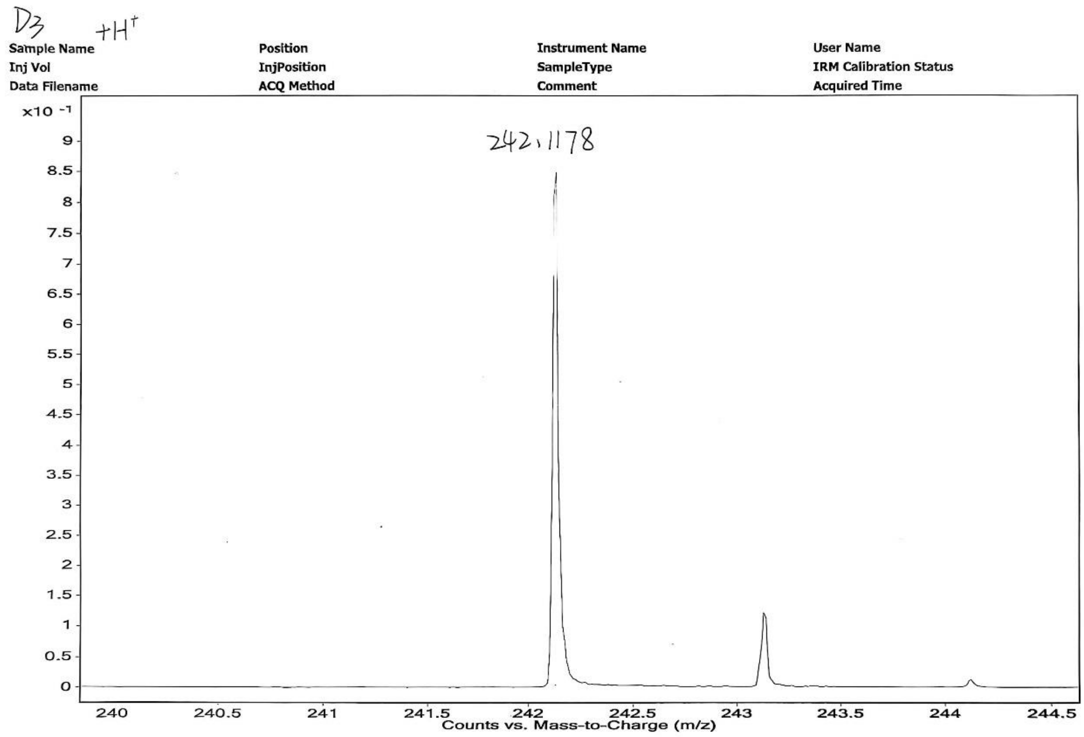

Figure S51. TOF-HRMS (ESI) spectra of compound **4c**.

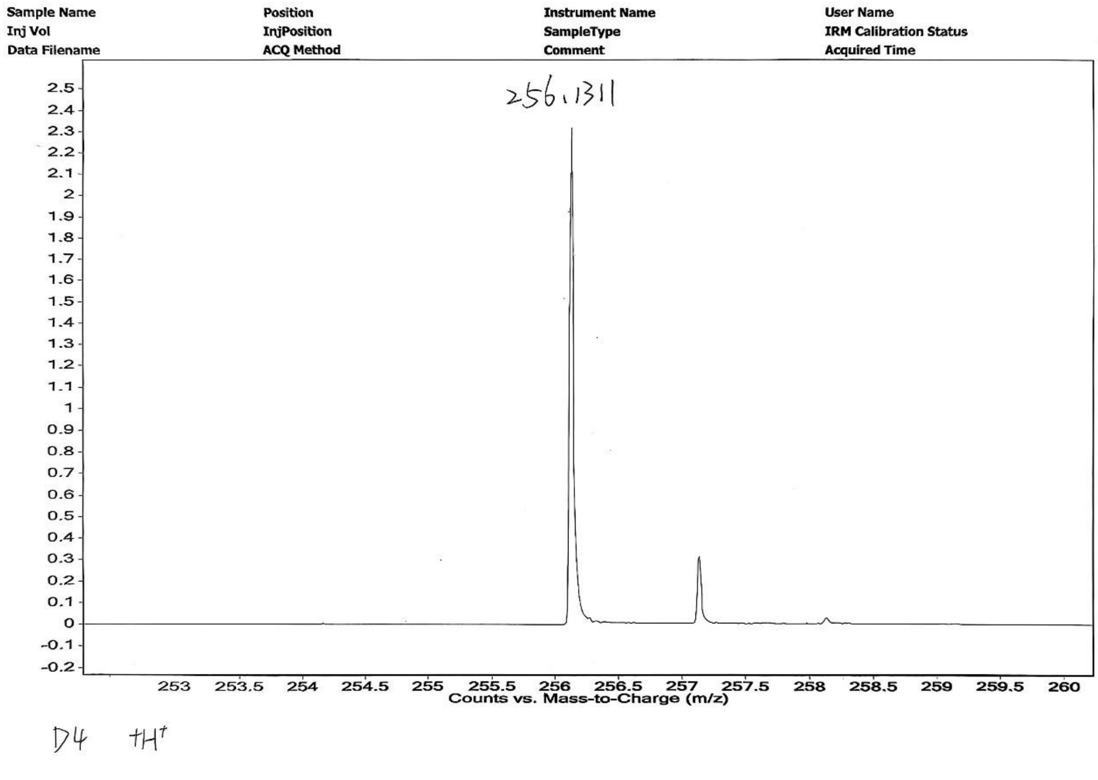

Figure S52. TOF-HRMS (ESI) spectra of compound **4d**.

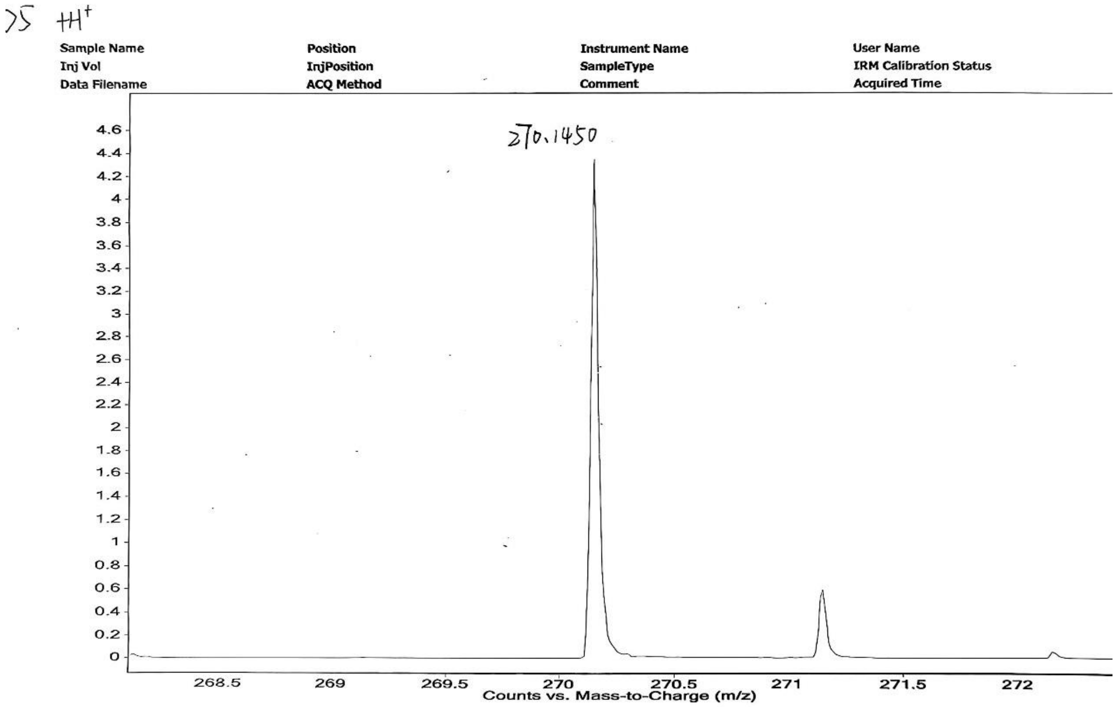

Figure S53. TOF-HRMS (ESI) spectra of compound **4e**.

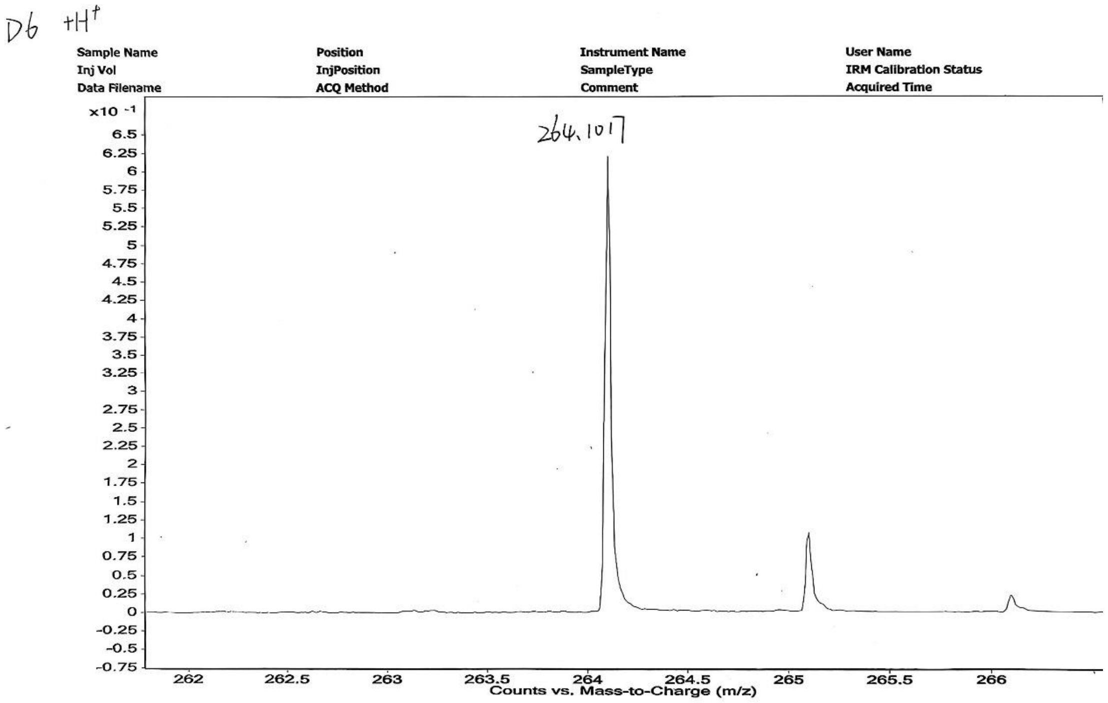

Figure S54. TOF-HRMS (ESI) spectra of compound **4f**.

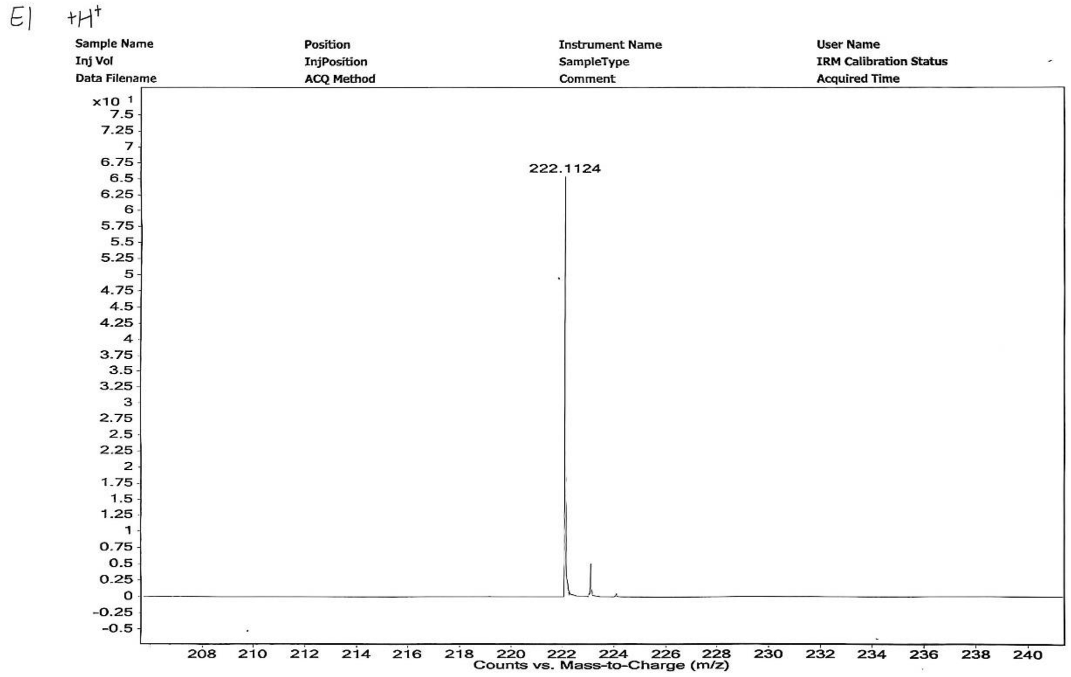

Figure S55. TOF-HRMS (ESI) spectra of compound **5a**.

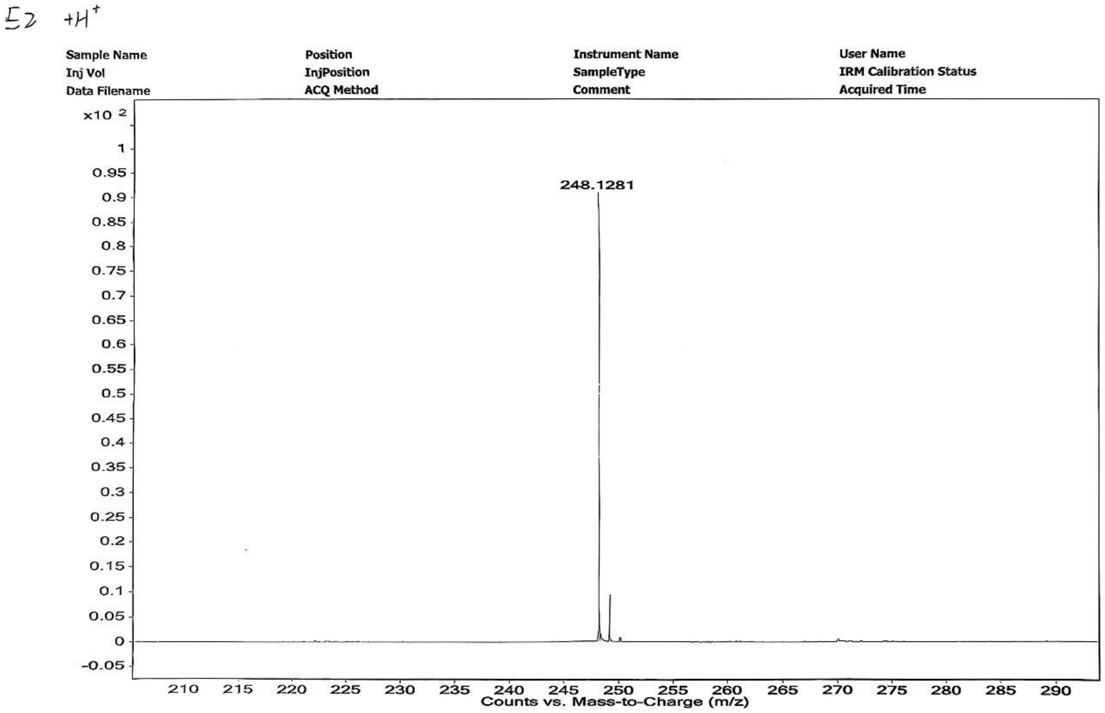

Figure S56. TOF-HRMS (ESI) spectra of compound **5b.**

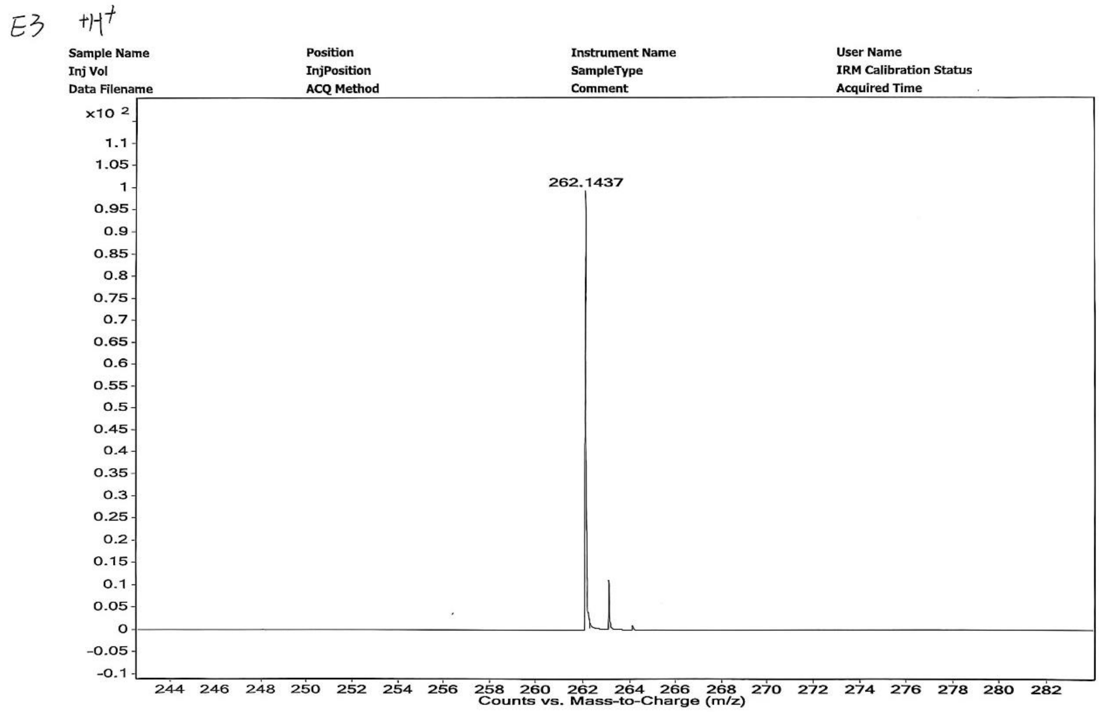

Figure S57. TOF-HRMS (ESI) spectra of compound **5c**.

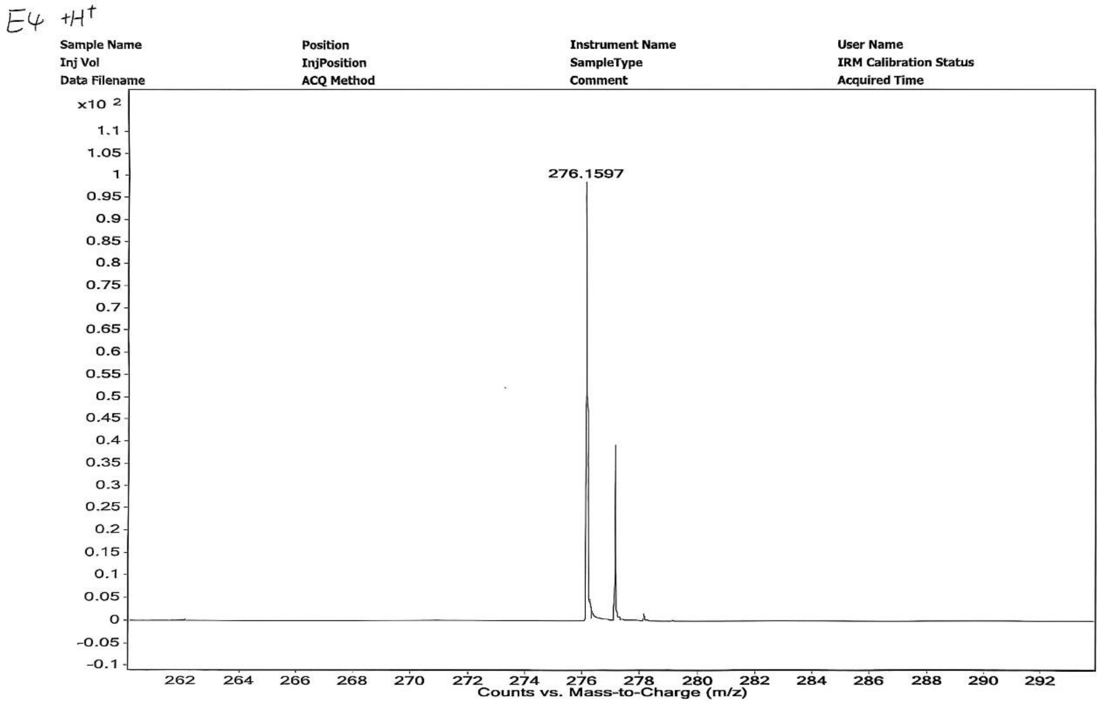

Figure S58. TOF-HRMS (ESI) spectra of compound **5d**.

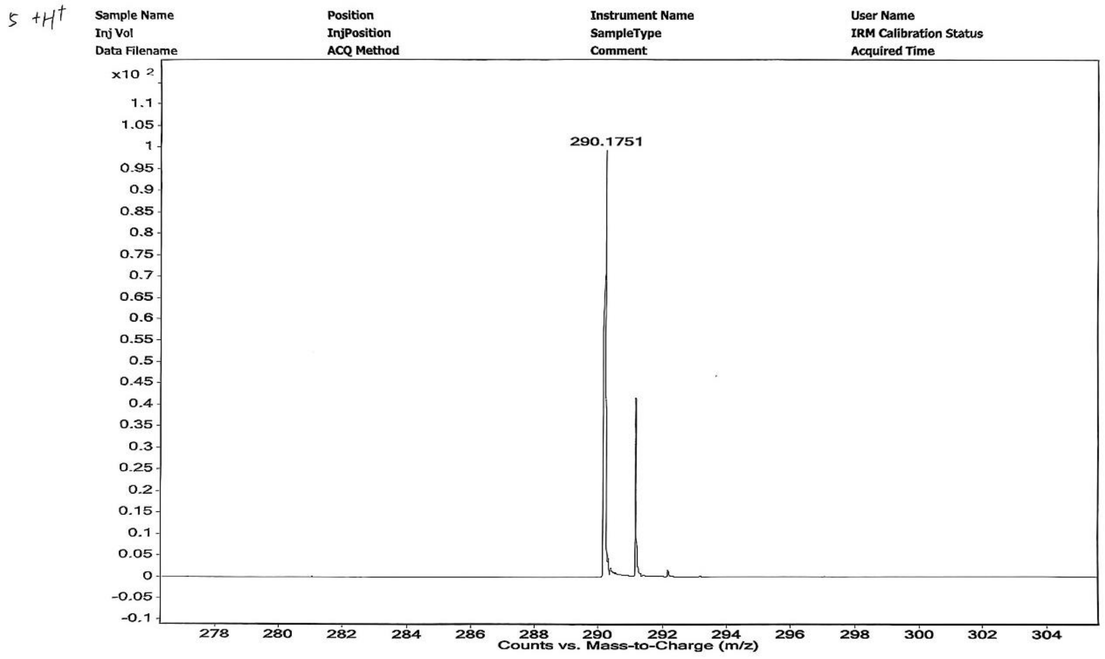

Figure S59. TOF-HRMS (ESI) spectra of compound **5e**.

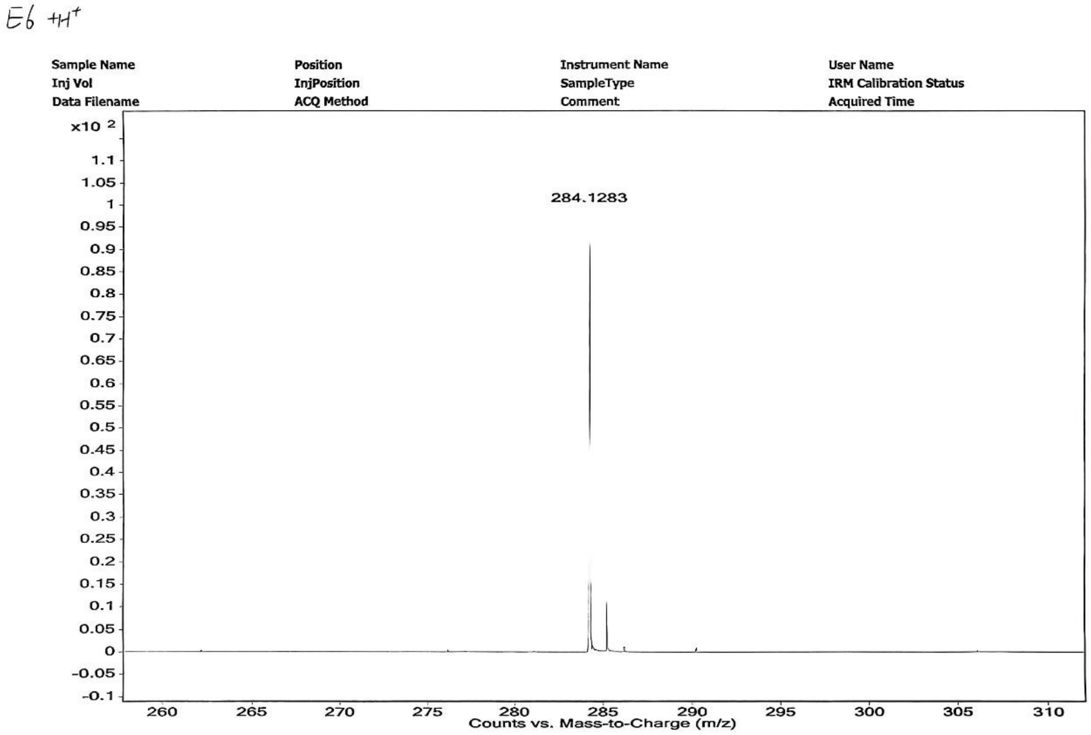

Figure S60. TOF-HRMS (ESI) spectra of compound **5f**.
